# 2-Phenoxy-3-Trichloromethylquinoxalines Are Antiplasmodial Derivatives with Activity against the Apicoplast of *Plasmodium falciparum*

**DOI:** 10.3390/ph14080724

**Published:** 2021-07-26

**Authors:** Dyhia Amrane, Christophe-Sébastien Arnold, Sébastien Hutter, Julen Sanz-Serrano, Miguel Collia, Amaya Azqueta, Lucie Paloque, Anita Cohen, Nadia Amanzougaghene, Shahin Tajeri, Jean-François Franetich, Dominique Mazier, Françoise Benoit-Vical, Pierre Verhaeghe, Nadine Azas, Patrice Vanelle, Cyrille Botté, Nicolas Primas

**Affiliations:** 1 Aix Marseille Univ, CNRS, ICR UMR 7273, Equipe Pharmaco-Chimie Radicalaire, Faculté de Pharmacie, CEDEX 05, 13385 Marseille, France; dyhia.amrane@etu.univ-amu.fr; 2 ApicoLipid Team, Institute for Advanced Biosciences, Université Grenoble Alpes, 38700 La Tronche, France; arnold.csa@gmail.com; 3 Aix Marseille Univ, IHU Méditerranée Infection, UMR VITROME, IRD, SSA, Mycology & Tropical Eucaryotic Pathogens, CEDEX 05, 13005 Marseille, France; sebastien.hutter@univ-amu.fr (S.H.); anita.cohen@univ-amu.fr (A.C.); nadine.azas@univ-amu.fr (N.A.); 4 Department of Pharmacology and Toxicology, Faculty of Pharmacy and Nutrition, University of Navarra, C/Irunlarrea 1, 31008 Pamplona, Spain; jsanz.3@alumni.unav.es (J.S.-S.); mcollia@alumni.unav.es (M.C.); amazqueta@unav.es (A.A.); 5 Navarra Institute for Health Research, IdiSNA, Irunlarrea 3, 31008 Pamplona, Spain; 6 LCC-CNRS, Université de Toulouse, CNRS UPR8241, UPS, 31400 Toulouse, France; lucie.paloque@lcc-toulouse.fr (L.P.); Francoise.Vical@inserm.fr (F.B.-V.); pierre.verhaeghe@lcc-toulouse.fr (P.V.); 7 MAAP, New Antimalarial Molecules and Pharmacological Approaches, MAAP, Inserm ERL 1289, 31400 Toulouse, France; 8 Institut de Pharmacologie et de Biologie Structurale, IPBS, Université de Toulouse, CNRS, UPS, 31400 Toulouse, France; 9 Sorbonne Université, INSERM, CNRS, Centre d’Immunologie et des Maladies Infectieuses, CIMI, 75013 Paris, France; amanzougaghene_nadia@yahoo.fr (N.A.); tajeri.shahin@inserm.fr (S.T.); jean-francois.franetich@upmc.fr (J.-F.F.); dominique.mazier@sorbonne-universite.fr (D.M.); 10 CHU de Toulouse, Service Pharmacie, 330 Avenue de Grande-Bretagne, CEDEX 9, 31059 Toulouse, France; 11 APHM, Hôpital Conception, Service Central de la Qualité et de l’Information Pharmaceutiques, 13005 Marseille, France

**Keywords:** quinoxaline, trichloromethyl goup, *Plasmodium falciparum*, structure-activity relationships, apicoplast

## Abstract

The malaria parasite harbors a relict plastid called the apicoplast. Although not photosynthetic, the apicoplast retains unusual, non-mammalian metabolic pathways that are essential to the parasite, opening up a new perspective for the development of novel antimalarials which display a new mechanism of action. Based on the previous antiplasmodial hit-molecules identified in the 2-trichloromethylquinoxaline series, we report herein a structure–activity relationship (SAR) study at position two of the quinoxaline ring by synthesizing 20 new compounds. The biological evaluation highlighted a hit compound (**3i**) with a potent *Pf*K1 EC_50_ value of 0.2 µM and a HepG2 CC_50_ value of 32 µM (Selectivity index = 160). Nitro-containing (**3i**) was not genotoxic, both in the Ames test and in vitro comet assay. Activity cliffs were observed when the 2-CCl_3_ group was replaced, showing that it played a key role in the antiplasmodial activity. Investigation of the mechanism of action showed that **3i** presents a drug response by targeting the apicoplast and a quick-killing mechanism acting on another target site.

## 1. Introduction

Malaria remains the deadliest parasitic disease; according to the World Health Organisation (WHO) [1], malaria caused an estimated 229 million cases leading to 409,000 deaths in 2019 with 94% of malaria cases and deaths occurring in the sub-Saharan Africa region. The most lethal species infecting humans is *Plasmodium falciparum*. The parasite is transmitted to humans by the bite of an infected female *Anopheles* mosquito. Currently, the first-line treatment of malaria caused by *P. falciparum* is based on artemisinin-based combination therapies (ACTs). Unfortunately, despite global efforts, the emergence of parasite resistance to the most effective class of antimalarial drugs, such as artemisinin (ART) [2], has led to treatment failures, particularly in the Greater Mekong Subregion [3]. However, the geographic scope of resistance may be rapidly expanding. Indeed, molecular markers of artemisinin resistance were described in African regions, leading to a significant public health concern [4]. Moreover, there are only six new chemical entities in the Medicines for Malaria Ventures (MMV) clinical pipeline [5].

Therefore, there is an urgent need for new therapies with novel mechanisms of action against *Plasmodium* in order to reduce the risk of emerging resistant parasites and to ensure the sustained efficacy of ACTs.

**Figure 1:**
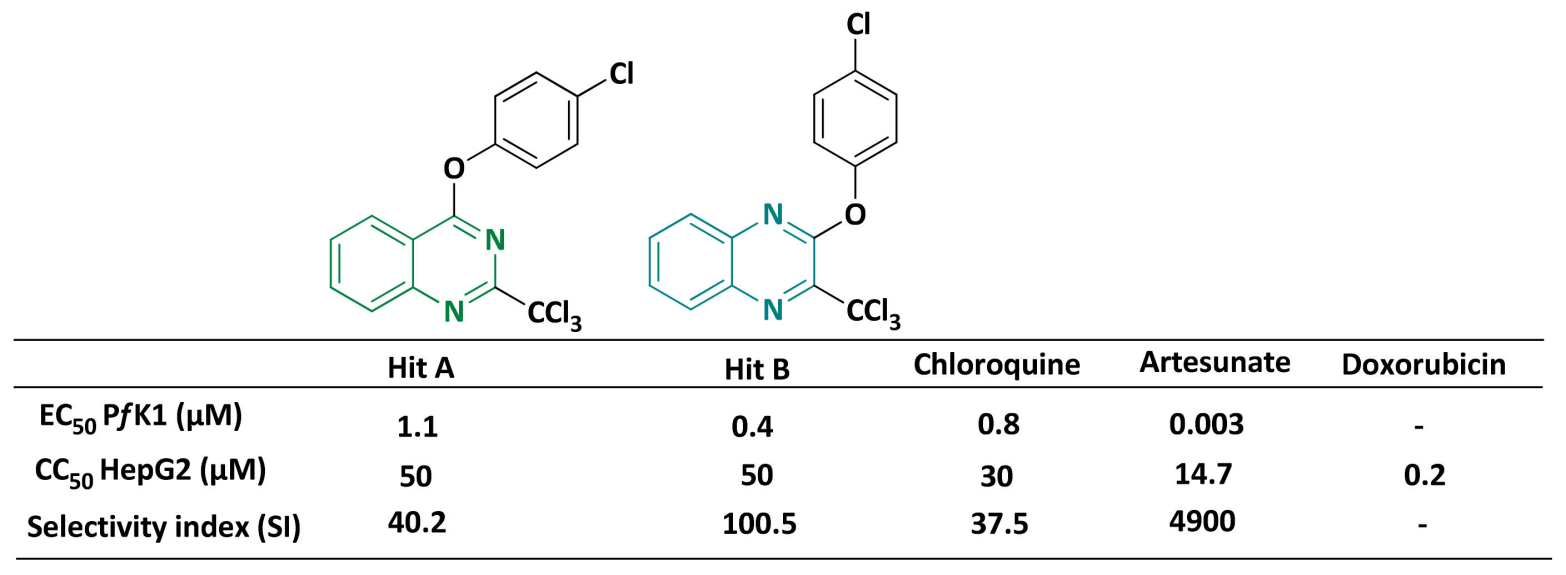
Comparison of biological activities between hit **A,** hit **B** and reference drugs.

Focusing on the development of new anti-infective agents, our team has reported the antiplasmodial activities of nitrogenous heterocyclic scaffolds bearing a trichloromethyl (CCl_3_) group, including a hit molecule (**A**) in quinazoline series (Figure 1) [6,7,8]. This hit-compound showed a good antiplasmodial profile (EC_50_ *Pf*K1 = 1.1 µM and CC_50_ = 50 µM) [9]. Furthermore, replacement of the quinazoline ring by a quinoxaline one (hit **B**, Figure 1), using a scaffold-hopping strategy, improved the antiplasmodial activity and preserved the cytotoxicity profile compared to hit **A**.

**Figure 2:**
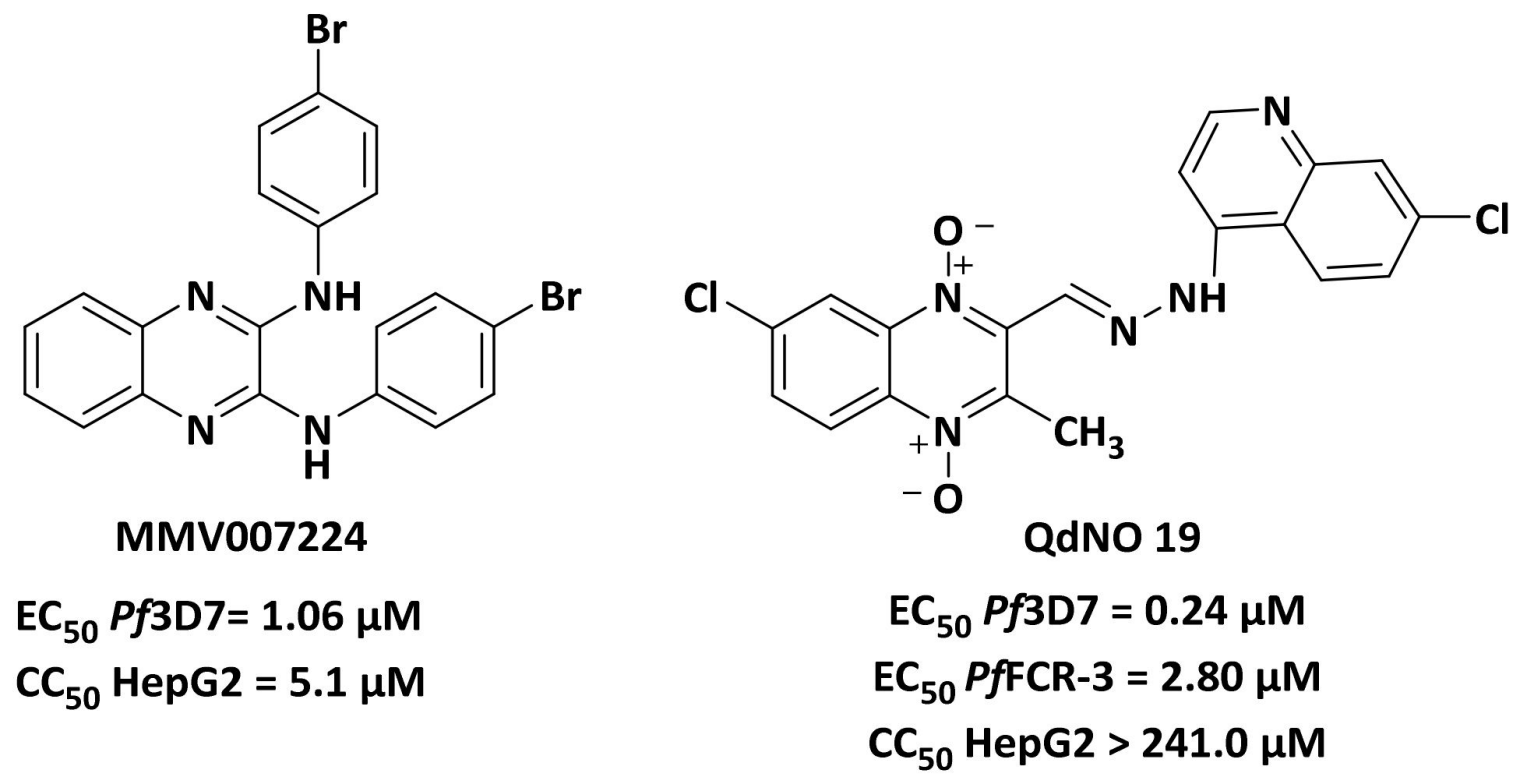
Structures of quinoxalines with antiplasmodial activities.

Indeed, several quinoxalines have already been described as promising antiplasmodial molecules, including **MMV007224** (Figure 2), which was identified from the Malaria Box [10]. In addition, the 1,4-di-*N*-oxide quinoxaline derivative (**QdNO 19**) showed antiplasmodial activity against both the 3D7 chloroquine-sensitive and FCR-3 multidrug-resistant strains of *P. falciparum* [11].

The MMV has defined requirements for the target candidate profiles (TCP) and target product profiles (TPP) [12]. One way to achieve these goals is to search for compounds with new mechanisms of action. Among antiplasmodial targets identified, the apicoplast, a relic plastid of Apicomplexa, constitutes an interesting target for the development of new antimalarial drugs.

Apicomplexan parasites are responsible for serious infectious diseases, such as malaria. Most apicomplexans have a key organelle called the apicoplast, which is a non-photosynthetic plastid acquired by a secondary endosymbiosis from a plastid-containing red alga [13,14]. This organelle contains the second smallest known circular genome for a plastid (35 kilobases) which encodes for proteins that are targeted to the apicoplast [15]. Although not photosynthetic, the apicoplast performs several essential anabolic functions for the parasite, including isoprenoid precursors biosynthesis, fatty acids biosynthesis, heme biosynthesis, and iron-sulfur cluster assembly [16].

To date, very few molecules targeting the apicoplast have been described, however, some known antibiotics have been studied, such as ciprofloxacin and doxycycline, which target the genetic machinery of *P. falciparum* apicoplast leading to a delayed death drug-response [17]. Clindamycin and azithromycin are other examples of delayed death antibiotics that target *P. falciparum* [18].

The vestigial origin of the apicoplast has paved the way for innovative antimalarial drugs that can be described as herbicidal therapies. Among the biocides targeting the apicoplast, triclosan showed antiparasitic activity against *Plasmodium* through the fatty acid biosynthesis pathway inhibition located in the apicoplast [19], although it was off-target and not active in vivo [20].

**Figure 3:**
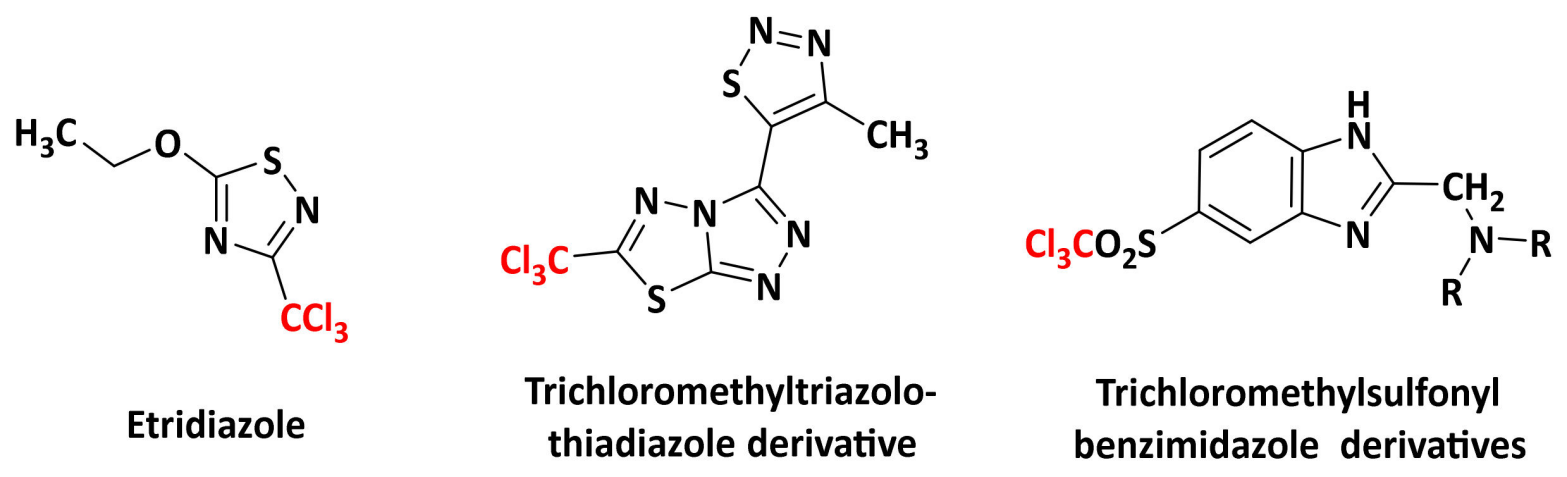
Structures of some trichloromethylated derivatives as biocides.

It is noteworthy that some trichloromethylated molecules were known as biocides, such as etridiazole (Figure 3) [21] and trichloromethyltriazolothiadiazole, which target pyruvate kinase II located in the apicoplast [22]. In addition, Banach et al. described trichloromethylsulfonylbenzimidazole derivatives with herbicidal activities targeting the apicoplast [23]. Therefore, a possible correlation between the pharmacophore -CCl_3_ and herbicidal activity could be suggested.

All these data allowed us to establish a relationship between the biocid effect of the CCl_3_ group and the antiplasmodial activities of herbicides that target the apicoplast. In order to decipher the mechanism of action of our trichloromethylated molecules, studies to determine the effect of our molecules on apicoplast biogenesis during the blood and hepatic stages of *Plasmodium falciparum* are discussed here. In parallel, the action against artemisinin-resistant parasites of *P. falciparum* was also studied.

## 2. Results and Discussion

### 2.1. Synthesis

We planned to explore the structure–activity relationship by investigating the influence of substituents at position 2 of the 3-trichloromethylquinoxaline scaffold, in a view to identify new optimized hit-compounds.

**Scheme 1:**
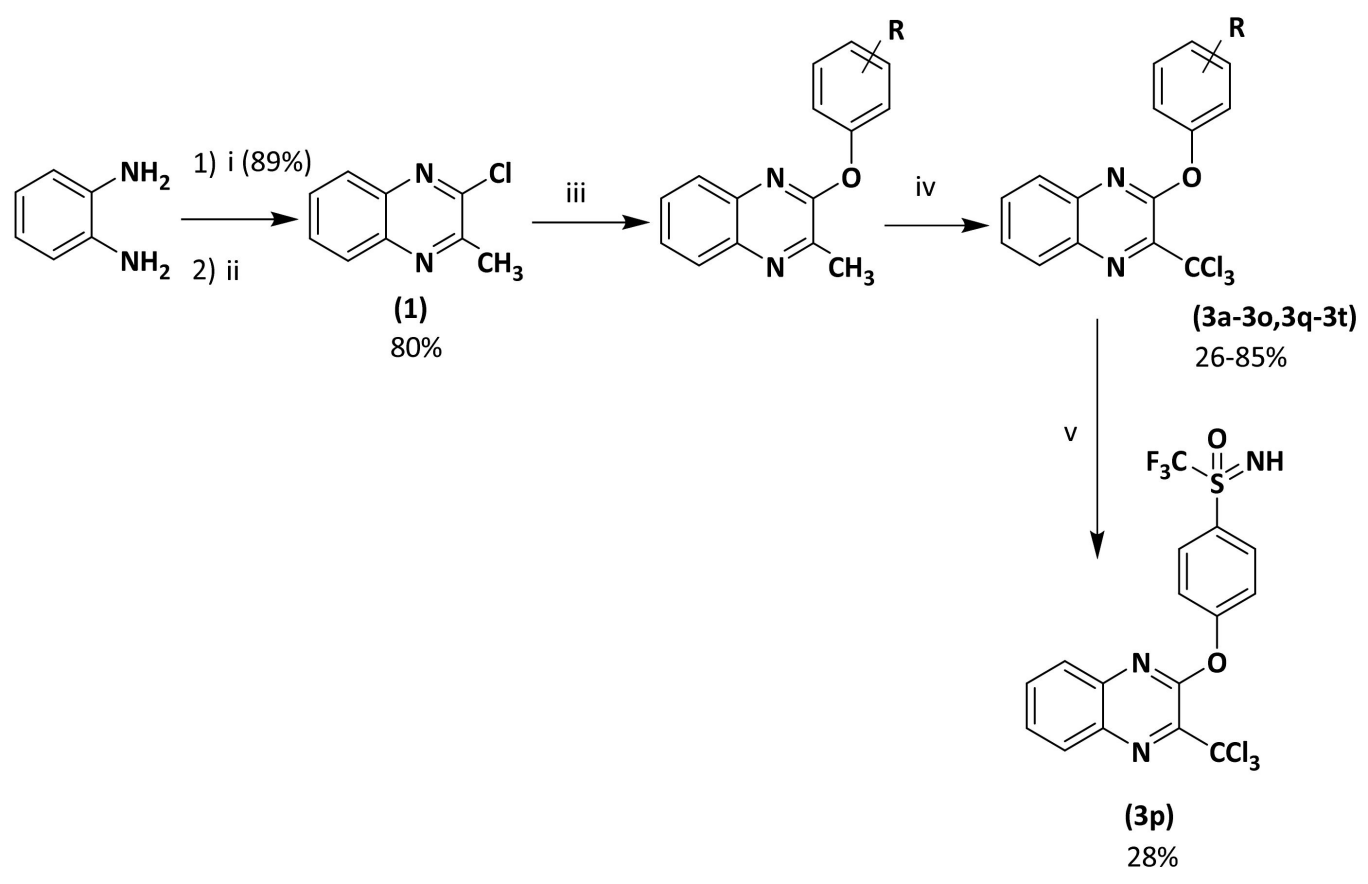
Preparation of 2-phenoxy-3-trichloromethylquinoxaline derivatives (**3a–3t**). Reagents and conditions: (i) ethyl pyruvate 1 equiv, H_2_O, 50 °C, 15 min; (ii) POCl_3_, reflux, 2 h; (iii) appropriate phenol derivative 1 equiv, Cs_2_CO_3_ 1 equiv, anh. DMF, 70 °C, 12 h, sealed vial, N_2_; (iv) PCl_5_ 6 equiv, POCl_3_ as solvent, 100 °C, MW, 20 min, 800 W; (v) (**3m**) 1 equiv, ammonium carbamate 1.5 equiv, phenyliodine diacetate 2.1 equiv., CF_3_CH_2_OH, rt, 3 h.

Key substrate (**1**) was obtained from a condensation reaction of o-phenylenediamine with ethyl pyruvate to provide the corresponding lactam followed by a chlorination reaction using POCl_3_ [8] (Scheme 1). Next, chlorimine (**1**) was reacted by a nucleophilic aromatic substitution reaction (S_N_Ar) with various phenol derivatives using cesium carbonate in DMF at 70 °C. The desired intermediates (**2a–2t**) were obtained in moderate to quantitative yields (45 to 99%). Yield variation seems to have no clear correlation with the electron-donating or -withdrawing behavior of the different substituents borne on the phenols. Finally, according to a previously described protocol [24], from the methylated precursors (**2a–2t**), a chlorination reaction using PCl_5_ and POCl_3_ was performed under microwave heating at 100 °C for 30 min, leading to the target analogs of hit **B** (**3a–3t**) in low to very good yields (26–85%). Globally, 20 derivatives (**3a–3t**) were obtained: 16 compounds bearing electro-donating or electro-withdrawing substituents in ortho, meta, and para positions of the phenol moiety, and four cluttered phenol derivatives.

### 2.2. Biological Results

#### 2.2.1. Structure-Activity Relationship (SAR) Study

These new derivatives were evaluated in vitro against the K1 chloroquine and pyrimethamine-resistant *P. falciparum* strain by determining their 50% efficacy concentration (EC_50_), and were compared to three reference antimalarial drugs: chloroquine, artesunate, and doxycycline. The in vitro 50% cytotoxic concentrations (CC_50_) were assessed on the HepG2 human hepatocyte cell line and compared to a cytotoxic reference drug, doxorubicin. For all molecules, the selectivity index was calculated as following SI = CC_50_/EC_50_. The results are presented in Table 1 and Table 2.

Out of the 20 tested molecules, five showed poor aqueous solubility in the cell culture medium (**3e, 3h, 3r, 3s, 3t**), limiting their in vitro cytotoxicity evaluation. Regarding the effect on cell viability of the HepG2 human cells, except for two compounds (**3o** and **3p**), all derivatives showed good cytotoxicity values ranging from 15.4 to 53.1 µM in comparison with reference drug doxorubicin (CC_50_ HepG2 = 0.20 µM). It appeared that the cluttered derivatives (**3q**, **3s**) and the 2-chlorophenoxy derivative (**3b**) were the less cytotoxic compounds. The electron-withdrawing groups (EWG) (**3l, 3n**) at *para* position of 3-phenoxy moiety led to slightly more cytotoxic compounds ((CC_50_ HepG2 = 15.4 and 16.6 µM, respectively).

The *meta*-halogenated (chloro or fluoro) phenoxy group (**3c** and **3f**) gave the best antiplasmodial activities with quite similar activities ((EC_50_ *Pf* K1 = 0.34 and 0.32 µM, respectively) compared to the ortho (**3b, 3e**) and para (**3g**) positions.

The *para*-EWG as 4-OCF_3_ (**3k**) and the *para*-electro-donating group (EDG) as OMe (**3h**) remained active as the halogenated phenoxy moieties (EC_50_ *Pf* K1 = 0.40 and 0.38 µM, respectively). The unsubstituted phenoxy group (**3a**) also showed good antiplasmodial activity. Among the cluttered analogs, the 2-(naphthalen-2-yloxy) was slightly better than the 2-(naphtalen-1-yloxy) compound (EC_50_ *Pf* K1 = 0.40 vs. 0.60 µM). Strong EWG such as 4-SF_5_, 4-SCF_3_, and 4-CN (**3l**, **3m**, **3n**) afforded the best antiplasmodial activities (EC_50_ *Pf* K1 = 0.20 µM for each molecule).

From the trifluoromethylthio derivative (**3m**), in order to add H-bond donor and decrease clogP, an analog bearing a trifluoromethylsulfoximine at *para* position was synthetized. Moreover, to anticipate the potential in vivo sulfur oxidation of (**3m**), the sulfonyl **3o** was prepared. These two analogs (**3o, 3p**) showed both good antiplasmodial activity. However, these were the most cytotoxic derivatives.

Finally, the best compound in this series is the 4-NO_2_-substituted phenoxy group (**3i**) which showed both good EC_50_ and CC_50_ values, leading to the highest selectivity index in the series (SI = 160.0) (Table 1 and Table 2). It is also notable that the best compound has also the lowest clogP.

#### 2.2.2. In Vitro Toxicity Data

Toxicity concerns related to nitro-containing drugs emerged a long time ago [25], particularly due to the mutagenic potential of these compounds [26]. Most of molecules bearing a nitro group are well known for their mutagenicity against the *Salmonella typhimurium* strain used in the Ames test [27].

Nevertheless, the evaluation of the mutagenicity of compound (**3i**) was carried out using the Ames test on four *S. typhimurium* strains at 5 mM and 25 mM (four doses per concentrations: 2, 3, 4, 5 µL/plate for assay without S9 mix and 4, 6, 8, 10 µL/plate for assay with S9 mix). After 48 h of exposition of (**3i**), no significant number of revertants was observed compared to the positive control, indicating that this nitro-containing trichloromethylated molecule is not mutagenic in vitro (see Supplementary Materials).

To complete the mutagenic Ames test, in vitro comet assay was performed on the HepG2 cell line at the concentration of 3.2 and 16 µM (CC_50_ (**3i**)/2 and CC_50_ (**3i**)/10, 72 h) (Table 3). Compound **3i** did not induce DNA strand breaks or alkali labile sites after either short (2 h) or long (72 h) exposition at two concentrations tested (Supplementary Materials) Methyl methanesulphonate (MMS), the positive control used in the comet assay, gave the expected results.

#### 2.2.3. Role of the 3-CCl_3_ Group

**Figure 4:**
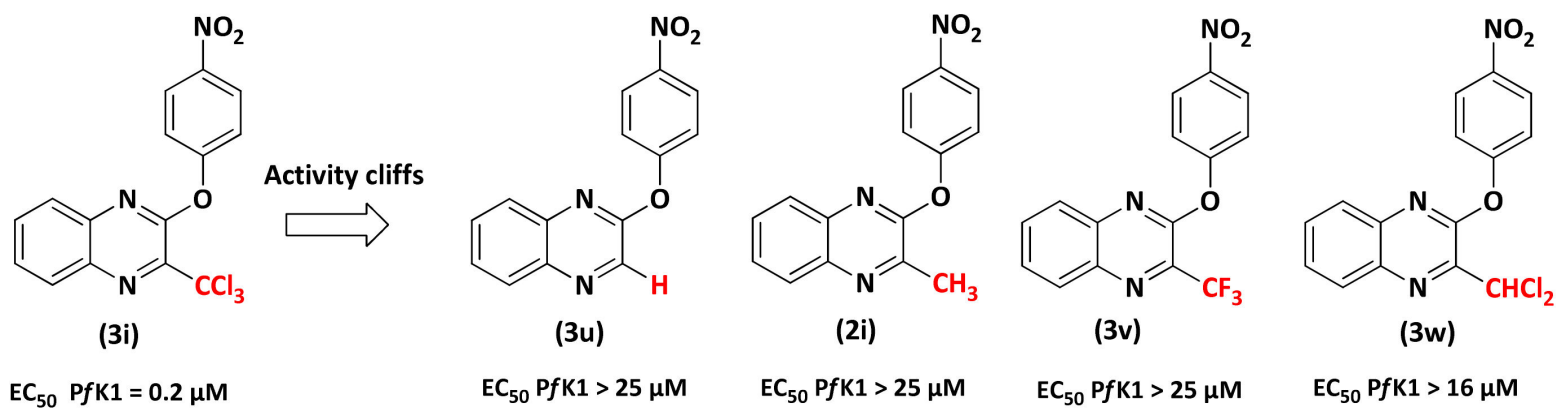
Comparison of the in vitro antiplasmodial activities of hit molecule **3i** with analogs **3u**, **3v**, **3w**, **3x**.

Previously, we have shown that the CCl_3_ group is mandatory to provide antiplasmodial activity [28,29,30]. To confirm the key role played by the 3-CCl_3_ group of the most potent compound (**3i**) in phenoxyquinoxaline series, the CCl_3_ group was replaced by a proton (**3u**), a CH_3_ group (**2i**), a CF_3_ group (**3v**), and a CHCl_2_ group (**3w**). All these analogs without the CCl_3_ group lost their activity against *P. falciparum* (EC_50_ *Pf*K1 > 16 µM). Once again, the CCl_3_ group appeared essential for antiplasmodial activity (Figure 4) [31].

#### 2.2.4. Evaluation on *P. falciparum* Apicoplast

**Figure 5:**
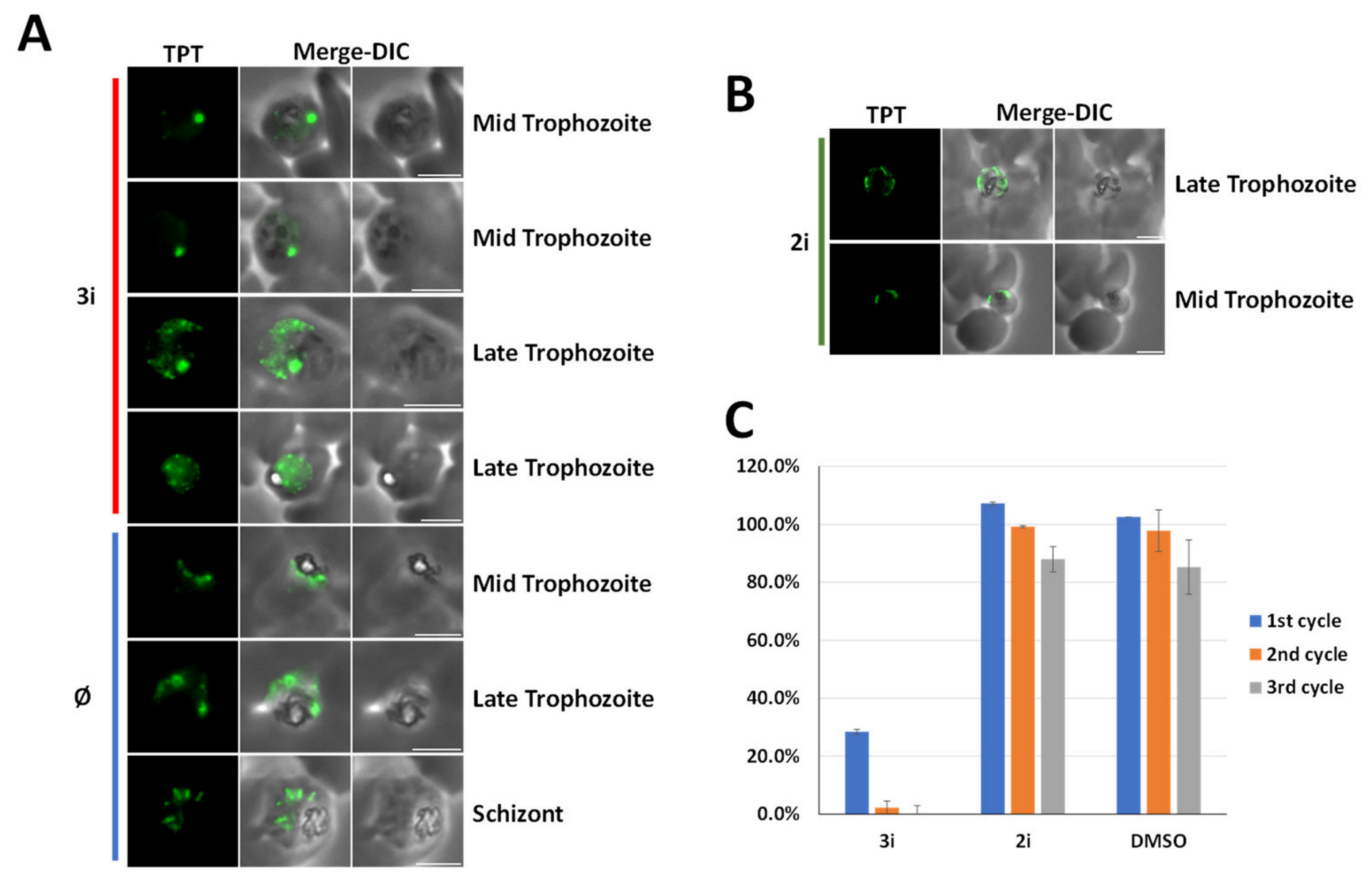
(**3i**) treatment affects apicoplast biogenesis. (**A**,**B**) A triose phosphate transporter (TPT) fluorescent signal is shown in green (TPT labeled with an 3 × HA tag). Apicoplasts of treated parasites with 0.2 µM of (**3i**) molecule or 0.2 µM of (**2i**) are compared to normal plastid visualized in Ø untreated control. Microscopic images were obtained by a epifluorescence microscope. DIC, differential interference contrast. (**C**) Growth assay over three life cycles (48 h), bars represent the ratio treated parasites on untreated. (*n* = 3, error bar = standard deviation, SD).

To determine the impact of (**3i**) on apicoplast biogenesis, parasites were treated for 48 h with 0.2 µM of (**3i**) or 0.2 µM of (**2i**) (a control of (**3i**) for which the active CCl_3_ was replaced by a methyl CH_3_ group) and compared to untreated parasites by immunofluorescence assay. In the untreated culture, parasites presented normal apicoplast biogenesis depending on the life stage (elongated for trophozoite stage and individualized for schizont) while treated culture with (**3i**) showed a doted single non-elongated apicoplast at the mid-trophozoite life stage and apicoplast, which seems to diffuse into the cytosol and form vesicles for advance life stages (i.e., late trophozoite) (Figure 5A). This result is similar to what was previously reported after parasite chemical rescue after apicoplast loss [32]. We confirmed the absence of effect of molecule (**2i**) on the apicoplast biogenesis and *Plasmodium* growth, correlated to the replacement of the CCl_3_ group of (**3i**) by CH_3_ (Figure 5B,C). In order to determine if the apicoplast is the main target of (**3i**), we performed a growth assay over three life cycles to assess a potential delayed death effect, one of the major signature for compounds affecting apicoplast. The results show a drastic reduction in the parasite growth within the first life cycle in the cultures treated with 0.2 µM of (**3i**) (Figure 5C), which can be interpreted in two ways: the first is that the primary target is outside of the apicoplast and the impact on apicoplast biogenesis is a collateral effect. The second interpretation could be that, unlike chloramphenicol or doxycycline, which cause a delayed death phenotype on *Plasmodium*, (**3i**) could act like fosmidomycin or actinonin and cause a rapid death phenotype [31]. Hence, although, we cannot exclude an indirect effect on the apicoplast, (**3i**) affects its presence and morphology.

#### 2.2.5. Evaluation on Artemisinin-Resistant and Artemisinin-Sensitive Parasites

It is essential to assess the efficacy of new antimalarial compounds against ART-resistance, as its worldwide spreading threats malaria control [1]. In this context, the ART-resistant strain F32-ART5 and its tween ART-sensitive strain F32-TEM were used [33,34]. Compound (**3i**) showed similar antiplasmodial activity on both strains F32-ART5 and F32-TEM with mean EC_50_ values of 400 nM and 392 nM, respectively (Mann–Whitney test *p*-value = 0.8). However, despite ART-resistant genotype of F32-ART5, EC_50_ values of ART were also similar for both strains (ranging from 11 to 19 nM, Mann–Whitney test p-value = 0.267) (Table 4), due to the specific *P. falciparum* quiescence-based mechanism of ART-resistance leading to parasite cell cycle arrest during drug exposure. Therefore, evaluation of (**3i**) with specific assays of artemisinin-resistance [33,34,35] was conducted and evidenced that this compound was active against the artemisinin-resistant parasites at the proliferative state but not at the quiescent state (see Supplementary Materials Figure S49).

#### 2.2.6. Studying the Effect of (**3i**) on the Liver Stage Development of *P. falciparum*

The effect of (**3i**) on the hepatic stage of *P. falciparum* was next studied in order to know if (**3i**) was active against another stage of *P. falciparum* cycle. Cryopreserved primary human hepatocytes were infected with 30,000 freshly extracted *P. falciparum* sporozoites and simultaneously treated with a dose range (0.9–30 µM) of (**3i**). Atovaquone (ATQ) was used as a positive parasite-killing control at 25 nM. Treatment with (**3i**) had no effect on exoerythrocytic forms (EEF or schizont) size and numbers at 6 or 12 days post-infection, while ATQ could completely clear all parasites from the culture (see Supplementary Materials Figures S50 and S51). The apicoplast in the hepatic stage was quite not affected by (**3i**) even at high concentrations (up to 30 µM) (see Supplementary Materials Figure S52).

**Figure 6:**
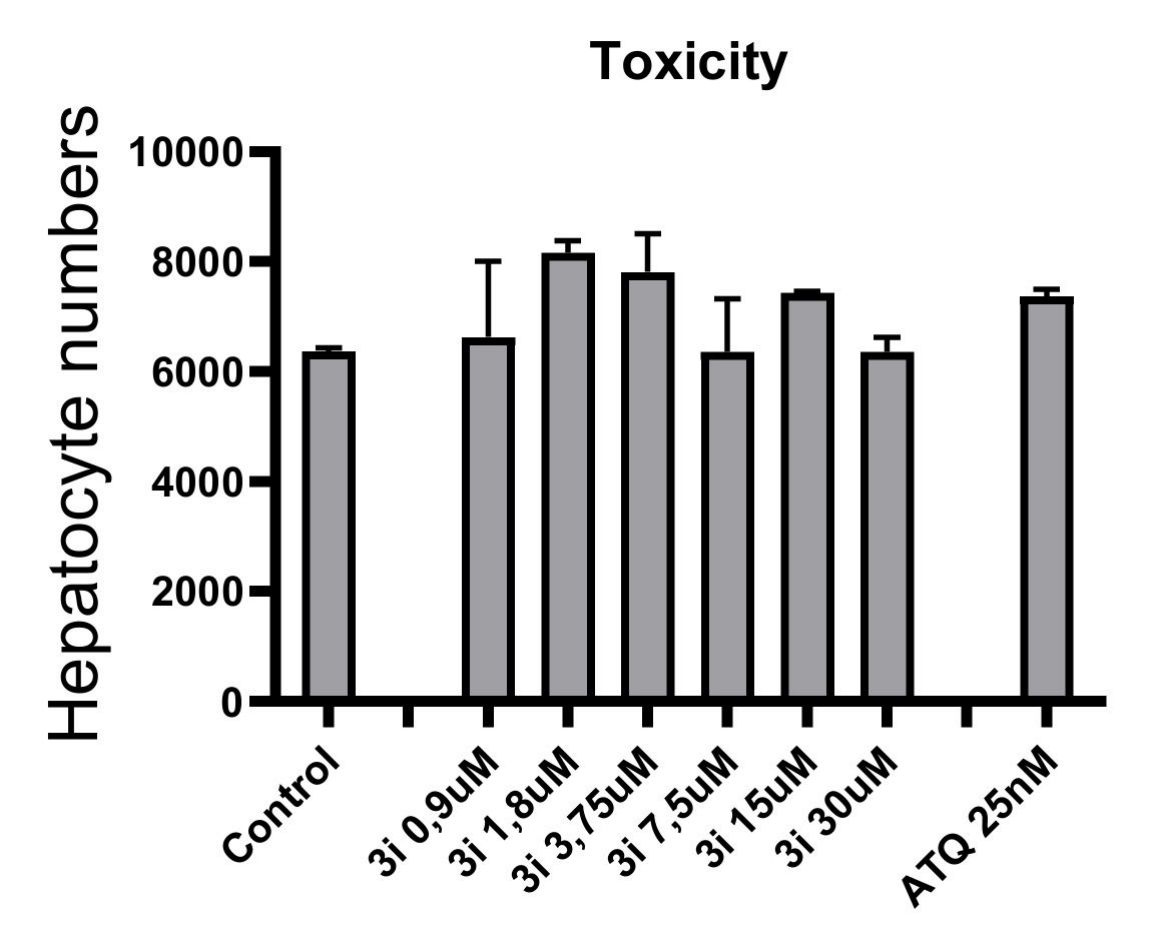
Toxicity of (**3i**) on human hepatocytes.

Finally, we studied the hepatotoxicity of (**3i**). This was carried out by quantification of DAPI-stained hepatocyte nuclei in culture wells. Generally, if a drug is hepatotoxic, it causes death and detachment of hepatocytes leading to less hepatocyte nuclei counts. The nuclei numbers in control and **3i**-treated wells are comparable and no reduction in hepatocyte numbers could be detected under the dose range tested (Figure 6). Interestingly, no cytotoxicity was observed on human hepatocytes up to 30 µM of (**3i**).

## 3. Materials and Methods

### 3.1. Chemistry

#### 3.1.1. Generality

Melting points were determined on a Köfler melting point apparatus (Wagner & Munz GmbH, München, Germany) and were uncorrected. Elemental analyses were carried out at the Spectropole, Faculté des Sciences de Saint-Jêrome (Marseille) with a Thermo Finnigan EA1112 analyzer (Thermo Finnigan, San Jose, CA, USA). NMR spectra were recorded on a Bruker AV (Billerica, MA, USA) 200 or AV 250 spectrometers or a Bruker Avance NEO 400MHz NanoBay spectrometer at the Faculté de Pharmacie of Marseille or on a Bruker Avance III nanobay 400 MHz spectrometer at the Spectropole, Faculté des Sciences de Saint-Jêrome (Marseille). (1H NMR: reference CHCl_3_ δ = 7.26 ppm, reference DMSO-d6 δ = 2.50 ppm and ^13^C NMR: reference CHCl_3_ δ = 76.9 ppm, reference DMSO-d_6_ δ = 39.52 ppm). The following adsorbent was used for column chromatography: silica gel 60 (Merck KGaA, Darmstadt, Germany, particle size 0.063–0.200 mm, 70–230 mesh ASTM). TLC was performed on 5 cm × 10 cm aluminum plates coated with silica gel 60F-254 (Merck) in an appropriate eluent. Visualization was performed with ultraviolet light (234 nm). Purity of synthesized compounds was checked by LC/MS analyses, which were realized at the Faculté de Pharmacie of Marseille with a Thermo Scientific Accela High Speed LC System^®®^ (Waltham, MA, USA) coupled using a single quadrupole mass spectrometer Thermo MSQ Plus^®®^. The RP-HPLC column is a Thermo Hypersil Gold^®®^ 50 × 2.1 mm (C18 bounded), with particles of a diameter of 1.9 mm. The volume of sample injected on the column was 1 μL. Chromatographic analysis, total duration of 8 min, was on the gradient of the following solvents: t = 0 min, methanol/water 50:50; 0 < t < 4 min, linear increase in the proportion of methanol to a methanol/water ratio of 95:5; 4 < t < 6 min, methanol/water 95:5; 6 < t < 7 min, linear decrease in the proportion of methanol to return to a methanol/water ratio of 50:50; 6 < t < 7 min, methanol/water 50:50. The water used was buffered with ammonium acetate 5 mM. The flow rate of the mobile phase was 0.3 mL/min. The retention times (t_R_) of the molecules analyzed were indicated in min. The microwave reactions were performed using multimode reactors: ETHOS Synth Lab station and MicroSYNTH^®®^ Lab terminal 1024 (Ethos start, MLS GmbH, Leutkirch, Germany); or monomode reactors: Biotage Initiator^®®^ classic in sealed vials with a power output of 0 to 400 W. Reagents were purchased and used without further purifications from Sigma-Aldrich or Fluorochem.

#### 3.1.2. General Procedure for 2-chloro-3-methyl Substituted Quinoxaline (2a–2p)

To a solution of 2-chloro-3-methylquinoxaline (1) (500 mg, 2.8 mmol) and the appropriate phenol (2.8 mmol, 1.0 equiv) in anhydrous DMF (10 mL), Cs_2_CO_3_ (912 mg, 2.8 mmol, 1.0 equiv) was added under inert atmosphere. The mixture was stirred at 70 °C overnight. After completion of the reaction, water was added, leading to a precipitate which was separated by filtration. The resulting precipitate was then thoroughly washed with water. The precipitate was dissolved in CH_2_Cl_2_ and dried with Na_2_SO_4_. After filtration and evaporation, the resulting solid was purified by silica gel column chromatography (using the appropriate eluant) to afford the desired compound.

##### 2-Methyl-3-phenoxyquinoxaline (2a)

Yield 79% (523 mg). White solid. Mp 90 °C. ^1^H NMR (250 MHz, CDCl_3_) δ 8.02–7.96 (m, 1H), 7.74–7.67 (m, 1H), 7.60–7.54 (m, 2H), 7.51–7.43 (m, 2H), 7.32–7.19 (m, 3H), 2.84 (s, 3H). ^13^C NMR (63 MHz, CDCl_3_) δ 156.2, 153.0, 148.2, 139.6, 139.2, 129.7 (2C), 129.3, 127.9, 127.5, 125.4, 121.8 (2C), 115.5, 20.7. LC-MS (ESI+) t_R_ 6.01 min, *m/z* [M + H]^+^ 237.27. MW: 236.27 g.mol^−1^. HRMS (ESI): *m/z* [M + H]^+^ calcd for C_15_H_12_N_2_O: 237.1022, Found: 237.1022.

##### 2-(2-Chlorophenoxy)-3-methylquinoxaline (2b)

Yield 73% (553 mg). Brown solid. Mp 140 °C. ^1^H NMR (250 MHz, CDCl_3_) δ 8.04–7.97 (m, 1H), 7.69–7.63 (m, 1H), 7.61–7.55 (m, 2H), 7.54–7.49 (m, 1H), 7.40–7.35 (m, 2H), 7.30–7.23 (m, 2H), 2.88 (s, 2H). ^13^C NMR (63 MHz, CDCl_3_) δ 155.5, 149.2, 147.7, 139.5, 139.4, 130.7, 129.3, 128.0, 127.9, 127.6, 127.5, 127.2, 126.7, 124.4, 20.7. LC-MS (ESI+) t_R_ 3.77 min, *m/z* [M + H]^+^ 271.15/273.19. MW: 270.71 g.mol^−1^. HRMS (ESI): *m/z* [M + H]^+^ calcd for C_15_H_10_ClN_2_O: 271.0633, Found: 271.0632.

##### 2-(3-Chlorophenoxy)-3-methylquinoxaline (2c)

Yield 90% (682 mg). Red-brown solid. Mp 108 °C. ^1^H NMR (250 MHz, CDCl_3_) δ 8.02–7.95 (m, 1H), 7.76–7.69 (m, 1H), 7.62–7.56 (m, 2H), 7.42–7.24 (m, 2H), 7.21–7.16 (m, 1H), 2.81 (s, 3H). ^13^C NMR (63 MHz, CDCl_3_) δ 155.7, 153.5, 147.9, 139.5, 139.3, 134.9, 130.4, 129.4, 128.0, 127.7, 127.5, 125.7, 122.4, 120.2, 20.6. LC-MS (ESI+) t_R_ 4.13 min, *m/z* [M + H]^+^ 271.09/273.18. MW: 270.71 g.mol^−1^. HRMS (ESI): *m/z* [M + H]^+^ calcd for C_15_H_10_ClN_2_O: 271.0633, Found: 271.0634.

##### 2-(2,4-Dichlorophenoxy)-3-methylquinoxaline (2d)

Yield 83% (709 mg). Yellow solid. Mp 110 °C. ^1^H NMR (250 MHz, CDCl_3_) δ 7.95–7.91 (m, 1H), 7.63–7.59 (m, 1H), 7.53–7.45 (m, 2H), 7.45 (d, J = 1.9 Hz, 1H), 7.32–7.20 (m, 2H), 2.82 (s, 3H). ^13^C NMR (63 MHz, CDCl_3_) δ 155.2, 147.9, 147.5, 139.7, 139.3, 131.4, 130.4, 129.4, 128.1, 128.1, 127.7, 127.4, 125.3, 20.6. (a quaternary carbon was visible in these conditions). LC-MS (ESI+) t_R_ 4.61 min, *m/z* [M + H]^+^ 304.92/306.97/309.10. MW: 305.16 g.mol^−1^. HRMS (ESI): *m/z* calcd. For C_15_H_10_Cl_2_N_2_O [M + H]^+^ 305.0243. Found: 305.0244.

##### 2-(2-Fluorophenoxy)-3-methylquinoxaline (2e)

Yield 53% (377 mg). Brown solid. Mp 149 °C. ^1^H NMR (250 MHz, CDCl_3_) δ 8.04–7.96 (m, 1H), 7.71–7.65 (m, 1H), 7.60–7.53 (m, 2H), 7.37–7.19 (m, 3H), 2.86 (s, 3H). ^13^C NMR (63 MHz, CDCl_3_) δ 156.7 (d, J = 250.2 Hz), 155.4, 152.7, 147.4, 140.4, 140.2, 139.5, 129.3, 128.0, 127.5 (d, J = 4.6 Hz), 126.8 (d, J = 7.2 Hz), 124.7(d, J = 3.8 Hz), 124.3, 117.0 (d, J = 18.4 Hz), 94.8. LC-MS:t_R_= 3.48 min; *m/z* [M + H]^+^ 255.19. MW: 254.26 g.mol^−1^. HRMS (ESI): *m/z* calcd. For C_15_H_10_FN_2_O [M + H]^+^ 255.0928. Found: 255.0928.

##### 2-(3-Fluorophenoxy)-3-methylquinoxaline (2f)

Yield 92% (655 mg). Brown solid. Mp 83 °C. ^1^H NMR (250 MHz, CDCl_3_) δ 8.01–7.95 (m, 1H), 7.75–7.69 (m, 1H), 7.62–7.55 (m, 2H), 7.47–7.36 (m, 1H), 7.09–6.95 (m, 2H), 2.81 (s, 3H). ^13^C NMR (63 MHz, CDCl_3_) δ 163.2 (d, J = 247.0 Hz), 155.7, 153.9 (d, J = 10.9 Hz), 147.9, 139.4, 139.4, 130.4 (d, J = 9.5 Hz), 129.4, 128.02, 127.7, 127.5, 117.5 (d, J = 3.3 Hz), 112.5 (d, J = 21.1 Hz), 109.8 (d, J = 24.4 Hz), 20.6. LC-MS (ESI+) t_R_ 3.69 min, *m/z* [M + H]^+^ 255.27. MW: 254.26 g.mol^−1^. HRMS (ESI): *m/z* calcd. For C_15_H_10_FN_2_O [M + H]^+^ 255.0928. Found: 255.0928.

##### 2-(4-Fluorophenoxy)-3-methylquinoxaline (2g)

Yield 91% (648 mg). Brown solid. Mp 86 °C. ^1^H NMR (250 MHz, CDCl_3_) δ 8.05–7.95 (m, 1H), 7.73–7.64 (m, 1H), 7.63–7.53 (m, 2H), 7.25–7.09 (m, 3H), 2.83 (s, 3H). ^13^C NMR (63 MHz, CDCl_3_) δ 160.1 (d, J = 243.6 Hz), 156.1, 148.6 (d, J = 2.9 Hz), 147.9, 139.3 (d, J = 21.0 Hz, 2C), 129.4, 127.9, 127.6, 127.4, 123.3 (d, J = 8.4 Hz, 2C), 116.5, 116.1, 20.6. LC-MS (ESI+) tR 3.63 min, *m/z* [M + H]^+^ 255.18. MW: 254.26 g.mol^−1^. HRMS (ESI): *m/z* calcd. For C_15_H_10_FN_2_O [M + H]+ 255.0928. Found: 255.0928.

##### 2-(4-Methoxyphenoxy)-3-methylquinoxaline (2h)

Yield 97% (723 mg). White solid. Mp 144 °C. ^1^H NMR (250 MHz, CDCl_3_) δ 7.98–7.95 (m, 1H), 7.72–7.68 (m, 1H), 7.57–7.53 (m, 2H), 7.19 (d, J = 9.0 Hz, 2H), 6.97 (d, J = 9.0 Hz, 2H), 3.85 (s, 3H), 2.81 (s, 3H). ^13^C NMR (63 MHz, CDCl_3_) δ 157.0, 156.5, 148.1, 146.3, 139.6, 139.2, 129.1, 128.0, 127.4, 127.2, 122.7(2C), 114.6(2C), 55.7, 20.7. LC-MS (ESI+) t_R_ 6.00 min; *m/z* [M + H]^+^ 267.22. MW: 266.29 g.mol^−1^. HRMS (ESI): *m/z* calcd. For C_16_H_14_N_2_O_2_ [M + H]^+^ 267.1128. Found: 267.1128.

##### 2-Methyl-3-(4-nitrophenoxy)quinoxaline (2i)

Yield 86% (677 mg). Brown powder. Mp 163 °C.^1^H NMR (250 MHz, CDCl_3_) δ 8.35–8.25 (m, 2H), 8.00–7.93 (m, 1H), 7.84–7.76 (m, 2H), 7.75–7.70 (m, 1H), 7.68–7.59 (m, 2H), 2.80 (s, 3H). ^13^C NMR (101 MHz, CDCl_3_) δ 153.5, 151.7, 148.0, 141.2, 140.4, 138.2, 134.9 (2C), 129.6, 129.3, 128.5, 128.1, 124.0 (2C), 22.5. LC-MS (ESI+) t_R_ 4.26 min, no ionization. MW: 281.27g.mol-1. HRMS (ESI): *m/z* calcd. For C_15_H_11_N_3_O_2_S [M + H]^+^ 282.0873. Found: 282.0869.

##### 2-Methyl-3-(4-trifluoromethylphenoxy)quinoxaline (2j)

Yield 90% (767 mg). Pink solid. Mp 95 °C. ^1^H NMR (250 MHz, CDCl_3_) δ 8.06–7.93 (m, 1H), 7.77–7.67 (m, 3H), 7.66–7.55 (m, 2H), 7.41 (d, J = 8.3 Hz, 2H), 2.83 (s, 3H). ^13^C NMR (63 MHz, CDCl_3_) δ 155.6 (q, J = 1.4 Hz), 155.5, 147.9, 139.8, 139.2, 129.5, 128.2, 127.8, 127.5 (q, J = 32.8 Hz), 127.4, 127.0 (q, J = 3.7 Hz), 124.2 (q, J = 271.9 Hz), 122.1, 20.7. LC-MS (ESI+) t_R_ 4.99 min, *m/z* [M + H]+ 304.44. MW: 304.28 g.mol^−1^. HRMS (ESI): *m/z* calcd. For C_16_H_11_F_3_N_2_O [M + H]^+^ 305.0896. Found: 305.0894.

##### 2-Methyl-3-(4-trifluoromethoxyphenoxy)quinoxaline (2k)

Yield 86% (771 mg). Beige solid. Mp 86 °C. ^1^H NMR (250 MHz, CDCl_3_) δ 8.01–7.77 (m, 2H), 7.72–7.59 (m, 2H), 7.59–7.45 (m, 4H), 2.77 (s, 3H). ^13^C NMR (101 MHz, CDCl_3_) δ 155.8, 151.2, 147.9, 146.3 (q, J = 1.9 Hz), 139.6, 139.4, 129.4, 128.1, 127.7, 127.4, 123.1 (2C), 122.3, 120.7 (q, J = 257.1 Hz), 20.7. LC-MS (ESI+) t_R_ 4.70 min, *m/z* [M + H]^+^ 321.22. MW: 320.27 g.mol-1. HRMS (ESI): *m/z* calcd. For C_16_H_11_F_3_N_2_O_2_ [M + H]+ 321.0845. Found: 321.0842.

##### 2-Methyl-3-[4-(pentafluorosulfanyl)phenoxy]quinoxaline (2l)

Yield 90% (914 mg). Beige solid. Mp 154 °C.^1^H NMR (250 MHz, CDCl_3_) δ 8.07–7.94 (m, 1H), 7.93–7.77 (m, 2H), 7.78–7.66 (m, 1H), 7.67–7.55 (m, 2H), 7.46–7.33 (m, 2H), 2.83 (s, 3H). ^13^C NMR (101 MHz, CDCl_3_) δ 155.2, 155.0, 150.5 (t, J = 17.9 Hz), 147.8, 139.8, 139.2, 129.6, 128.2, 128.0, 127.8 (p, J = 4.6 Hz), 127.4, 121.8, 20.6. LC-MS (ESI+) t_R_ 4.58 min, *m/z* [M + H]^+^ 362.90. MW: 362.32 g.mol^−1^. HRMS (ESI): *m/z* calcd. For C_15_H_11_F_5_N_2_OS [M + H]^+^ 363.0585. Found: 363.0584.

##### 2-Methyl-3-[4-(trifluoromethylthio)phenoxy]quinoxaline (2m)

Yield 87% (819 mg). Pink powder. Mp 85 °C. ^1^H NMR (250 MHz, CDCl_3_) δ 8.04–7.95 (m, 1H), 7.79–7.68 (m, 3H), 7.65–7.56 (m, 2H), 7.40–7.33 (m, 2H), 2.82 (s, 3H). ^13^C NMR (101 MHz, CDCl_3_) δ 155.4, 148.0, 139.8, 139.3, 138.1 (2C), 129.7 (q, J = 308.2 Hz), 129.5, 128.2, 127.9, 127.5, 122.8 (2C), 120.6, 120.6 (q, J = 2.2 Hz), 20.7. LC-MS (ESI+) t_R_ 4.93 min, *m/z* [M + H]^+^ 336.61. MW: 336.33 g.mol-1. HRMS (ESI): *m/z* calcd. For C_16_H_11_F_3_N_2_OS [M + H]^+^ 337.0617. Found: 337.0615.

##### 4-[(3-Methylquinoxalin-2-yl)oxy]benzonitrile (2n)

Yield 72% (527 mg). Pink powder. Mp 188 °C. ^1^H NMR (250 MHz, CDCl_3_) δ 8.04–7.96 (m, 1H), 7.81–7.74 (m, 2H), 7.72–7.66 (m, 1H), 7.66–7.59 (m, 2H), 7.46–7.39 (m, 2H), 2.82 (s, 3H). ^13^C NMR (63 MHz, CDCl_3_) δ 156.5, 155.1, 147.8, 139.9, 139.1, 134.0 (2C), 129.6, 128.3, 128.1, 127.4, 122.7 (2C), 118.7, 109.1, 20.7. LC-MS (ESI+) t_R_ 3.01 min, *m/z* [M + H]^+^ 262.03. MW: 261.28 g.mol^−1^. HRMS (ESI): *m/z* calcd. For C_16_H_11_N_3_O [M + H]^+^ 262.0975. Found: 262.0975.

##### 2-Methyl-3-[4-(trifluoromethylsulfonyl)phenoxy]quinoxaline (2o)

Yield 45% (464 mg). White solid. Mp 156 °C. ^1^H NMR (400 MHz, CDCl_3_) δ 8.14 (d, J = 8.8 Hz, 2H), 8.06–7.99 (m, 1H), 7.79–7.72 (m, 1H), 7.68–7.61 (m, 4H), 2.84 (s, 3H). ^13^C NMR (101 MHz, CDCl_3_) δ 159.9, 154.6, 147.7, 140.2, 138.9, 133.0, 129.9, 128.5, 128.4, 127.5, 127.1, 122.8, 119.9 (d, J = 325.7 Hz), 20.7. LC-MS (ESI+) t_R_ 4.02 min, *m/z* [M + H]^+^ 368.51. MW: 368,33 g.mol^−1^. HRMS (ESI): *m/z* calcd. For C_16_H_11_F_3_N_2_O_3_S [M + H]^+^ 369.0515. Found: 369.0513.

##### 2-Methyl-3-(naphthalen-1-yloxy)quinoxaline (2q)

Yield 94% (754 mg). Beige solid. Mp 169 °C. ^1^H NMR (400 MHz, CDCl_3_) δ 8.04–7.97 (m, 1H), 7.96–7.90 (m, 2H), 7.81 (d, J = 8.2 Hz, 1H), 7.62–7.50 (m, 5H), 7.49–7.41 (m, 2H), 2.98 (s, 3H). ^13^C NMR (101 MHz, CDCl_3_) δ 156.5, 149.0, 147.9, 139.7, 139.4, 135.1, 129.2, 128.2, 128.0, 127.6, 127.5, 127.3, 126.5, 126.4, 125.7, 125.6, 121.8, 118.0, 20.9. LC-MS (ESI+) t_R_ 4.24 min, *m/z* [M + H]^+^ 287.05. MW: 286.33 g.mol^−1^. HRMS (ESI): *m/z* calcd. For C_19_H_14_N_2_O [M + H]^+^ 287.1179. Found: 287.1180.

##### 2-Methyl-3-(naphthalen-2-yloxy)quinoxaline (2r)

Yield 90% (721 mg). Beige solid. Mp 149 °C. ^1^H NMR (250 MHz, CDCl_3_) δ 7.97–7.89 (m, 1H), 7.89–7.79 (m, 2H), 7.78–7.72 (m, 1H), 7.64 (t, J = 3.3 Hz, 1H), 7.59 (m, 1H), 7.54–7.46 (m, 2H), 7.46–7.39 (m, 2H), 7.37–7.29 (m, 1H), 2.89–2.72 (m, 3H). ^13^C NMR (101 MHz, CDCl_3_) δ 156.5, 149.0, 147.9, 139.7, 139.5, 135.1, 129.2, 128.2, 128.0, 127.6, 127.5, 127.3, 126.5, 126.4, 125.7, 125.6, 121.8, 118.0, 20.9. LC-MS (ESI+) t_R_ 4.45 min, *m/z* [M + H]^+^ 287.02. MW: 286.33 g.mol^−1^. HRMS (ESI): *m/z* calcd. For C_19_H_14_N_2_O [M + H]^+^ 287.1179. Found: 287.1180.

##### 2-([1,1′-Biphenyl]-4-yloxy)-3-methylquinoxaline (2s)

Yield 99% (866 mg). White solid. Mp 158 °C. ^1^H NMR (250 MHz, CDCl_3_) δ 8.03–7.94 (m, 1H), 7.78–7.68 (m, 2H), 7.68–7.61 (m, 3H), 7.60–7.55 (m, 2H), 7.52–7.42 (m, 2H), 7.41–7.31 (m, 3H), 2.85 (s, 3H). ^13^C NMR (63 MHz, CDCl_3_) δ 156.1, 152.6, 148.2, 140.7, 139.6 (2C), 138.5, 129.2, 129.0 (2C), 128.4 (2C), 128.2, 127.5, 127.4 (2C), 127.3 (2C), 122.0 (2C), 20.8. LC-MS (ESI+) t_R_ 4.81 min, *m/z* [M + H]^+^ 313.08. MW: 312.36 g.mol^−1^. HRMS (ESI): *m/z* calcd. For C_21_H_16_N_2_O [M + H]^+^ 313.1335. Found: 313.1331.

##### 2-([1,1′-Biphenyl]-3-yloxy)-3-methylquinoxaline (2t)

Yield 99% (866 mg). White solid. Mp 109 °C. ^1^H NMR (250 MHz, CDCl_3_) δ 8.05–7.95 (m, 1H), 7.79–7.69 (m, 1H), 7.67–7.55 (m, 4H), 7.55–7.50 (m, 3H), 7.49–7.41 (m, 2H), 7.40–7.34 (m, 1H), 7.31–7.25 (m, 1H), 2.85 (s, 3H). ^13^C NMR (63 MHz, CDCl_3_) δ 156.1, 153.5, 148.2, 143.1, 140.5, 139.6 (2C), 129.9, 129.2, 129.0 (2C), 128.2, 127.8, 127.5, 127.4, 127.3 (2C), 124.1, 120.5 (2C), 20.8. LC-MS (ESI+) t_R_ 4.82 min, *m/z* [M + H]^+^ 313.09. MW: 312.36 g.mol^−1^. HRMS (ESI): *m/z* calcd. For C_21_H_16_N_2_O [M + H]^+^ 313.1335. Found: 313.1332.

#### 3.1.3. General Procedure for Preparation of 3-thiophenoxy-2-trichloromethylquinoxaline Derivatives

To a solution of 2-chloro-3-methyl substituted quinoxaline (2a–2p) (624 mg, 2.8 mmol) and PCl_5_ (2.88 g, 16.8 mmol), POCl_3_ was added to make a slurry (ca 5 mL). The mixture was then heated in a multimode microwave oven at 100 °C, 800 W for 20–30 min. After completion of the reaction, the mixture was poured into ice and was then neutralized with Na_2_CO_3_. The resulting solution was extracted with CH_2_Cl_2_ and dried with Na_2_SO_4_. After filtration and evaporation, the resulting solid was purified by silica gel column chromatography (eluent: Petroleum Ether/CH_2_Cl_2_, 9:1) to afford the desired compound.

##### 2-Phenoxy-3-trichloromethylquinoxaline (3a)

Yield 84% (799 mg). White solid. Mp 59 °C. ^1^H NMR (250 MHz, CDCl_3_) δ 8.14–8.06 (m, 1H), 7.72–7.56 (m, 3H), 7.43–7.37 (m, 2H), 7.26–7.16 (m, 3H). ^13^C NMR (63 MHz, CDCl_3_) δ 152.8, 152.7, 142.5, 141.3, 136.9, 132.2, 129.8, 129.8 (2C), 128.5, 127.1, 125.8, 121.8 (2C), 95.0. LC-MS (ESI+) t_R_ 5.0 min, *m/z* [M + H]^+^ no ionization. MW: 339.6 g.mol^−1^. HRMS (ESI): *m/z* [M + H]^+^ calcd for: C_15_H_9_Cl_3_N_2_O: 338.9853. Found: 338.9854.

##### 2-(2-Chlorophenoxy)-3-trichloromethylquinoxaline (3b)

Yield 67% (702 mg). White solid. Mp 99 °C. ^1^H NMR (250 MHz, CDCl_3_) δ 8.19–8.15 (m, 1H), 7.77–7.66 (m, 3H), 7.58–7.52 (m, 1H), 7.42–7.36 (m, 1H), 7.32–7.27 (m, 2H). ^13^C NMR (63 MHz, CDCl_3_) δ 151.9, 148.8, 141.9, 141.3, 137.1, 132.2, 130.8, 129.9, 128.6, 128.1, 127.6, 127.2, 127.1, 124.0, 94.8. LC-MS (ESI+) t_R_ 5.11 min, *m/z* [M + H]^+^ no ionization. MW: 374.05 g.mol^−1^. HRMS (ESI): *m/z* calcd. for C_15_H_8_Cl_4_N_2_O [M + H]^+^ 372.9464. Found: 372.9464.

##### 2-(3-Chlorophenoxy)-3-(richloromethylquinoxaline (3c)

Yield 67% (702 mg). White solid. Mp 58 °C. ^1^H NMR (250 MHz, CDCl_3_) δ 8.08–8.04 (m, 1H), 7.72–7.56 (m, 3H), 7.32–7.10 (m, 4H). ^13^C NMR (63 MHz, CDCl_3_) δ 153.1, 152.3, 142.3, 141.1, 137.1, 135.0, 132.4, 130.5, 129.9, 128.9, 127.1, 126.1, 122.4, 120.1, 94.8. LC-MS (ESI+) t_R_ 5.74 min, *m/z* [M + H]^+^ no ionization. MW: 374.05 g.mol^−1^. HRMS (ESI): *m/z* calcd. for C_15_H_8_Cl_4_N_2_O [M + H]^+^ 372.9464. Found: 372.9463.

##### 2-(2,4-Dichlorophenoxy)-3-trichloromethylquinoxaline (3d)

Yield 52% (595 mg). White solid. Mp 120 °C. ^1^H NMR (250 MHz, CDCl_3_) δ 8.22–8.13 (m, 1H), 7.78–7.66 (m, 3H), 7.56 (d, J = 2.4 Hz, 1H), 7.36 (dd, J = 8.7 Hz, 2.4 Hz, 1H), 7.26–7.21 (m, 1H). ^13^C NMR (63 MHz, CDCl_3_) δ 151.7, 147.6, 141.8, 141.1, 137.2, 132.4, 132.0, 130.6, 129.9, 128.9, 128.6, 128.3, 127.1, 124.9, 94.7. LC-MS (ESI+) t_R_ 5.5 min, *m/z* [M + H]^+^ no ionization. MW: 408.49 g.mol^−1^. HRMS (ESI): *m/z* calcd. for C_15_H_7_Cl_5_N_2_O [M + H]^+^ 406.9074. Found: 406.9074.

##### 2-(2-Fluorophenoxy)-3-trichloromethylquinoxaline (3e)

Yield 43% (430 mg). White solid. Mp 138 °C. ^1^H NMR (250 MHz, CDCl_3_) δ 8.19–8.15 (m, 1H), 7.78–7.66 (m, 3H), 7.40–7.21 (m, 4H). ^13^C NMR (63 MHz, CDCl_3_) δ 154.6 (d, *J* = 250.2 Hz), 151.9, 141.9, 141.3, 139.9, 139.7, 137.2, 132.3, 129.9, 128.7, 127.2 (d, *J* = 7.0 Hz), 124.8 (d, *J* = 3.9 Hz), 124.1, 117.1 (d, *J* = 18.3 Hz), 94.8. LC-MS (ESI+) t_R_ 4.9 min, *m/z* [M + H]^+^ no ionization. MW: 357.59 g.mol^−1^. HRMS (ESI): *m/z* calcd. for C_15_H_8_Cl_3_FN_2_O [M + H]^+^ 356.9759. Found: 356.9762.

##### 2-(3-Fluorophenoxy)-3-trichloromethylquinoxaline (3f)

Yield 52% (521 mg). White solid. Mp 101 °C. ^1^H NMR (250 MHz, CDCl_3_) δ 8.22–8.13 (m, 1H), 7.84–7.68 (m, 3H), 7.49–7.39 (m, 1H), 7.16–7.00 (m, 3H). ^13^C NMR (63 MHz, CDCl_3_) δ 163.2 (d, J = 247.5 Hz), 153.5 (d, J = 11.0 Hz), 152.3, 142.4, 141.2, 137.1, 132.4, 130.5 (d, J = 9.4 Hz), 129.9, 128.9, 127.1, 117.5 (d, J = 3.3 Hz), 112.8 (d, J = 21.0 Hz), 109.9 (d, J = 24.5 Hz), 94.8. LC-MS (ESI+) t_R_ 5.1 min, *m/z* [M + H]^+^ no ionization. MW: 357.59 g.mol^−1^. HRMS (ESI): *m/z* calcd. for C_15_H_8_Cl_3_FN_2_O [M + H]^+^ 356.9759. Found: 356.9761.

##### 2-(4-Fluorophenoxy)-3-trichloromethylquinoxaline (3g)

Yield 58% (581 mg). White solid. Mp 56 °C. ^1^H NMR (250 MHz, CDCl_3_) δ 8.17–8.13 (m, 1H), 7.79–7.63 (m, 3H), 7.33–7.24 (m, 2H), 7.20–7.10 (m, 2H). ^13^C NMR (63 MHz, CDCl_3_) δ 160.3 (d, J = 244.2 Hz), 152.7, 148.4 (d, J = 2.9 Hz), 142.3, 141.2, 136.9, 132.3, 129.9, 128.6, 127.0, 123.3 (d, J = 8.5 Hz, 2C), 116.4 (d, J = 23.5 Hz, 2C), 94.9. LC-MS (ESI+) t_R_ 4.9 min, *m/z* [M + H]+ no ionization. MW: 357.59 g.mol^−1^. HRMS (ESI): *m/z* calcd. for C_15_H_8_Cl_3_FN_2_O [M + H]^+^ 356.9759. Found: 356.9754.

##### 2-(4-Methoxyphenoxy)-3-trichloromethylquinoxaline (3h)

Yield 27% (270 mg). White solid. Mp 171 °C. ^1^H NMR (250 MHz, CDCl_3_) δ 8.17–8.13 (m, 1H), 7.81–7.64 (m, 3H), 7.26–7.23 (m, 2H), 7.01–6.97 (m, 2H), 3.86 (s, 3H). ^13^C NMR (63 MHz, CDCl_3_) δ 157.3, 153.1, 146.1, 142.4, 141.4, 136.9, 132.1, 129.8, 128.4, 127.1, 122.6 (2C), 114.7 (2C), 95.0, 55.8. LC-MS (ESI+) t_R_ 4.9 min, *m/z* [M + H]^+^ no ionization. MW: 357.59 g.mol^−1^. HRMS (ESI): *m/z* calcd. for C_16_H_11_Cl_3_N_2_O_2_ [M + H]^+^ 368.9959. Found: 368.9957.

##### 2-(4-Nitrophenoxy)-3-trichloromethylquinoxaline (3i)

Yield 57% (614 mg). Yellow solid. Mp 178 °C. ^1^H NMR (250 MHz, CDCl_3_) δ 8.41–8.35 (m, 2H), 8.23–8.19 (m, 1H), 7.82–7.73 (m, 3H), 7.54–7.48 (m, 2H). ^13^C NMR (63 MHz, CDCl_3_) δ 157.6, 151.7, 145.2, 142.4, 140.9, 137.4, 132.7, 130.0, 129.4, 127.1, 125.7 (2C), 122.3 (2C), 94.6. LC-MS (ESI+) t_R_ 4.12 min; *m/z* [M + H]^+^ no ionization. MW: 384.60 g.mol^−1^. HRMS (ESI): *m/z* calcd. for: C_15_H_8_Cl_3_N_3_O_3_ [M + H]^+^ 383.9704. Found: 383.9704.

##### 2-Trichloromethyl-3-[4-(trifluoromethyl)phenoxy]quinoxaline (3j)

Yield 70% (799 mg). White powder. Mp 129 °C. ^1^H NMR (250 MHz, CDCl_3_) δ 8.30–8.08 (m, 1H), 7.93–7.62 (m, 5H), 7.60–7.36 (m, 2H). ^13^C NMR (63 MHz, CDCl_3_) δ 155.3, 152.1, 142.5, 141.1, 137.2, 132.5, 130.0, 129.0, 127.9 (q, J = 32.8 Hz), 127.2 (q, J = 3.7 Hz), 127.1 (2C), 124.1 (q, J = 272.0 Hz), 122.1 (2C), 94.8. LC-MS (ESI+) t_R_ 6.62 min, *m/z* [M + H]^+^ don’t ionise. MW: 407.60 g.mol^−1^. HRMS (ESI): *m/z* calcd. for C_16_H_8_Cl_3_F_3_N_2_O: 406.9727, Found: 406.9722.

##### 2-Trichloromethyl-3-[4-(trifluoromethoxy)phenoxy]quinoxaline (3k)

Yield 85% (1008 mg). White solid. Mp 66 °C. ^1^H NMR (250 MHz, CDCl_3_) δ 8.22–8.14 (m, 1H), 7.84–7.69 (m, 3H), 7.44–7.30 (m, 4H). ^13^C NMR (63 MHz, CDCl_3_) δ 152.4, 150.9, 146.6 (q, J = 1.9 Hz), 142.4, 141.1, 137.1, 132.4, 129.9, 128.9, 127.1, 123.1, 122.5, 120.6 (q, J = 257.3 Hz), 94.9. LC-MS (ESI+) t_R_ 5.72 min, *m/z* [M + H]^+^ Don’t ionise. MW: 423.60 g.mol^−1^. HRMS (ESI): *m/z* calcd. for C_16_H_8_Cl_3_F_3_N_2_O_2_: 422.9676, Found: 422.9675.

##### 2-Trichloromethyl-3-[4-(pentafluorosulfanyl)phenoxy]quinoxaline (3l)

Yield 84% (1095 mg). Green crystal. Mp 99 °C. ^1^H NMR (250 MHz, CDCl_3_) δ 8.29–8.13 (m, 1H), 7.93–7.71 (m, 5H), 7.44 (d, J = 8.8 Hz, 2H). ^13^C NMR (63 MHz, CDCl_3_) δ 154.6, 151.9, 150.8 (p, J = 17.2 Hz), 142.4, 141.0, 137.3, 132.6 (2C), 130.0, 129.2, 127.9 (p, J = 4.6 Hz), 127.1, 121.7 (2C), 94.7. LC-MS (ESI+) t_R_ 5.70 min, *m/z* [M + H]^+^ 468.77. MW: 465.65 g.mol^−1^. HRMS (ESI): *m/z* calcd. for C_15_H_8_Cl_3_F_5_N_2_OS: 466.9387, Found: 466.9315.

##### 2-Trichloromethyl-3-[4-(trifluoromethylthio)phenoxy]quinoxaline (3m)

Yield 58% (714 mg). White crystal. Mp 94 °C. ^1^H NMR (250 MHz, CDCl_3_) δ 8.24–8.14 (m, 1H), 7.86–7.69 (m, 5H), 7.46–7.38 (m, 2H). ^13^C NMR (63 MHz, CDCl_3_) δ 155.0, 152.1, 142.5, 141.1, 138.2, 137.2, 132.5, 130.0, 129.6 (q, J = 307.7 Hz), 129.0, 127.1, 122.8, 121.2 (q, J = 2.1 Hz), 94.8. LC-MS (ESI+) t_R_ 5.8 min, *m/z* [M + H]^+^ no ionization. MW: 439.67 g.mol^−1^. HRMS (ESI): *m/z* calcd. for C_16_H_8_Cl_3_F_3_N_2_OS: 440.9419, Found: 440.9419.

##### 4-[(3-Trichloromethylquinoxalin-2-yl)oxy]benzonitrile (3n)

Yield 81% (827 mg). White solid. Mp 194 °C. ^1^H NMR (250 MHz, CDCl_3_) δ 8.23–8.14 (m, 1H), 7.86–7.71 (m, 5H), 7.51–7.42 (m, 2H). ^13^C NMR (63 MHz, CDCl_3_) δ 156.1, 151.8, 142.4, 140.9, 137.3, 134.1 (2C), 132.6, 130.0, 129.3, 127.1, 122.6 (2C), 118.5, 109.5, 94.6. LC-MS (ESI+) t_R_ 4.62 min, *m/z* [M + H]^+^ no ionization. MW: 364.61 g.mol^−1^. HRMS (ESI): *m/z* calcd. for C_16_H_8_Cl_3_N_3_O: 363.9806, Found: 36.9802.

##### 2-Trichloromethyl-3-[4-(trifluoromethylsulfonyl)phenoxy]quinoxaline (3o)

Yield 26% (343 mg). White solid. Mp 107 °C. ^1^H NMR (400 MHz, CDCl_3_) δ 8.25–8.20 (m, 1H), 8.19–8.13 (m, 2H), 7.88–7.83 (m, 2H), 7.82–7.76 (m, 1H), 7.69–7.62 (m, 2H). ^13^C NMR (101 MHz, CDCl_3_) δ 159.4, 151.3, 142.6, 140.8, 137.6, 133.1, 132.9, 130.1, 129.7, 127.8, 127.2, 122.8, 120.0 (q, J = 325.6 Hz), 94.5. LC-MS (ESI+) t_R_ 5.08 min, *m/z* [M + H]^+^ no ionization. MW: 471.67 g.mol^−1^. HRMS (ESI): *m/z* calcd. for C_16_H_8_Cl_3_F_3_N_2_O_3_S: 472.9318, Found: 472.9319.

##### 2-(Naphthalen-2-yloxy)-3-trichloromethylquinoxaline (3q)

Yield 64% (698 mg). White solid. Mp 143 °C, (isopropanol). ^1^H NMR (250 MHz, CDCl_3_) δ 8.24–8.13 (m, 1H), 8.00–7.83 (m, 3H), 7.83–7.78 (m, 1H), 7.78–7.65 (m, 3H), 7.57–7.49 (m, 2H), 7.49–7.41 (m, 1H). ^13^C NMR (63 MHz, CDCl_3_) δ 153.0, 150.5, 142.6, 141.4, 137.1, 134.2, 132.2, 131.6, 129.9, 129.7, 128.6, 128.0, 127.8, 127.1, 126.8, 125.8, 121.5, 118.5, 95.1. LC-MS (ESI+) t_R_ 5.66 min, *m/z* [M + H]^+^ 390.89/392.31/393.07. MW: 389.66 g.mol^−1^. HRMS (ESI): *m/z* calcd for C_19_H_11_Cl_3_N_2_O: 389.0010, Found: 389.0004.

##### 2-(Naphthalen-1-yloxy)-3-trichloromethylquinoxaline (3r)

Yield 26% (284 mg). Yellow solid. Mp 124 °C, (isopropanol). ^1^H NMR (250 MHz, CDCl_3_) δ 8.23–8.12 (m, 1H), 8.11–8.00 (m, 1H), 7.97–7.85 (m, 1H), 7.85–7.74 (m, 1H), 7.68–7.57 (m, 3H), 7.57–7.42 (m, 3H), 7.41–7.31 (m, 1H). ^13^C NMR (63 MHz, CDCl_3_) δ 153.3, 149.0, 142.3, 141.6, 137.1, 135.1, 132.2, 129.9, 128.6, 128.2, 127.5, 127.2, 126.7, 126.6, 126.0, 125.7, 122.2, 117.6, 95.2. LC-MS (ESI+) t_R_ 5.41 min, *m/z* [M + H]^+^ 388.93/390.95/392.94. MW: 389.66 g.mol^−1^. HRMS (ESI): *m/z* calcd for C_19_H_11_Cl_3_N_2_O: 389.0010, Found: 389.0004.

##### 2-([1,1′-Biphenyl]-4-yloxy)-3-trichloromethylquinoxaline (3s)

Yield 50% (582 mg). White solid. Mp 160 °C, (isopropanol). ^1^H NMR (250 MHz, CDCl_3_) δ 8.22–8.14 (m, 1H), 7.86–7.78 (m, 1H), 7.78–7.72 (m, 1H), 7.72–7.67 (m, 2H), 7.67–7.61 (m, 2H), 7.52–7.46 (m, 2H), 7.46–7.33 (m, 4H). ^13^C NMR (63 MHz, CDCl_3_) δ 152.8, 152.2, 142.6, 141.4, 140.6, 138.9, 137.1, 132.2, 129.9, 129.0 (2C), 128.6, 128.5 (2C), 127.5, 127.3 (2C), 127.2, 121.9 (2C), 95.0. LC-MS (ESI+) t_R_ 5.88 min, *m/z* [M + H]^+^ no ionization. MW: 415.70 g.mol^−1^. HRMS (ESI): *m/z* calcd for C_21_H_13_Cl_3_N_2_O: 415.0166, Found: 415.0164.

##### 2-([1,1′-Biphenyl]-3-yloxy)-3-trichloromethylquinoxaline (3t)

Yield 70% (815 mg). White oil. ^1^H NMR (250 MHz, CDCl_3_) δ 8.21–8.00 (m, 1H), 7.85–7.77 (m, 1H), 7.76–7.68 (m, 2H), 7.66–7.61 (m, 2H), 7.58–7.52 (m, 3H), 7.49–7.41 (m, 2H), 7.38 (m, 1H), 7.35–7.28 (m, 1H). ^13^C NMR (63 MHz, CDCl_3_) δ 153.2, 152.8, 143.3, 142.6, 141.4, 140.4, 137.1, 132.2, 130.0, 129.9, 129.0 (2C), 128.6, 127.9, 127.4, 127.3 (2C), 124.5, 120.5, 120.4, 95.1. LC-MS (ESI+) t_R_ 5.89 min, *m/z* [M + H]^+^ no ionization. MW: 415.70 g.mol^−1^. HRMS (ESI): *m/z* calcd for C_21_H_13_Cl_3_N_2_O: 415.0166, Found: 415.0164.

##### 2-Trichloromethyl-3-[4-(S-trifluoromethylsulfonimidoyl)phenoxy]quinoxaline (3p)

2-trichloromethyl-3-[4-(trifluoromethylthio)phenoxy]quinoxaline 3m (1.23 g, 2.8 mmol, 1 equiv.), trifluoroethanol (TFE, 0.4 M), ammonium carbamate (328 mg, 4.2 mmol, 1.5 equiv.), and phenyliodine (III) diacetate (PIDA) (1.9 g, 5.88 mmol, 2.1 equiv.) were successively added to a round bottom flask in one portion. The reaction mixture was stirred at room temperature for 3 h. After completion, trifluoroethanol was removed under reduced pressure. The crude mixture was diluted in an aqueous solution of HCl (6 M, 1 mL/mmol) and CH_3_CN (2 mL/mmol), and the reaction was stirred overnight at room temperature. The pH of the aqueous phase was adjusted to 7 with NaHCO_3_ (10% aqueous solution); then, the crude mixture was extracted with CH_2_Cl_2_ (3 × 10 mL). The organic layer was dried with MgSO_4_, concentrated under reduced pressure, and purified by column chromatography on silica gel.

Yield 28% (369 mg). White solid. Mp 127 °C. ^1^H NMR (400 MHz, CDCl_3_) δ 8.26 (d, J = 8.8 Hz, 2H), 8.23–8.17 (m, 1H), 7.86–7.81 (m, 2H), 7.78 (m, 1H), 7.65–7.58 (m, 2H), 3.65 (br, 1H). ^13^C NMR (101 MHz, CDCl_3_) δ 158.6, 151.6, 142.5, 140.9, 137.5, 132.9 (2C), 132.8, 130.1, 129.5, 128.1, 127.2, 122.5 (2C), 121.1 (q, J = 332.1 Hz), 94.6. LC-MS (ESI+) t_R_ 4.74 min, *m/z* [M + H]^+^ 469.61/471.50. MW: 470.68 g.mol^−1^. HRMS (ESI): *m/z* calcd. for C_16_H_9_Cl_3_F_3_N_3_O_2_S: 471.9478, Found: 471.9477.

#### 3.1.4. General Procedure for Compounds 3u–3v

##### Preparation of 2-chloro-3-trifluoromethylquinoxaline

Step 1: To a solution of *o*-phenylenediamine (2 g, 18.5 mmol, 1.0 equiv) in H_2_O, ethyl trifluoropyruvate was added (3g 146, 18.5 mmol, 1.0 equiv) and dissolved in H_2_O (30 mL). The reaction mixture was heated at 50 °C for 15 min. After cooling, the precipitate was filtered off and washed with H_2_O. 3-Trifluoromethylquinoxalin-2(1*H*)-one was recrystallized from ethanol, precipitating as a white solid, and engaged directly in step 2. Yield 93%.

Step 2: 3-Trifluoromethylquinoxalin-2-ol (3.5 g, 16.8 mmol, 1.0 equiv) was heated to reflux in phosphorus oxychloride (30 mL) for 2 h. After the starting material was consumed, the reaction mixture was cooled to r.t. and then quenched with ice at 0 °C. The precipitate was purified by flash chromatography to afford 2-chloro-3-trifluoromethylquinoxaline as a white solid. Yield 96%. Mp 140 °C. ^1^H NMR (400 MHz, CDCl_3_) δ 8.25–8.20 (m, 1H), 8.15–8.09 (m, 1H), 7.99–7.86 (m, 2H). NMR was consistent with the description provided in [36].

##### Preparation of compounds (3u, 3v)

To a solution of 2-chloroquinoxaline or 2-chloro-3-trifluoromethylquinoxaline [37] (300 mg, 1.3 mmol, 1.0 equiv.) and thiophenol reagent (143 mg, 1.3 mmol, 1.0 equiv.) in anhydrous DMF (5 mL), Cs_2_CO_3_ (1.0 equiv.) was added under inert atmosphere. The mixture was stirred at 70 °C overnight. After completion of the reaction, water was added, leading to a precipitate which was separated by filtration. The resulting precipitate was then thoroughly washed with water. The resulting solid was purified by silica gel column chromatography (eluent: Cyclohexane/CH_2_Cl_2_, 4:6) to afford the desired compound.

##### 2-(4-Nitrophenoxy)quinoxaline (3u)

Yield 73% (254 mg). Red solid. Mp 156 °C, (isopropanol). ^1^H NMR (400 MHz, CDCl_3_) δ 8.76 (s, 1H), 8.41–8.29 (m, 2H), 8.15–8.05 (m, 1H), 7.83–7.76 (m, 1H), 7.76–7.62 (m, 2H), 7.54–7.45 (m, 2H). ^13^C NMR (101 MHz, CDCl_3_) δ 157.9, 155.9, 144.9, 140.3, 139.6, 139.0, 131.0, 129.3, 128.4, 127.8, 125.7 (2C), 121.9 (2C). LC-MS (ESI+) t_R_ 3.14 min, *m/z* [M + H]^+^ 267.84. MW: 267,24 g.mol^−1^. HRMS (ESI): *m/z* calcd. for C_14_H_9_N_3_O_3_: 268.0717, Found: 268.0716.

##### 2-(4-Nitrophenoxy)-3-trifluoromethylquinoxaline (3v)

Yield 72% (314 mg). White solid. Mp 125 °C, (isopropanol). ^1^H NMR (400 MHz, CDCl_3_) δ 8.44–8.30 (m, 2H), 8.21 (d, J = 8.3 Hz, 1H), 7.90–7.68 (m, 3H), 7.57–7.40 (m, 2H). ^13^C NMR (101 MHz, CDCl_3_) δ 157.2, 152.9, 145.4, 141.1, 138.2, 134.8 (q, J = 36.8 Hz), 133.3, 130.0, 129.6, 127.6, 125.7 (2C), 122.5 (2C), 120.6 (q, J = 275.3 Hz). LC-MS (ESI+) t_R_ 4.14 min, *m/z* [M + H]^+^ No ionization. MW: 335,24 g.mol^−1^. HRMS (ESI): *m/z* calcd. for C_15_H_8_F_3_N_3_O_3_: 336.059, Found: 336.0585.

##### 2-Dichloromethyl-3-(4-nitrophenoxy)quinoxaline (3w)

Yield 20% (91 mg). White solid. Mp 112 °C, (isopropanol). ^1^H NMR (400 MHz, CDCl_3_) δ 8.43–8.32 (m, 2H), 8.20–8.12 (m, 1H), 7.80–7.76 (m, 2H), 7.76–7.70 (m, 1H), 7.55–7.47 (m, 2H), 7.24 (s, 1H). ^13^C NMR (101 MHz, CDCl_3_) δ 157.5, 152.4, 145.4, 143.4, 140.5, 139.0, 132.2, 129.6, 129.2, 127.5, 125.7 (2C), 122.5 (2C), 67.2. LC-MS (ESI+) t_R_ 4.1 min, *m/z* [M + H]^+^ No ionization. MW: 350.16 g.mol^−1^. HRMS (ESI): *m/z* calcd. for C_15_H_9_Cl_2_N_3_O_3_: 350.0094, Found: 350.0092.

### 3.2. Biology

#### 3.2.1. In Vitro Cytotoxicity Evaluation HepG2

The HepG2 cell line was maintained at 37 °C, 5% CO_2_, at 90% humidity in MEM supplemented with 10% fetal bovine serum, 1% L-glutamine (200 mM), and penicillin (100 U/mL)/streptomycin (100 μg/mL) (complete RPMI medium). The cytotoxicity of the tested molecules on the HepG2 (hepatocarcinoma cell line purchased from ATCC, ref HB-8065) cell line was assessed according to the method of Mosmann [37] with slight modifications. Briefly, 5.10^3^ cells in 100 μL of complete medium were inoculated into each well of 96-well plates and incubated at 37 °C in humidified 5% CO_2_. After 24 h incubation, 100 μL of medium with various product concentrations dissolved in DMSO (final concentration less than 0.5% *v/v*) were added and the plates were incubated for 72 h at 37 °C. Triplicate assays were performed for each sample. Each plate-well was then microscopically examined for possible precipitate formation before the medium was aspirated from the wells. Next, 100 µL of MTT (3-(4,5-dimethyl-2-thiazolyl)-2,5-diphenyl -2H-tetrazolium bromide) solution (0.5 mg/mL in medium without FBS) was added to each well. Cells were incubated for 2 h at 37 °C. After this time, the MTT solution was removed and DMSO (100 μL) was added to dissolve the resulting blue formazan crystals. Plates were shaken vigorously (700 rpm) for 10 min. The absorbance was measured at 570 nm with 630 nm as reference wavelength using a BIO-TEK ELx808 Absorbance Microplate Reader (LabX, Midland, ON, Canada). DMSO was used as blank and doxorubicin (purchased from Sigma Aldrich) as positive control. Cell viability was calculated as a percentage of control (cells incubated without compound). The 50% cytotoxic concentration (CC_50_) was determined from the dose–response curve, using TableCurve software 2D v.5.0. CC_50_ values to represent the mean value calculated from three independent experiments.

#### 3.2.2. In Vitro Antiplasmodial Evaluation

In this study, a K1 culture-adapted *P. falciparum* strain resistant to chloroquine, pyrimethamine, and proguanil was used in an in vitro culture. It was maintained in continuous culture, as described previously by Trager and Jensen [38]. Cultures were maintained in fresh A+ human erythrocytes at 2.5% hematocrit in complete medium (RPMI 1640 with 25 mM HEPES, 25 mM NaHCO_3_, 10% of A+ human serum) at 37 °C under reduced O2 atmosphere (gas mixture 10% O2, 5% CO_2_, and 85% N_2_). Parasitemia was maintained daily between 1 and 3%. The *P. falciparum* drug susceptibility test was carried out by comparing quantities of DNA in treated and control cultures of parasite in human erythrocytes according to an SYBR Green I fluorescence-based method [39] using a 96-well fluorescence plate reader. Compounds, previously dissolved in DMSO (final concentration less than 0.5% *v/v*), were incubated in a total assay volume of 200 μL (RPMI, 2% hematocrit and 0.4% parasitemia) for 72 h in a humidified atmosphere (10% O_2_ and 5% CO_2_) at 37 °C, in 96-well flat bottom plates. Duplicate assays were performed for each sample. After incubation, plates were frozen at 20 °C for 24 h. Then, the frozen plates were thawed for 1 h at 37 °C. Fifteen µL of each sample were transferred to 96-well flat bottom non-sterile black plates (Greiner Bio-one, Kremsmünster, Austria), already containing 15 µL of the SYBR Green I lysis buffer (2X SYBR Green I, 20 mM Tris base pH 7.5, 20 mM EDTA, 0.008% *w/v* saponin, 0.08% *w/v* Triton X-100). Negative control treated by solvents (DMSO or H_2_O) and positive controls (chloroquine and doxycycline) were added to each set of experiments. Plates were incubated for 15 min at 37 °C and then read on a TECAN Infinite F-200 spectrophotometer with excitation and emission wavelengths at 485 and 535 nm, respectively. The concentrations of compounds required to induce a 50% decrease in parasite growth (EC_50_ K1) were calculated from three independent experiments.

#### 3.2.3. Ames Test

Compounds were assessed for mutagenicity by a modified version of the liquid incubation assay of the classical Ames test at five concentrations (25–125 nM) [40]. *Salmonella* tester strains (TA97a: Acridine ICR191, TA98: 2,4,7 trinitrofluorenone, TA100: Sodium Azide and TA102: Mitomycine C) as positive control w/o S9 mix and benzo[alpha]pyrene as positive control w/S9 mix) were grown overnight in a nutrient broth n_2 (Oxoid, Dardilly, France). After this period, products dissolved in DMSO (Sigma) were added to 0.1 mL of culture and incubated for 1 h at 37 °C with shaking. Each sample was assayed in duplicate. After incubation, 2 mL of molten top agar were mixed gently with the pre-incubated solution and poured onto Vogel–Bonner minimal agar plates. After 48 h at 37 °C, the number of spontaneous and drug-induced revertants per plate was determined for each dose with a laser bacterial colony counter (laser bacterial colony counter 500A, Interscience). A product was considered mutagenic when it induced a two-fold increase in the number of revertants, compared with the spontaneous frequency (negative control). For each *Salmonella* strain, a specific positive- and solvent-control were performed.

#### 3.2.4. Comet Assay

##### Cell Culture and Treatment

The human hepatocarcinoma cell line HepG2 was obtained from the American Type Culture Collection (ATCC, ref. HB-8065). Cells were cultured in Eagle’s minimum essential medium (EMEM, ref. ATCC^®®^ 30-2003TM) and supplemented with 10% heat-inactivated foetal bovine serum, 100 U/mL penicillin, and 0.1 mg/mL streptomycin (all from Gibco). Cells were maintained at 37 °C in a humidified atmosphere with 5% CO_2_ and used in passage number 11 to 15. Two concentrations of the compound 3i (3.2 and 16 µM) were tested at 2 different times of incubation (2 and 72 h). Briefly, HepG2 cells were seeded at 1.13 × 10^5^ cells/mL in 6-well plates (3 mL of cell suspension per well) and incubated at 37 °C in a humidified atmosphere with 5% CO_2_. After 24 and 94 h of incubation, cells were treated with different concentrations of the compound or the vehicle (0.5% dimethylsulfoxide, DMSO) for 72 and 2 h, respectively. Additionally, in the 2 h treatment plate, cells in an additional well were treated with 1 mM MMS as positive control for the comet assay. After treatment, medium was removed from the wells and cells were washed with phosphate buffered saline (PBS). Finally, cells were trypsinized and trypsin was neutralized with fresh medium. From this point, cells were kept ice-cold to avoid DNA repair.

##### Comet Assay

The standard alkaline comet assay was employed for the detection of DNA strand breaks (SBs) and alkali-labile sites (ALS) in cells treated with molecule **3i**. Trypsinized HepG2 cells were centrifuged at 125 × *g* for 5 min at 4 °C and resuspended in cold PBS at 1 x 10^6^ cells/mL. For the preparation of the agarose gels, 30 µL of cell suspension were mixed with 140 µL of 1% low melting point agarose in PBS at 37 °C and 2 aliquots of 70 µL of cell/agarose mixture were placed on agarose-precoated microscope slides. Each droplet was covered with a 20 × 20 mm coverslip and, after 2–3 min on a cold metal plate, the coverslips were removed. Then, slides were immersed in lysis solution (2.5 M NaCl, 0.1 M Na_2_EDTA, 0.01 M Tris base, pH 10, and 1% Triton X-100) at 4 °C for 1 h. After lysis, slides were transferred to the electrophoresis tank and incubated for 40 min at 4 °C in the electrophoresis solution (0.3 M NaOH, 1 mM Na_2_EDTA, pH > 13) to allow DNA unwinding. After that, electrophoresis was carried out at 1 V/cm for 20 min (4 °C). Then, gels were neutralized and washed by immersing the slides in PBS for 10 min and distilled water for another 10 min (both at 4 °C). Gels were then air-dried at room temperature. Comets were stained by adding 30 µL of 1 mg/mL of 4,6- diamidino-2-phenylindole (DAPI) on top of each gel and placing 22 × 22 mm coverslips on top. Slides were incubated with DAPI at room temperature for 30 min before the analysis. The semi-automated image analysis system Comet Assay IV (Instem, Stone, UK) was used to evaluate 50 comets per gel (i.e., 100 comets/condition). The percentage of DNA in tail was the descriptor used for each comet.

##### Statistics

The median percentage of DNA in tail for 50 comets was calculated for each of the duplicate gels in each experiment, and the mean of the two medians were then calculated. The mean percentage of DNA in tail of 3 independent experiments and the standard deviation (SD) were calculated.

#### 3.2.5. Evaluation against ART-Resistant Strain

##### Parasite Culture

The *Plasmodium falciparum* ART-resistant strain F32-ART5 and its tween ART-sensitive strain F32-TEM [33,41] were cultivated at 37 °C with 5% CO_2_ in human red blood cells (EFS, French Blood Bank) diluted to 2% hematocrit in RPMI-1640 medium (Biowest, Nuaillé, France) supplemented with 5% human serum (EFS, French Blood Bank), as previously reported [41].

##### Standard In Vitro Chemo-Sensitivity Assay

The chemosensitivity assay was carried out in 96-well culture plates on synchronized ring-stage parasites at 1% parasitemia obtained after D-sorbitol treatment. The antiplasmodial activities were evaluated using the SYBR Green I method [42]. Parasites were exposed for 48 h to a range of concentrations of the different tested compounds. Each drug concentration was tested in triplicates. Parasite pellets were then washed in 1× PBS prior to lysing red blood cells at −20 °C overnight. Then, the plates were thawed and 100 µL of each well were transferred into a black 96-well plate. Next, 100 µL per well of SYBR Green I (Thermo Fisher) diluted at a final concentration of 2× in lysis buffer (20 mM Tris base pH 7.5, 5 mM EDTA, 0.008% *w/v* saponin, 0.08% *w/v* Triton X-100) were added and left to incubate for 1 h at room temperature prior to reading the plates on BioTek FLx800 Microplate Fluorescence Reader (λexcitation = 485 nm, λemission = 528 nm). Relative EC_50_ values were determined using GraphPad Prism software.

##### Recrudescence Assay

Recrudescence assay was performed to evaluate the ability of the F32-ART5 parasites to survive drug exposure comparatively to the F32-TEM parasites [33,34]. D-sorbitol synchronized ring-stages at 3% parasitemia (2% hematocrit) were exposed to drug for 48 h. After drug exposure, parasite cultures were washed with RPMI-1640 medium and replaced in normal drug-free culture conditions. The time to parasite recrudescence was determined as the time required to reach the 3% initial parasitemia. If no parasites were observed in the following 30 days, the culture was considered “not recrudescent”. Each experiment was performed for F32-ART5 and F32-TEM and cultivated in parallel in the same conditions to generate paired results. This was carried out at least three times independently. A statistical analysis (log-rank Mantel–Cox test) was carried out on data thanks to a Kaplan–Meier survival analysis (using GraphPad Prism software) considering censored data.

##### Quiescent-Stage Survival Assay

Chemosensitivity evaluation of ART-resistant parasites at the quiescent stage was performed on the strain F32-ART5 thanks to the quiescent-stage survival assay (QSA) [35]. D-sorbitol synchronized ring-stages parasites at 3% parasitemia (2% hematocrit) were first exposed to 700 nM of dihydroartemisinin (DHA) for 6 h to induce quiescence of artemisinin-resistant parasites. Then, quiescent parasites were exposed or not (as control condition) to the drug to be tested for 48 h. After drug exposure, parasite cultures were washed with RPMI-1640 medium and replaced in normal drug-free culture conditions. The time to parasite recrudescence was determined as the time required to reach the 3% initial parasitemia. If no parasites were observed in the following 30 days, the culture was considered “not recrudescent”. In each experiment, results obtained with the condition “DHA 6 h/DHA 48 h” were compared to those obtained with the condition “DHA 6 h/(DHA + drug to be tested) 48 h” in order to determine the delay in recrudescence time. This delay is representative of the capacity of the tested compound to be active on quiescent parasites and it is assumed that a 6-day threshold is necessary to classify a compound as active on quiescent parasites [35].

#### 3.2.6. Apicoplast Studies

##### Culturing Plasmodium-Infected Red Blood Cells

*Plasmodium falciparum* blood stage parasites were maintained at 2% hematocrit in 1640 RPMI-HEPES, supplemented with 5% AlbuMAX II (GIBCO) and 0.25% gentamycin. Parasites were grown sealed Perspex chambers gassed with beta mix gas (1% O_2_, 5% CO_2_ and 94% N_2_) at 37 °C and maintained on 48-h cycles. Cultures were tightly synchronized at ring stage using sorbitol treatment (5% *v/v*) as previously described [43].

##### IFA on Treated Parasites

Prior to the treatment, parasites were synchronized using 5% sorbitol. After 48 h treatment (0.3 µM of (3i) molecule, 0.3 µM of (2i) molecule or DMSO) parasites were fixed using 4% paraformaldehyde (PFA) and 0.0075% glutaraldehyde for 30 min at room temperature. Fixing solution was washed 3x times with PBS and cells were permeabilized with 0.1% TX-100 for 10 min at room temperature. The permeabilization solution was washed 3× times with PBS and cells were blocked with 3% fetal bovine serum (FBS) for 1 h. Primary antibody (Rat IgG anti-HA, Roche, 1/500 in 3% FBS) was incubated for 1 h at room temperature. The primary antibody was washed out 3× times with PBS and cells were incubated with secondary antibody (Alexa Fluor 488 goat anti-mouse IgG, Invitrogen, 1/1000 in 3% FBS) for 1 h at room temperature. The secondary antibody was washed out 3× times with PBS and cells are incubated with DAPI, 1/25000 in PBS. The samples were fixed between a slide and a coverslip with fluorogel and observed by epi-fluorescent microscopy.

##### Growth Assay

To observe a potential effect of delayed death of the molecule on the parasite, *Plasmodium* was maintained on three life cycles (48 h). At 48 h, 96 h, and 144 h, 100 µL of cultures were transferred into a 96-wells black wall flat bottom plate and mixed with 100 µL SYBR Green lysis buffer (20 mM Tris, pH 7.5; 5 mM EDTA; 0.008% (*w/v*) saponin; 0.08% (*v/v*) Triton X-100) with freshly added SYBR Green I (10000×), and incubated for 1 h at room temperature protected from the light. Fluorescence from each well was measured with TECAN infinite M200 plate reader (excitation: 485 nm, emission: 538 nm and integration time: 1000 μs). The rest of the cultures were diluted 1/10 as the untreated cultures. A graph was obtained by performing the ration of the treated culture fluorescence intensity on the untreated culture fluorescence intensity (*n* = 3).

#### 3.2.7. Study on the Liver Stage

##### *P. falciparum* Sporozoite Isolation

*P. falciparum* (NF135 strain) sporozoites were isolated by dissection of the salivary glands of infected *A. stephensi* 14–21 days after an infective blood meal (Department of medical microbiology, University Medical Centre, St Radboud, Nijmegen Netherland). All infected salivary glands were removed by hand dissection, crushed in a potter for sporozoites isolation, and filtrated through a 40 μm filter to remove mosquito debris (Cell Strainer, BD BioSciences, Franklin Lakes, NJ, USA). The sporozoites were counted using a disposable Glasstic microscope slide (KOVA, Garden Grove, CA, USA).

##### Hepatocyte Culture and In Vitro Infection with Plasmodium Sporozoites

One day before hepatocyte thawing, 96-well plates were coated by a Corning^®®^ collagen solution (50 µg/mL rat tail type I collagen in 0.02N acid acetic) over night at room temperature. The next day, the wells were washed twice by PBS solution. Cryopreserved primary human hepatocytes (LONZA, Basel, Swiss) were seeded in 96-well plates with a pre-determined seeding dose that gave a single dense layer of attached hepatocytes. Four days post-seeding, each plate well was infected with 30,000 freshly extracted *Plasmodium falciparum* sporozoites. The culture plates were kept at 37 °C with 5% CO_2_ and the Williams E medium (Gibco) containing usual supplements were used to feed the cells. [44]

##### Immunostaining and Confocal Microscopy of Infected Hepatocyte Cultures

Hepatocyte cultures were fixed with 4% paraformaldehyde solution (PFA, Invitrogen) for 10 min. To visualize intracellular *Plasmodium* schizonts anti-serum raised in mice against recombinant *P. falciparum* heat-shock protein 70 (PfHSP70) (1:1000 dilution in PBS-1% BSA-0.3% Triton) and to visualize parasite apicoplast, an antibody against *Plasmodium yoelli* acyl carrier protein (PyACP) (1:250 dilution in PBS-BSA-Triton) was used. Host and parasite nuclei were stained by DAPI. Fixed and immunostained hepatocyte cultures were studied by a confocal (Leica SP8 white laser) microscope located at the ICM Quant microscopy platform (Institut du Cerveau et de la Moelle, ICM) in Paris, France. Images were analyzed and prepared with ImageJ software. Apicoplast morphology was observed and quantified by eye under a confocal microscope.

##### Quantification of Parasite Size and Numbers in Treated Versus Control Wells

Upon immunostaining of parasites, the study plates were scanned using a CellInsight high-content screening platform equipped with the Studio HCS software (Thermo Fisher Scientific, Waltham, MA, USA). The details of analysis is provided elsewhere [45]. The number of host hepatocyte nuclei per well was counted by the system as a measure of toxicity of 3i towards the liver cells.

## 4. Conclusions

An antiplasmodial SAR study, against the asexual erythrocytic stage, was conducted through the synthesis and evaluation of 20 original 3-trichloromethylquinoxaline derivatives. All the molecules showed good in vitro antiplasmodial activity. Among them, compound (**3i**) had the best EC_50_ values against the K1 *P. falciparum* strain along with a low cytotoxicity on the HepG2 cell line (SI = 160) and no cytotoxicity on human hepatocytes. Nitro-containing (**3i**) was not mutagenic in the Ames test or genotoxic in the in vitro comet assay. The CCl_3_ group was found to be essential for antiplasmodial activity. Moreover, (**3i**) showed an effect on the apicoplast biogenesis, inducing a quick-killing mechanism. A parasite chemical rescue strategy could address the question of whether (**3i**) targets the apicoplast as a primary target or an off-target. The essential metabolic pathways that reside in the apicoplast are attractive targets for the development of new antimalarial drugs with novel mechanisms of action.

## Figures and Tables

**Table 1:**
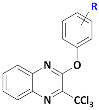
Reaction yields, antiplasmodial activity, HepG2 cytotoxicity, and clogP of the 3-phenoxy-2-trichloromethylquinoxalines **3a–3p**.

R	N°	Yield Step 2 (%)	EC_50_ *Pf*K1 (µM) ± SD	CC_50_ HepG2 (µM) ± SD	SI ^d^	clogP ^e^
H	**3a**	84	0.47 ± 0.10	45.3 ± 9.6	96.4	4.28
2-Cl	**3b**	67	0.55 ± 0.12	49.5 ± 10.1	90.0	4.75
3-Cl	**3c**	67	0.34 ± 0.06	37.2 ± 8.1	109.4	4.77
2,4-Cl	**3d**	52	0.60 ± 0.12	37.7 ± 4.7	62.8	5.28
2-F	**3e**	43	0.64 ± 0.14	>15.6 ^c^	>24.4	4.53
3-F	**3f**	52	0.32 ± 0.07	33.5 ± 6.3	105.0	4.54
4-F	**3g**	58	0.42 ± 0.09	35.9 ± 7.5	85.5	4.56
4-OMe	**3h**	27	0.38 ± 0.08	>7.8 ^c^	>20.6	4.22
4-NO_2_^f^	**3i**	57	0.20 ± 0.04	32.0 ± 6.1	160.0	3.53
4-CF_3_	**3j**	70	0.30 ± 0.06	28.3 ± 4.4	94.3	5.27
4-OCF_3_	**3k**	85	0.40 ± 0.08	27.1 ± 4.7	67.8	5.15
4-SF_5_	**3l**	84	0.20 ± 0.03	16.6 ± 3.9	83.0	5.41
4-SCF_3_	**3m**	58	0.20 ± 0.04	25.6 ± 4.6	128.0	5.70
4-CN	**3n**	81	0.25 ± 0.04	15.4 ± 2.1	77.0	4.06
4-SO_2_CF_3_	**3o**	26	0.22 ± 0.07	7.2 ± 1.4	32.7	4.77
4-SONHCF_3_	**3p**	28	0.21 ± 0.05	6.5 ± 1.4	31.0	5.11
Chloroquine ^a^			0.80	30.0	37.5	3.82
Artesunate ^a^			0.003 ± 0.0012	14.7 ± 1.4	4900	-
Doxycycline ^a^			6.00	20.0	3.3	-
Doxorubicin ^b^			-	0.2	-	-

^a^ Chloroquine, artesunate, and doxycycline were used as antimalarial reference-drugs; ^b^ Doxorubicin was used as a cytotoxic reference-drug; ^c^ Highest concentrations were tested due to a lack of solubility; ^d^ Selectivity index (SI) was calculated according to the formula: SI = CC_50_/EC_50_; ^e^ Weighted clogP was computed by the SwissADME; ^f^ The best antiplasmodial hit with the best ratio activity/cytotoxicity.

**Table 2:**
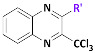
Reaction yields, antiplasmodial activity, HepG2 cytotoxicity, and clogP of the cluttered 3-phenoxy-2-trichloromethylquinoxalines **3q** to **3t**.

R’	N°	Yield Step 2 (%)	EC_50_ *Pf*K1 (µM) ± SD	CC_50_ HepG2 (µM) ± SD	SI ^d^	clogP ^e^
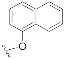	**3q**	43	0.60 ± 0.10	53.1 ± 7.6	88.5	5.17
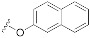	**3r**	64	0.40 ± 0.08	>31.2 ^c^	>78.1	5.19
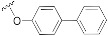	**3s**	50	0.60 ± 0.10	>62.5 ^c^	>104.2	5.61
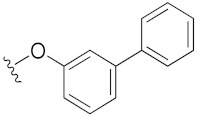	**3t**	70	0.50 ± 0.05	>31.2 ^c^	>62.5	5.60
Chloroquine ^a^			0.80	30.0	37.5	3.82
Artesunate ^a^			0.003 ± 0.0012	14.7 ± 1.4	4900	-
Doxycycline ^a^			6.00	20.0	3.3	-
Doxorubicin ^b^			-	0.2	-	-

^a^ Chloroquine, artesunate and doxycycline were used as antimalarial reference-drugs; ^b^ Doxorubicin was used as a cytotoxic reference-drug; ^c^ Highest concentration tested due to a lack of solubility; ^d^ Selectivity index (SI) was calculated according to the formula: SI = CC_50_/EC_50_, ^e^ Weighted clogP was computed by the SwissADME.

**Table 3:** Ames test and in vitro comet assay data regarding hit compound **3i**.

	Time (h)	[3i] Concentration	Result
**Ames test ^a^**	48	5 mM (w/o S9 mix) 25 mM (w/o S9mix) 5 mM (w/S9 mix) 25 mM (w/S9mix)	negative
**In vitro comet assay ^b^**	2	3.2 µM	negative
	2	16 µM	
	72	3.2 µM	
	72	16 µM	

^a^ Ames test: each concentration of [**3i**] tested at 2, 3, 4 et 5 µL/plate (w/o S9 mix assay) or 4, 6, 8, 10 µL/plate (w/S9 mix assay). ^b^ In vitro comet assay was performed on HepG2 cell line.

**Table 4:** Evaluation of chemo-sensitivity, by standard assay, of *P. falciparum* strains F32-ART5 (ART-resistant) and F32-TEM (ART-sensitive) to (**3i)**. Artemisinin was used as drug control.

	Mean ± SEM EC_50_ values		
	F32-ART5 *n* = 4 *	F32-TEM *n* = 2 *	*p*-value ^$^
3i	400 ± 105 nM	392 ± 34 nM	0.8
Artemisinin	11 ± 3 nM	19 ± 3 nM	0.267

* Results represent the mean of four and two independent experiments for F32-ART5 and F32-TEM, respectively.^$^ Mann–Whitney test. A *p*-value < 0.05 was considered as statistically significant.

## Data Availability

Data sharing not applicable.

## Supplementary Materials

The following are available online at https://www.mdpi.com/article/10.3390/ph14080724/s1, Figures S1–S48: ^1^H and ^13^C NMR spectra, Figure S49: Evaluation of (**3i**) on the ART-resistant strain F32-ART5, Figure S50: Quantification of *P. falciparum* hepatic exo-erythrocytic forms (EEFs) number (A) and size (B) in hepatocyte culture plate fixed at day 6 post-infection, Figure S51: Quantification of *P. falciparum* hepatic exo-erythrocytic forms (EEFs) number (A) and size (B) in hepatocyte culture plate fixed at day 12 post-infection, Figure S52: The effect of (**3i**) on apicoplast in liver stage development of *P. falciparum*.
