# Supplementary material for: 2-Phenoxy-3-Trichloromethylquinoxalines Are Antiplasmodial Derivatives with Activity against the Apicoplast of Plasmodium falciparum
Source: Pharmaceuticals (Basel). 2021 Jul 26;14(8):724. doi: 10.3390/ph14080724 (PMC8400257; doi:10.3390/ph14080724)
Supplement: Supplementary file 1 [file pharmaceuticals-14-00724-s001.zip › pharmaceuticals-1305327-supplementary.pdf]

2-Phenoxy-3-trichloromethylquinoxalines are antiplasmodial derivatives with activity against the apicoplast of *P. falciparum.*

Dyhia Amrane[1], Christophe-Sébastien Arnold[2], Sébastien Hutter[3], Julen Sanz-Serrano[4], Miguel Collia[4], Amaya Azqueta[4,5], Lucie Paloque[6,7,8], Anita Cohen[3], Nadia Amazougaghene[9], Shahin Tajeri[9], Jean-François Franetich[9], Dominique Mazier[9], Françoise Benoit-Vical[6,7,8], Pierre Verhaeghe[6,10], Nadine Azas[3], Patrice Vanelle[1,11*], Cyrille Botté[2,*] and Nicolas Primas[1,11*]

[1] Aix Marseille Univ, CNRS, ICR UMR 7273, Equipe Pharmaco-Chimie Radicalaire, Faculté de Pharmacie, 13385 Marseille cedex 05, France

[2] ApicoLipid Team, Institute for Advanced Biosciences, Université Grenoble Alpes, La Tronche, France.

[3] Aix Marseille Univ, IHU Méditerranée Infection, UMR VITROME, IRD, SSA, Mycology & Tropical Eucaryotic pathogens, 13005 Marseille cedex 05, France

[4] Department of Pharmacology and Toxicology, Faculty of Pharmacy and Nutrition, University of Navarra, C/ Irunlarrea 1, CP 31008, Pamplona, Navarra, Spain

[5] Navarra Institute for Health Research, IdiSNA, Irunlarrea 3, 31008 Pamplona, Spain

[6] LCC-CNRS, Université de Toulouse, CNRS UPR8241, UPS, 31400 Toulouse, France

[7] New antimalarial molecules and pharmacological approaches, MAAP, Inserm ERL 1289, Toulouse, France

[8] Institut de Pharmacologie et de Biologie Structurale, IPBS, Université de Toulouse, CNRS, UPS, France.

[9] Sorbonne Université, INSERM, CNRS, Centre d'Immunologie et des Maladies Infectieuses, CIMI, 75013 Paris, France

[10] CHU de Toulouse, Service Pharmacie, 330 avenue de Grande-Bretagne, 31059 Toulouse cedex 9, France

[11] APHM, Hôpital Conception, Service Central de la Qualité et de l'Information Pharmaceutiques, 13005 Marseille, France

Correspondence: cyrille.botte@univ-grenoble-alpes.fr (C.B.); patrice.vanelle@univ-amu.fr (P.V.) ; nicolas.primas@univ-amu.fr (N.P.)

Supplementary material:

1. **Experimental spectra of final compounds** **3**
2. **Evaluation on artemisinin-resistant and artemisinin-sensitive parasites** **27**
3. **Studying the effect of (3i) on the liver stage development of *P. falciparum*** **28**
4. **The effect of (3i) on apicoplast in liver stage development of *P. falciparum*** **29**

Table of contents:

Figure S1. ^{1}H NMR spectrum of **3a** in $CDCl_3$, on a Bruker ARX 250 spectrometer. 3
Figure S2. ^{13}C NMR spectrum of **3a** in $CDCl_3$, on a Bruker ARX 63 spectrometer. 4
Figure S3. ^{1}H NMR spectrum of **3b** in $CDCl_3$, on a Bruker ARX 250 spectrometer. 4
Figure S4. ^{13}C NMR spectrum of **3b** in $CDCl_3$, on a Bruker ARX 63 spectrometer 5
Figure S5. ^{1}H NMR spectrum of **3c** in $CDCl_3$, on a Bruker ARX 250 spectrometer................................ 5
Figure S6. ^{13}C NMR spectrum of **3c** in $CDCl_3$, on a Bruker ARX 63 spectrometer................................ 6
Figure S7. ^{1}H NMR spectrum of **3d** in $CDCl_3$, on a Bruker ARX 250 spectrometer. 6
Figure S8. ^{13}C NMR spectrum of **3d** in $CDCl_3$, on a Bruker ARX 63 spectrometer................................ 7
Figure S9. ^{1}H NMR spectrum of **3e** in $CDCl_3$, on a Bruker ARX 250 spectrometer................................ 7

Figure S10. ^{13}C NMR spectrum of **3e** in $CDCl_3$, on a Bruker ARX 63 spectrometer 8
Figure S11. ^{1}H NMR spectrum of **3f** in $CDCl_3$, on a Bruker ARX 250 spectrometer 8
Figure S12. ^{13}C NMR spectrum of **3f** in $CDCl_3$, on a Bruker ARX 63 spectrometer 9
Figure S13. ^{1}H NMR spectrum of **3g** in $CDCl_3$, on a Bruker ARX 250 spectrometer 9
Figure S14. ^{13}C NMR spectrum of **3g** in $CDCl_3$, on a Bruker ARX 63 spectrometer 10
Figure S15. ^{1}H NMR spectrum of **3h** in $CDCl_3$, on a Bruker ARX 250 spectrometer 10
Figure S16. ^{13}C NMR spectrum of **3h** in $CDCl_3$, on a Bruker ARX 63 spectrometer 11
Figure S17. ^{1}H NMR spectrum of **3i** in $CDCl_3$, on a Bruker ARX 400 spectrometer 11
Figure S18. ^{13}C NMR spectrum of **3i** in $CDCl_3$, on a Bruker ARX 101 spectrometer 12
Figure S19. ^{1}H NMR spectrum of **3j i**n $CDCl_3$, on a Bruker ARX 250 spectrometer 12
Figure S20. ^{13}C NMR spectrum of **3j** in $CDCl_3$, on a Bruker ARX 63 spectrometer 13
Figure S21. ^{1}H NMR spectrum of **3k** in $CDCl_3$, on a Bruker ARX 250 spectrometer 13
Figure S22. ^{13}C NMR spectrum of **3k** in $CDCl_3$, on a Bruker ARX 63 spectrometer 14
Figure S23. ^{1}H NMR spectrum of **3l** in $CDCl_3$, on a Bruker ARX 250 spectrometer 14
Figure S24. ^{13}C NMR spectrum of **3l** in $CDCl_3$, on a Bruker ARX 63 spectrometer 15
Figure S25. ^{1}H NMR spectrum of **3m** in $CDCl_3$, on a Bruker ARX 250 spectrometer 15
Figure S26. ^{13}C NMR spectrum of **3m** in $CDCl_3$, on a Bruker ARX 63 spectrometer 16
Figure S27. ^{1}H NMR spectrum of **3n** in $CDCl_3$, on a Bruker ARX 250 spectrometer 16
Figure S28. ^{13}C NMR spectrum of **3n** in $CDCl_3$, on a Bruker ARX 63 spectrometer 17
Figure S29. ^{1}H NMR spectrum of **3o** in $CDCl_3$, on a Bruker ARX 250 spectrometer 17
Figure S30. ^{13}C NMR spectrum of **3o** in $CDCl_3$, on a Bruker ARX 63 spectrometer 18
Figure S31. ^{1}H NMR spectrum of **3q** in $CDCl_3$, on a Bruker ARX 250 spectrometer 18
Figure S32. ^{13}C NMR spectrum of **3q** in $CDCl_3$, on a Bruker ARX 63 spectrometer 19
Figure S33. ^{1}H NMR spectrum of **3r** in $CDCl_3$, on a Bruker ARX 250 spectrometer 19
Figure S34. ^{13}C NMR spectrum of **3r** in $CDCl_3$, on a Bruker ARX 63 spectrometer 20
Figure S35. ^{1}H NMR spectrum of **3s** in $CDCl_3$, on a Bruker ARX 250 spectrometer 20
Figure S36. ^{13}C NMR spectrum of **3s** in $CDCl_3$, on a Bruker ARX 63 spectrometer 21
Figure S37. ^{1}H NMR spectrum of **3t** in $CDCl_3$, on a Bruker ARX 250 spectrometer 21
Figure S38. ^{13}C NMR spectrum of **3t** in $CDCl_3$, on a Bruker ARX 63 spectrometer 22
Figure S39. ^{1}H NMR spectrum of **3p** in $CDCl_3$, on a Bruker ARX 250 spectrometer 22
Figure S40. ^{13}C NMR spectrum of **3p** in $CDCl_3$, on a Bruker ARX 63 spectrometer 23
Figure S41. ^{1}H NMR spectrum of **3u** in $CDCl_3$, on a Bruker ARX 250 spectrometer 23
Figure S42. ^{13}C NMR spectrum of **3u** in $CDCl_3$, on a Bruker ARX 63 spectrometer 24
Figure S43. ^{1}H NMR spectrum of **3v** in $CDCl_3$, on a Bruker ARX 250 spectrometer 24
Figure S44. ^{13}C NMR spectrum of **3v** in $CDCl_3$, on a Bruker ARX 63 spectrometer 25
Figure S45. ^{1}H NMR spectrum of **3w** in $CDCl_3$, on a Bruker ARX 250 spectrometer 25
Figure S46. ^{13}C NMR spectrum of **3w** in $CDCl_3$, on a Bruker ARX 63 spectrometer 26
Figure S47. ^{1}H NMR spectrum of **2i** in $CDCl_3$, on a Bruker ARX 250 spectrometer 26
Figure S48. ^{13}C NMR spectrum of **2i** in $CDCl_3$, on a Bruker ARX 101 spectrometer 27
Figure S49. Evaluation of (3i) on the ART-resistant strain F32-ART5. 28
Figure S50. Quantification of *P. falciparum* hepatic exo-erythrocytic forms (EEFs) number (A) and size (B) in hepatocyte culture plate fixed at day 6 post-infection. 29
Figure S51. Quantification of *P. falciparum* hepatic exo-erythrocytic forms (EEFs) number (A) and size (B) in hepatocyte culture plate fixed at day 12 post-infection. 29
Figure 52. The effect of **3i** on apicoplast in liver stage development of *P. falciparum* 30

1. Experimental spectra of final compounds

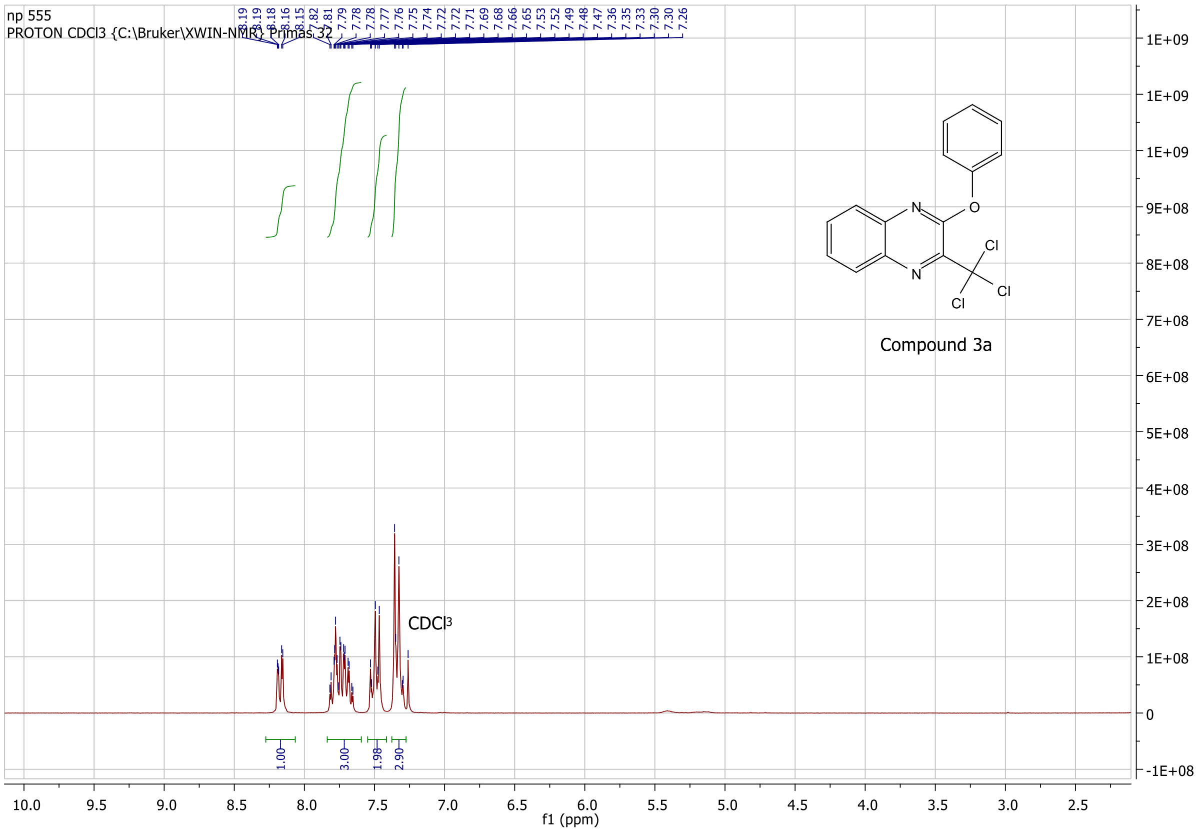

*Figure S1. ^{1}H NMR spectrum of **3a** in $CDCl_3$, on a Bruker ARX 250 spectrometer.*

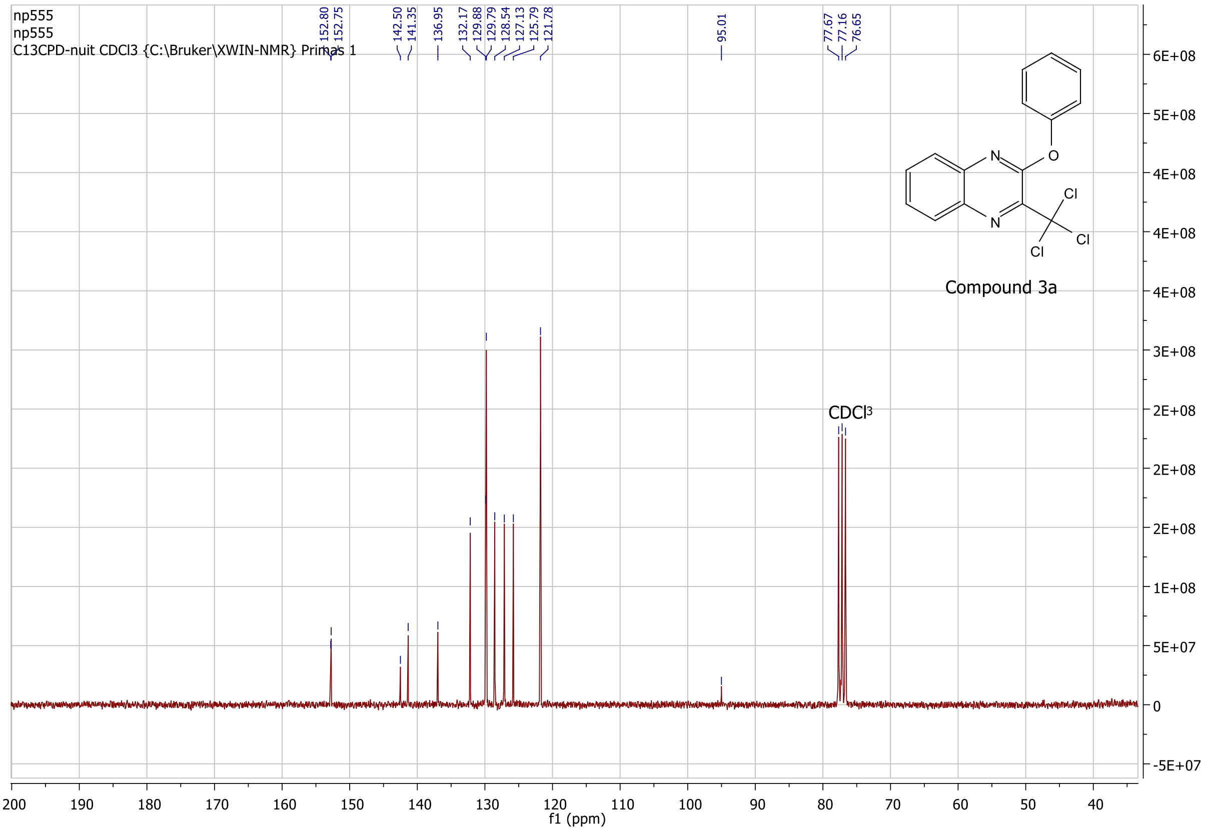

*Figure S2. ^{13}C NMR spectrum of **3a** in $CDCl_3$, on a Bruker ARX 63 spectrometer.*

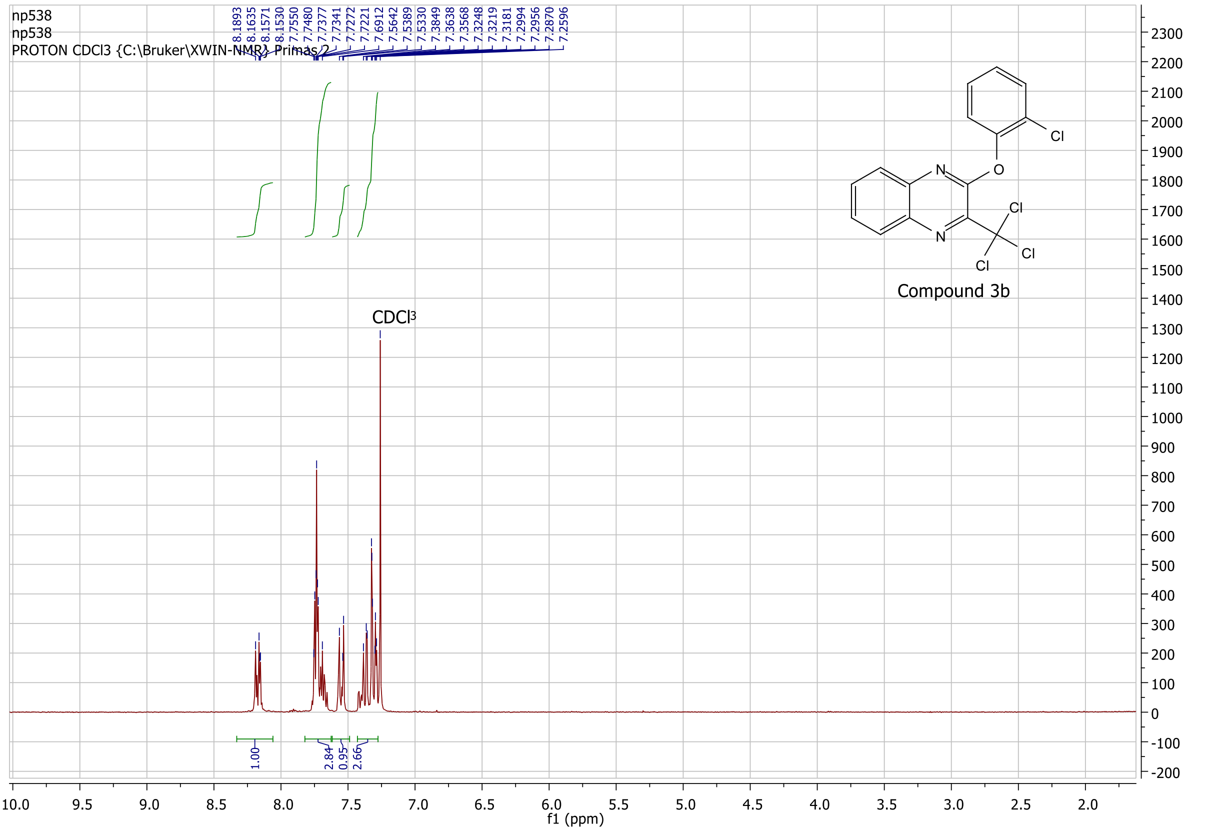

*Figure S3. ^{1}H NMR spectrum of **3b** in $CDCl_3$, on a Bruker ARX 250 spectrometer.*

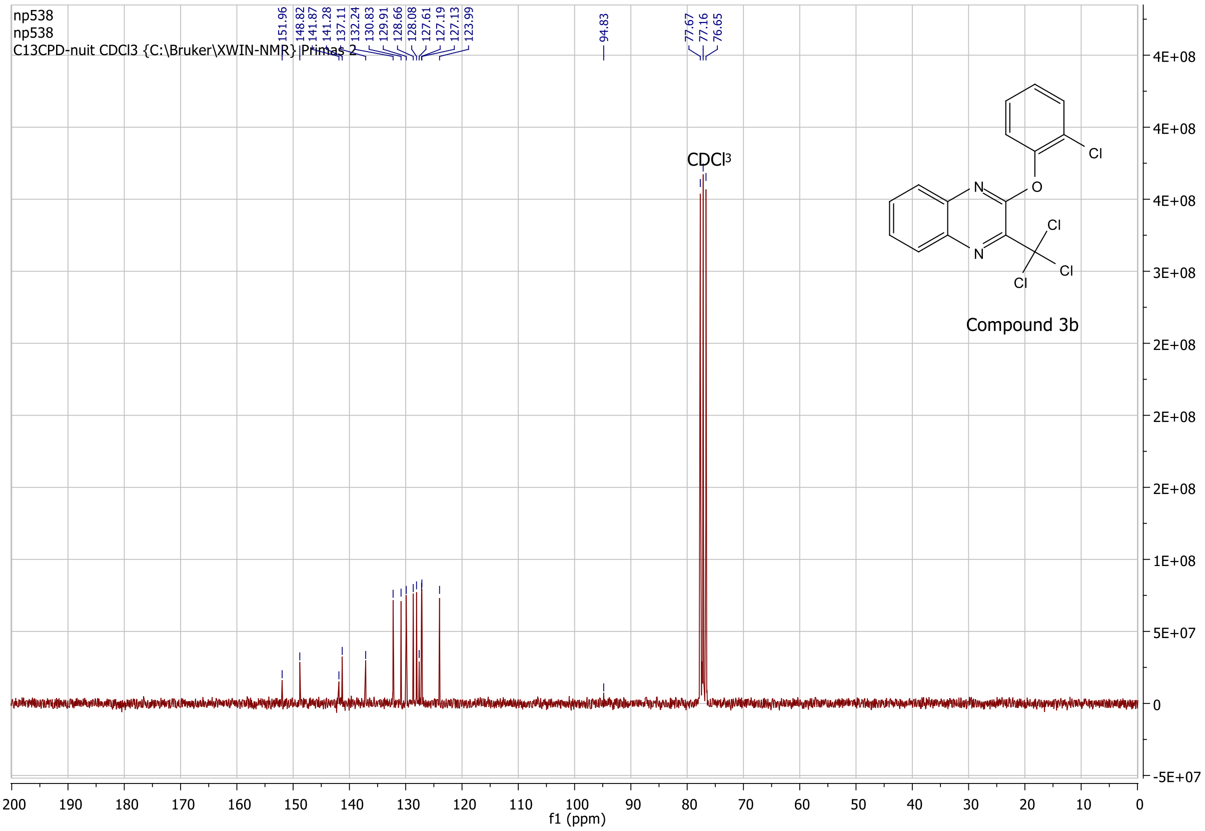

*Figure S4. ^{13}C NMR spectrum of **3b** in $CDCl_3$, on a Bruker ARX 63 spectrometer*

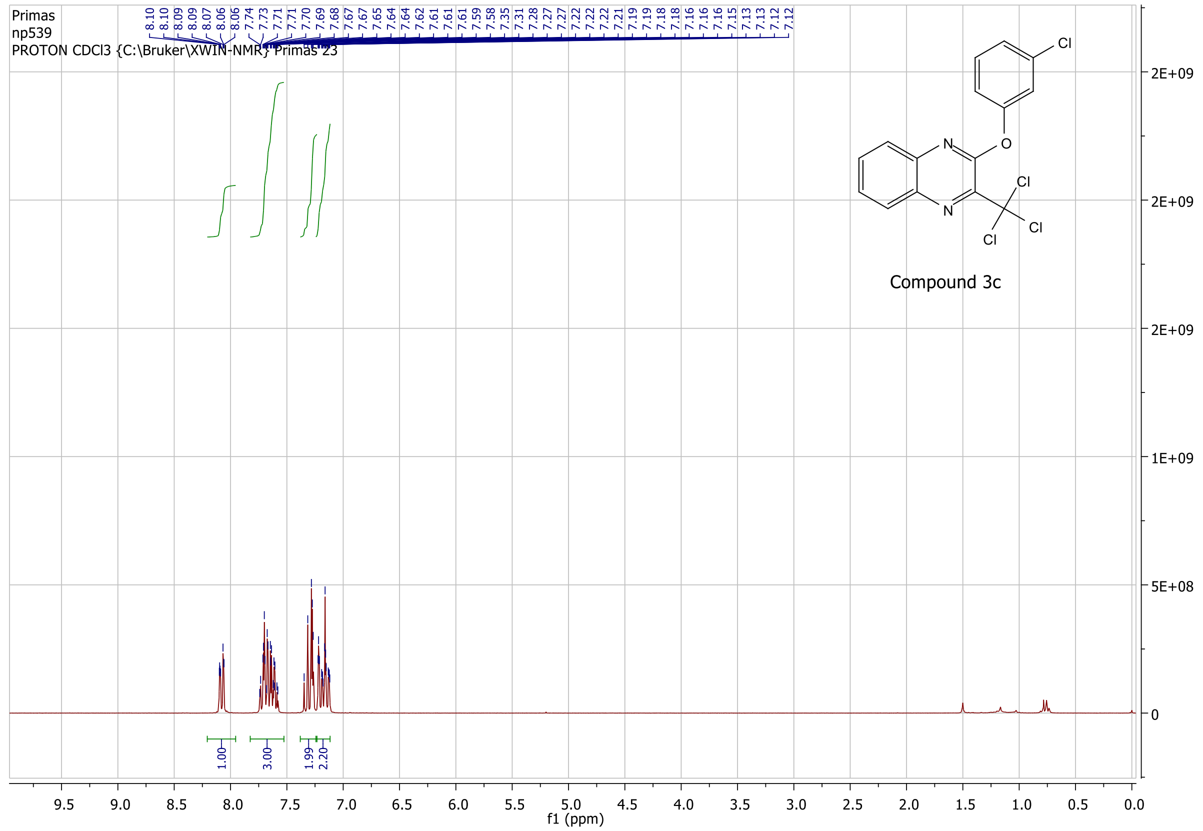

*Figure S5. ^{1}H NMR spectrum of **3c** in $CDCl_3$, on a Bruker ARX 250 spectrometer.*

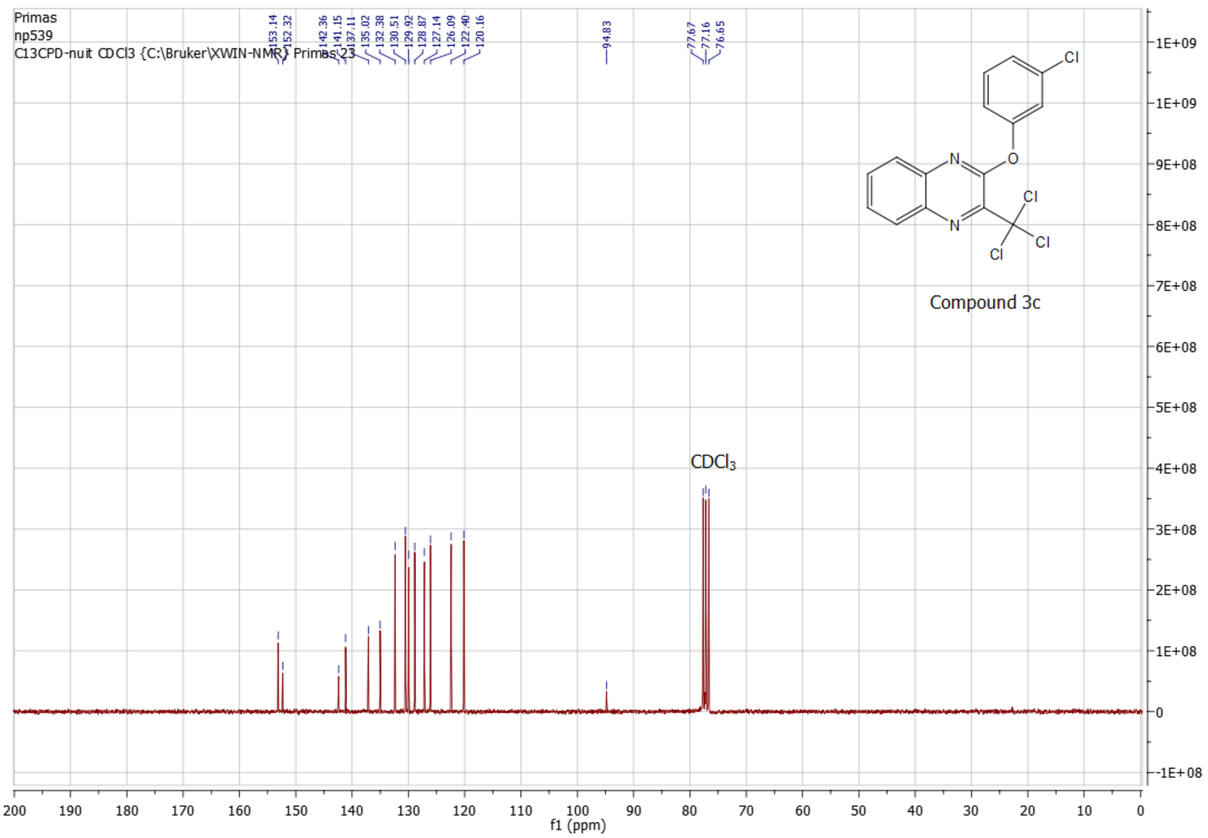

*Figure S6. ^{13}C NMR spectrum of **3c** in $CDCl_3$, on a Bruker ARX 63 spectrometer*

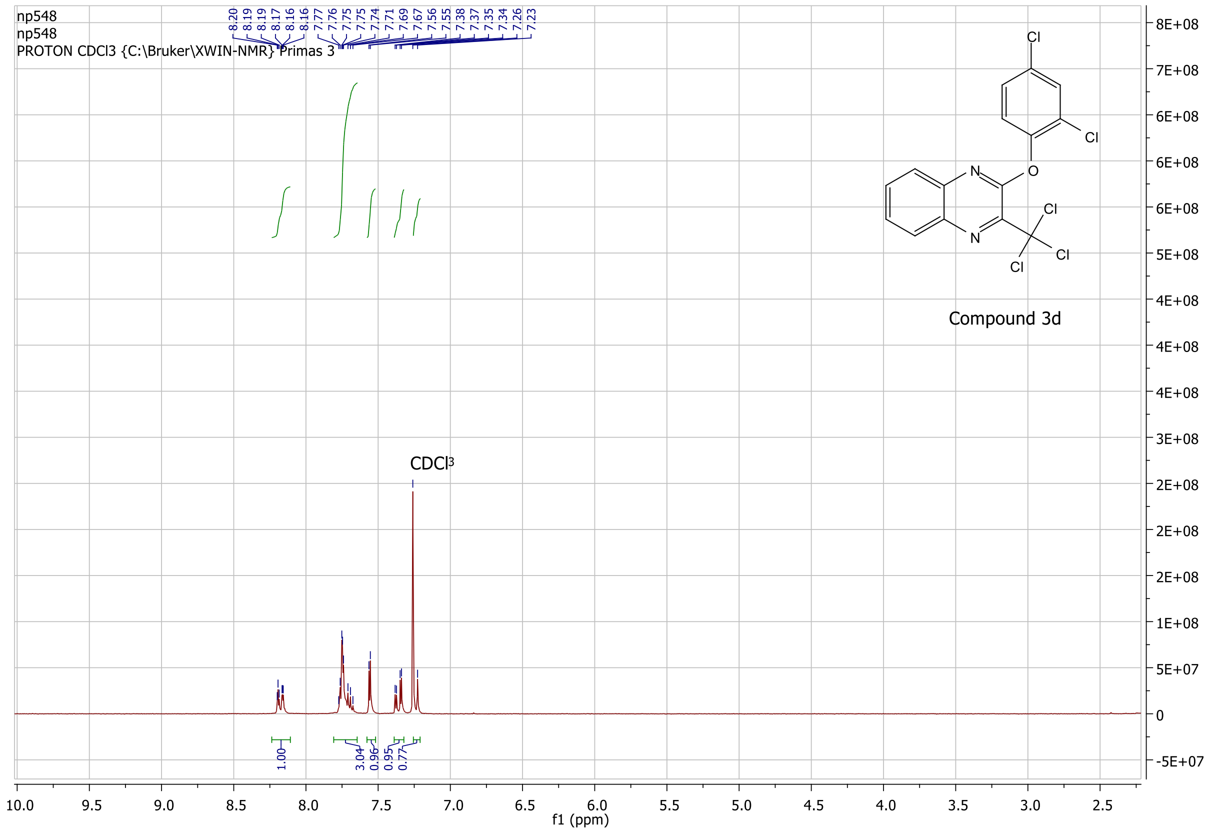

*Figure S7. ^{1}H NMR spectrum of **3d** in $CDCl_3$, on a Bruker ARX 250 spectrometer.*

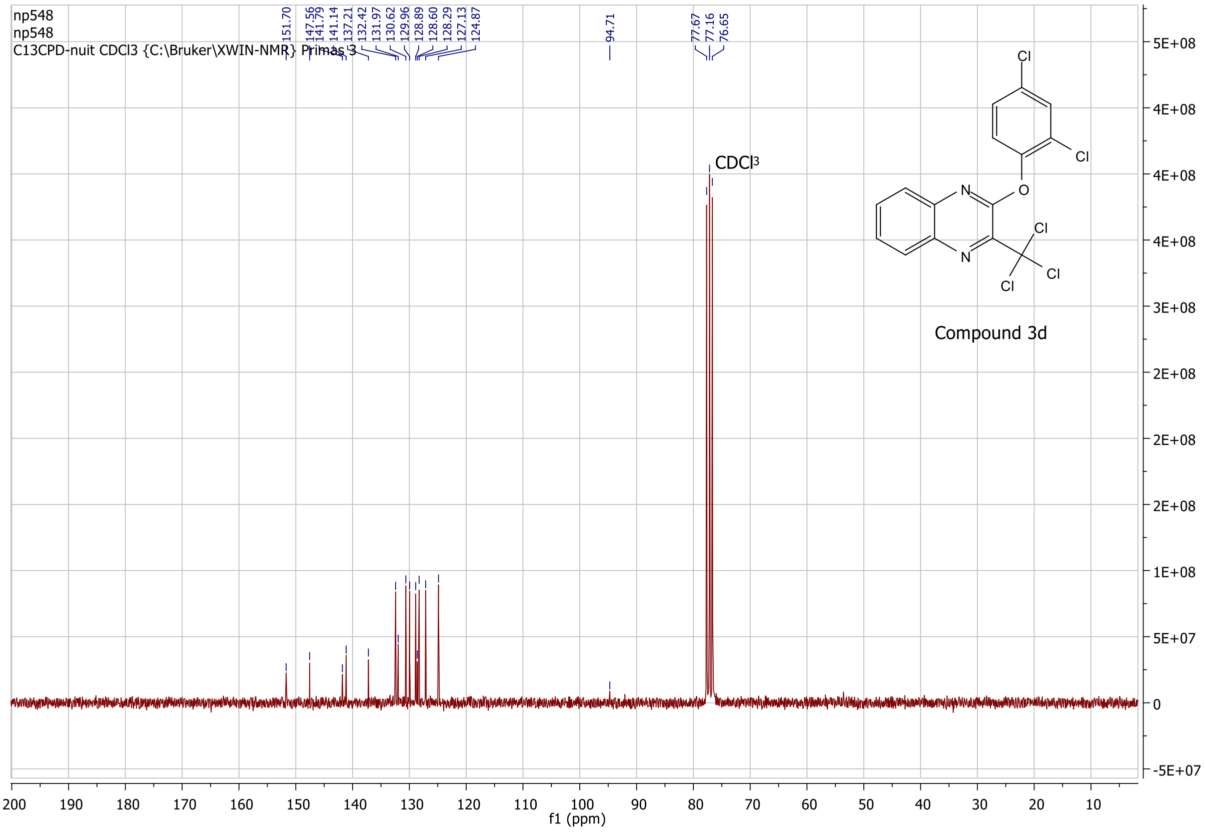

*Figure S8. ^{13}C NMR spectrum of **3d** in $CDCl_3$, on a Bruker ARX 63 spectrometer*

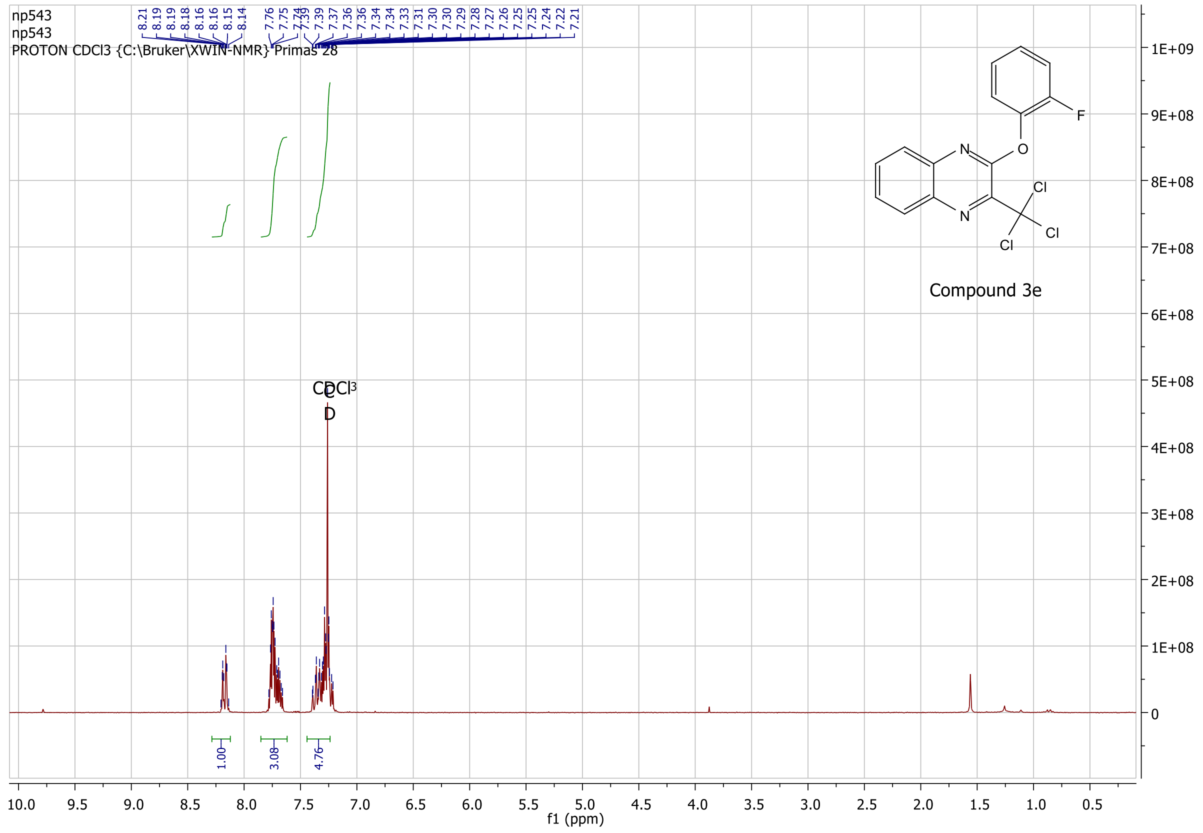

*Figure S9. ^{1}H NMR spectrum of **3e** in $CDCl_3$, on a Bruker ARX 250 spectrometer*

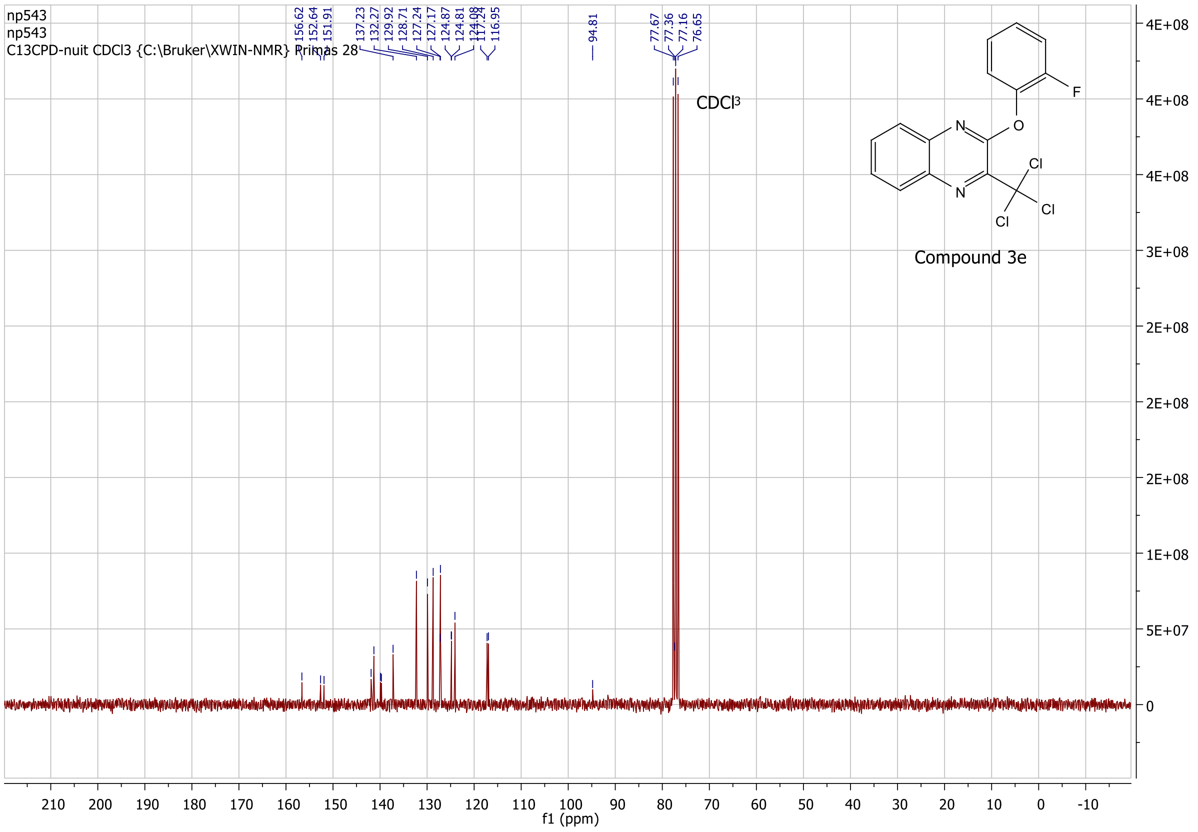

*Figure S10. ^{13}C NMR spectrum of **3e** in $CDCl_3$, on a Bruker ARX 63 spectrometer*

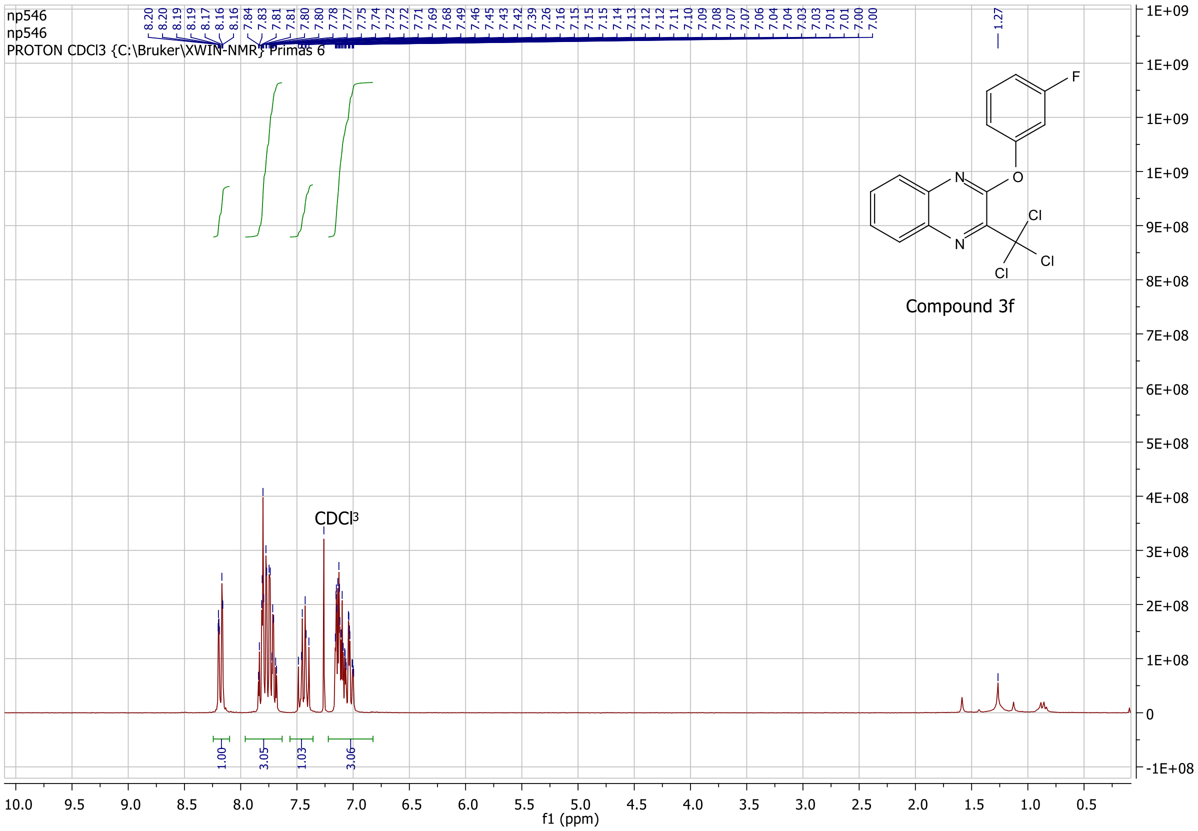

*Figure S11. ^{1}H NMR spectrum of **3f** in $CDCl_3$, on a Bruker ARX 250 spectrometer*

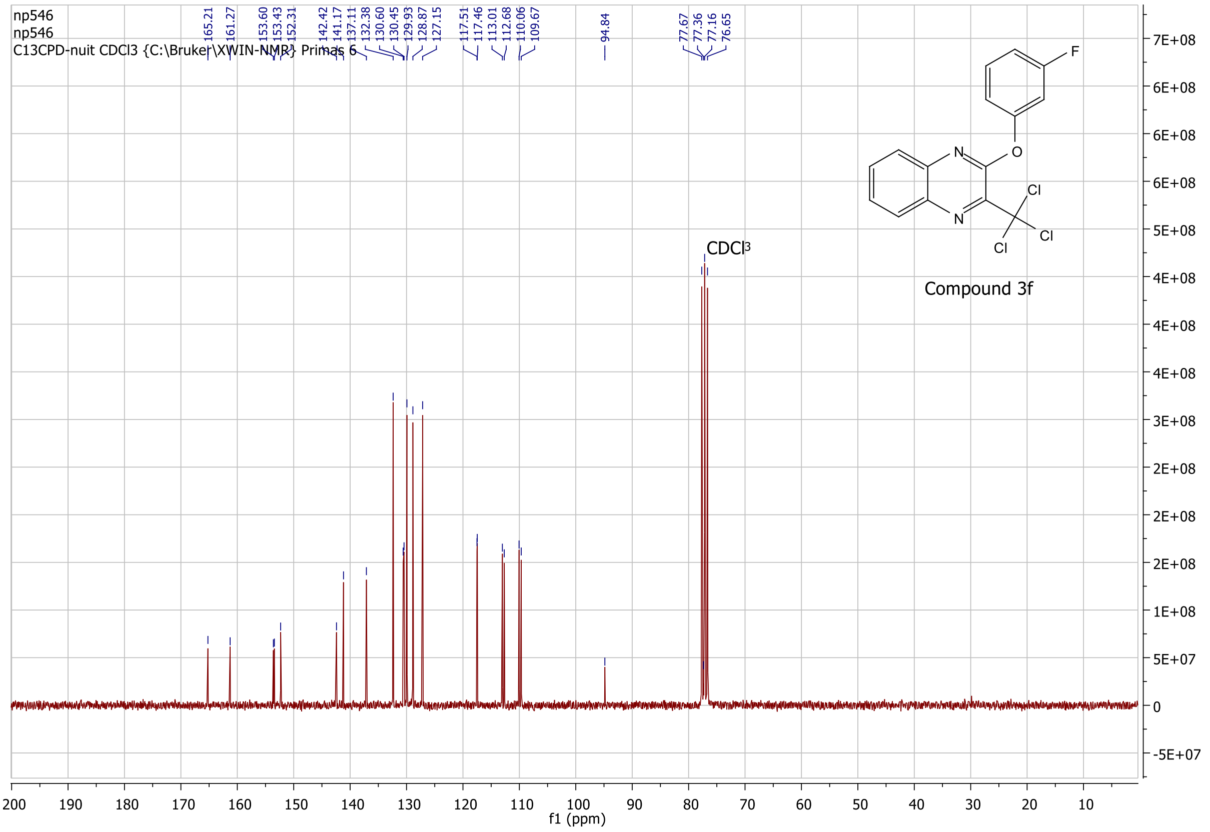

*Figure S12. ^{13}C NMR spectrum of **3f** in $CDCl_3$, on a Bruker ARX 63 spectrometer*

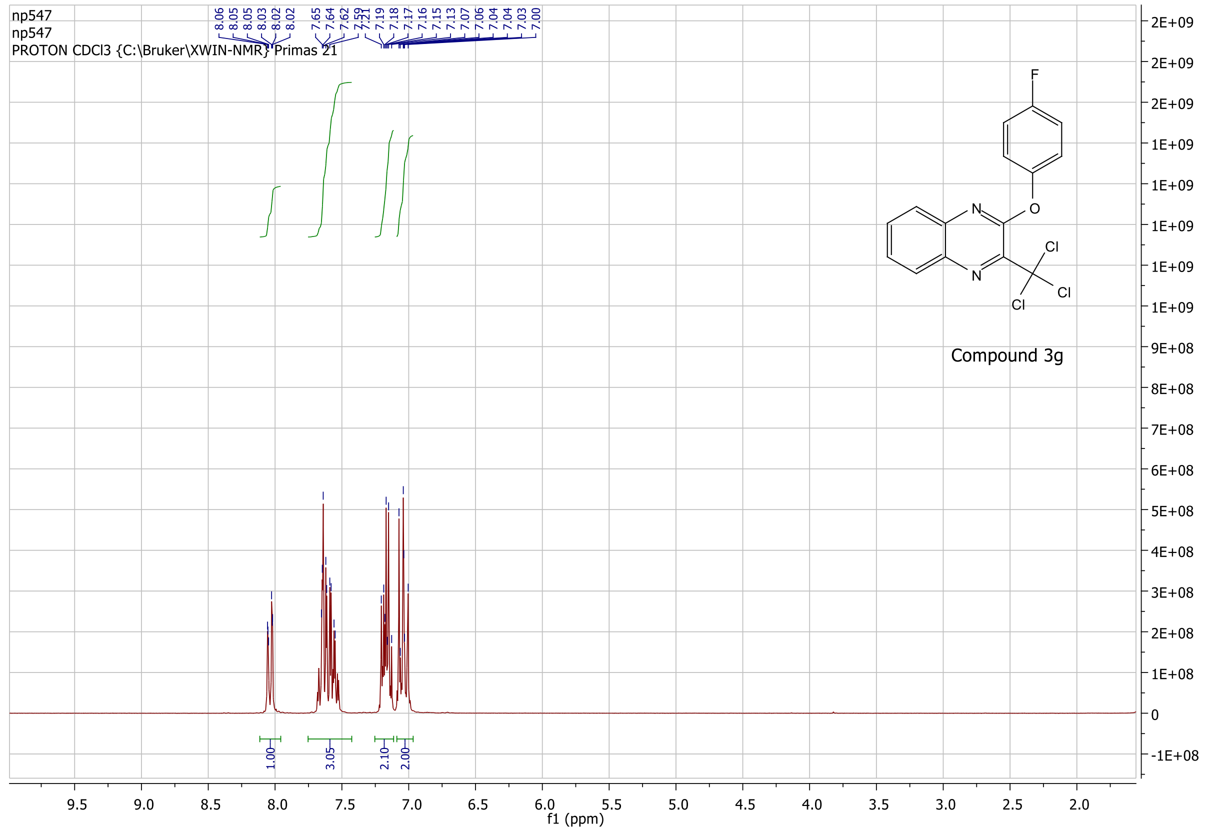

*Figure S13. ^{1}H NMR spectrum of **3g** in $CDCl_3$, on a Bruker ARX 250 spectrometer*

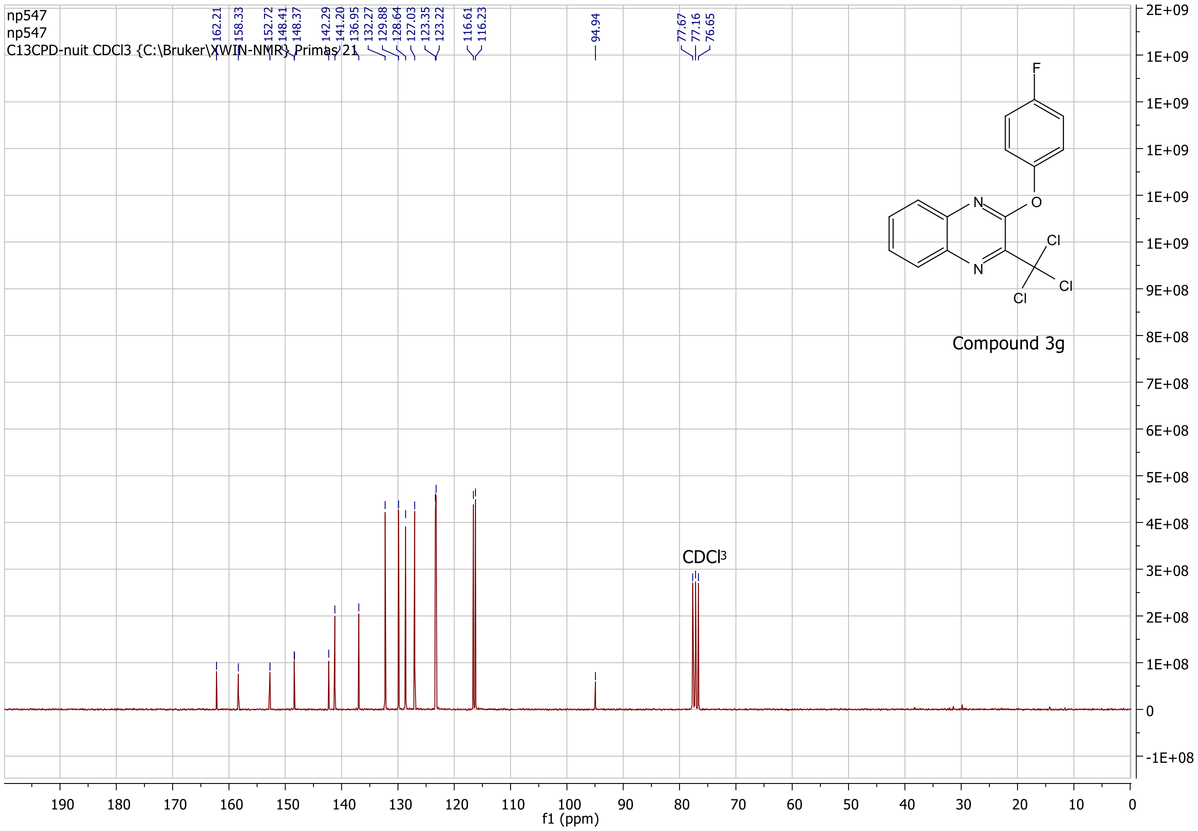

*Figure S14. ^{13}C NMR spectrum of **3g** in $CDCl_3$, on a Bruker ARX 63 spectrometer*

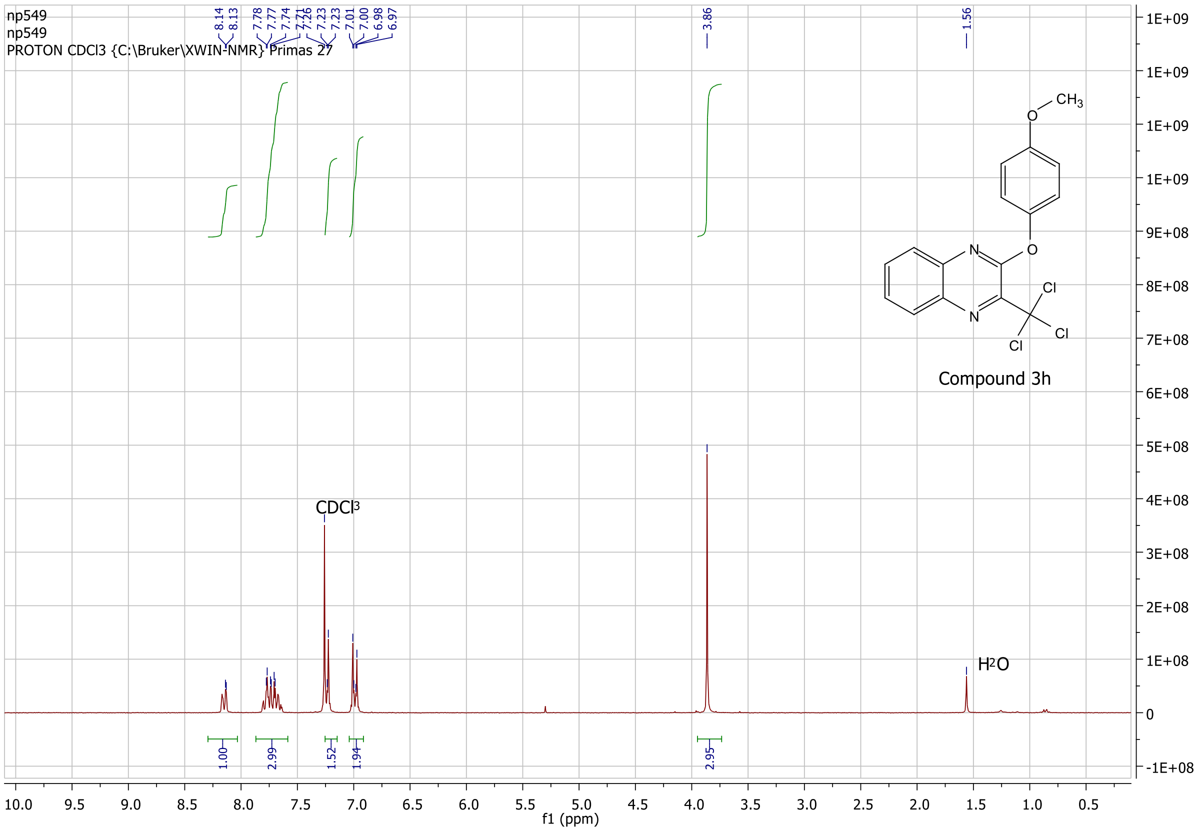

*Figure S15. ^{1}H NMR spectrum of **3h** in $CDCl_3$, on a Bruker ARX 250 spectrometer*

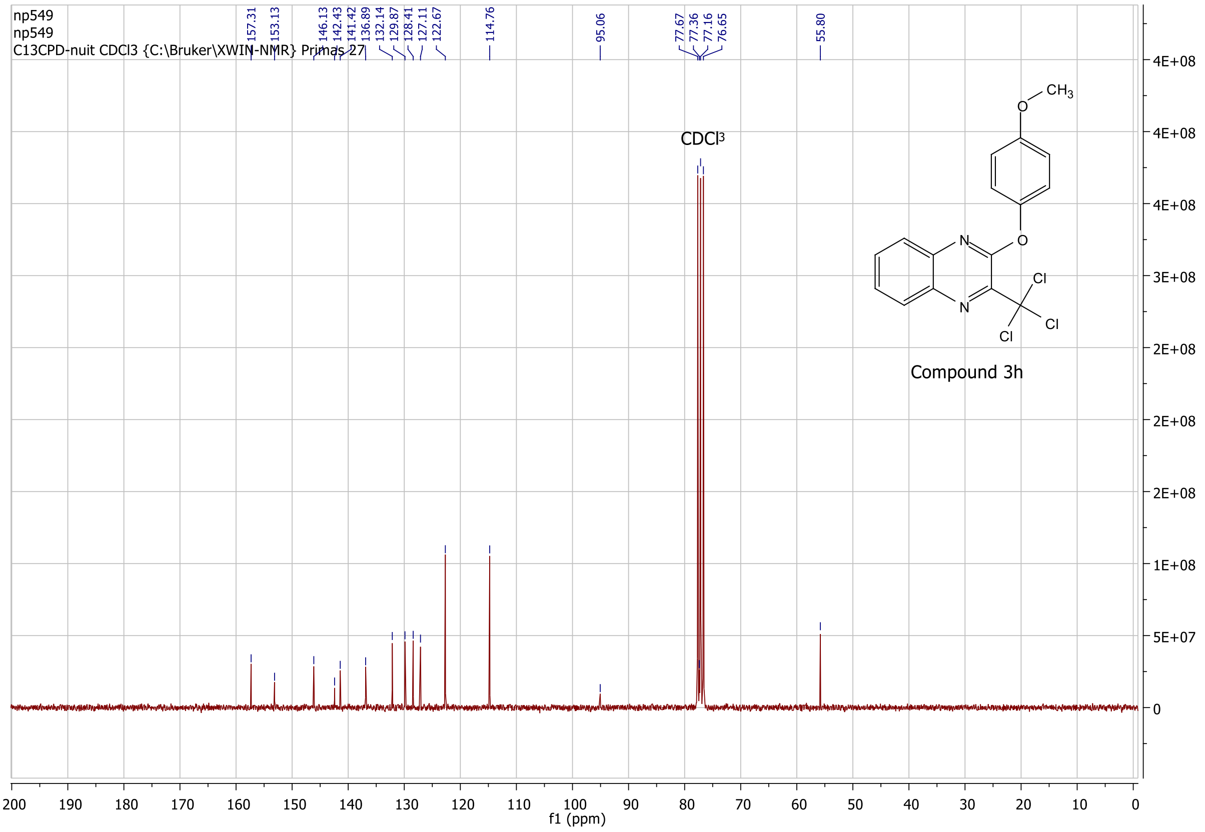

*Figure S16. ^{13}C NMR spectrum of **3h** in $CDCl_3$, on a Bruker ARX 63 spectrometer*

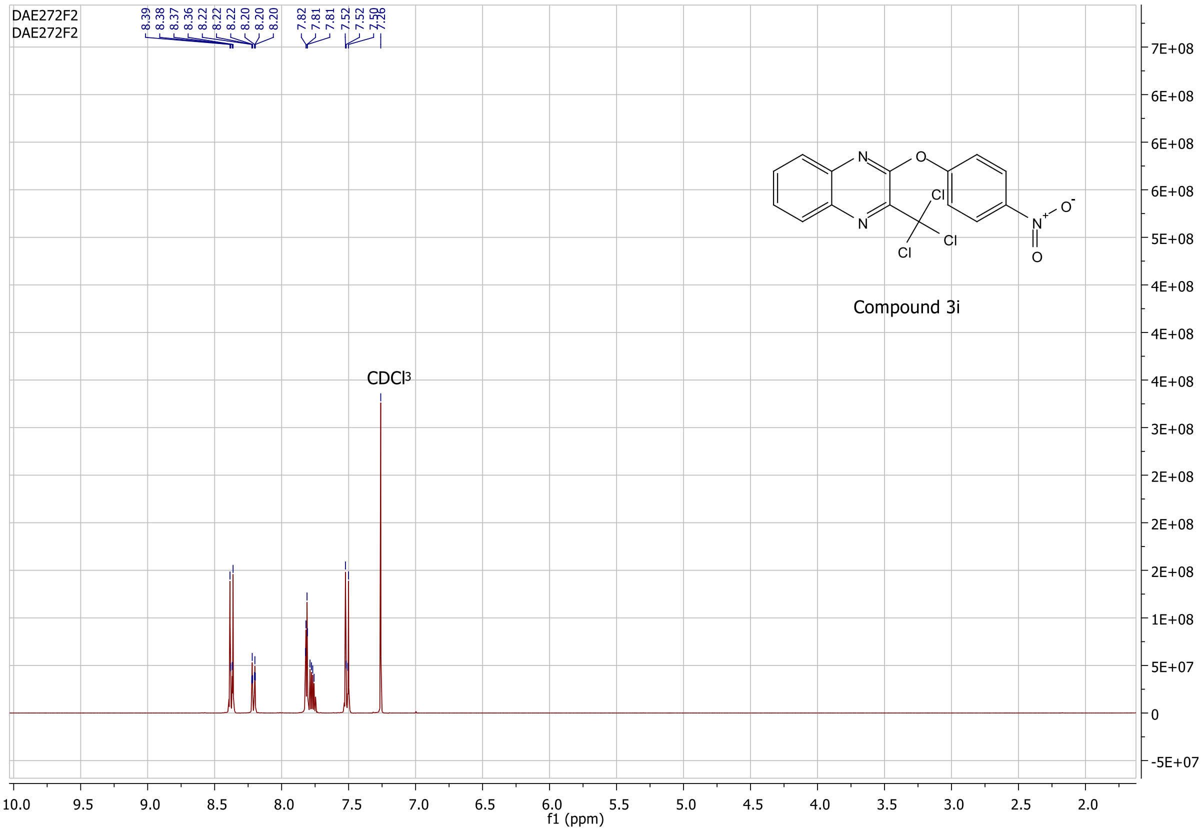

*Figure S17. ^{1}H NMR spectrum of **3i** in $CDCl_3$, on a Bruker ARX 400 spectrometer*

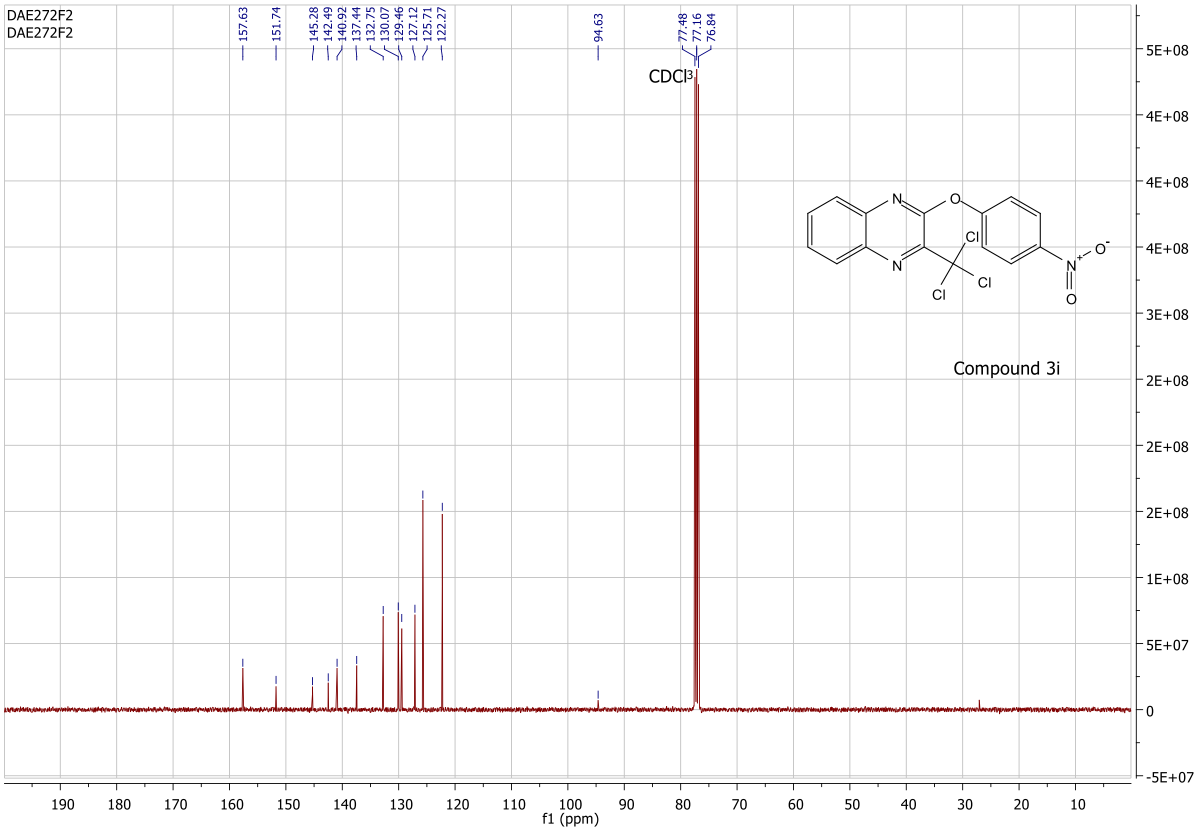

*Figure S18. ^{13}C NMR spectrum of **3i** in $CDCl_3$, on a Bruker ARX 101 spectrometer*

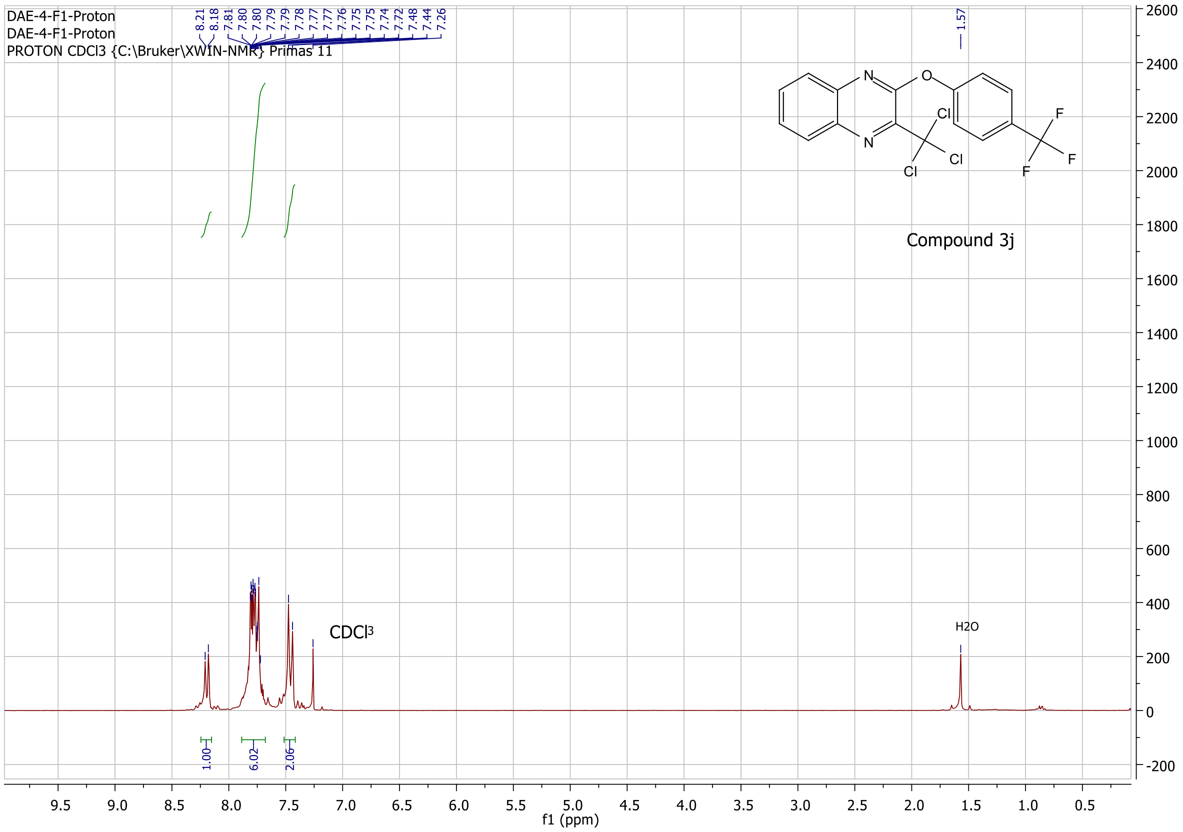

*Figure S19. ^{1}H NMR spectrum of **3j in** $CDCl_3$, on a Bruker ARX 250 spectrometer*

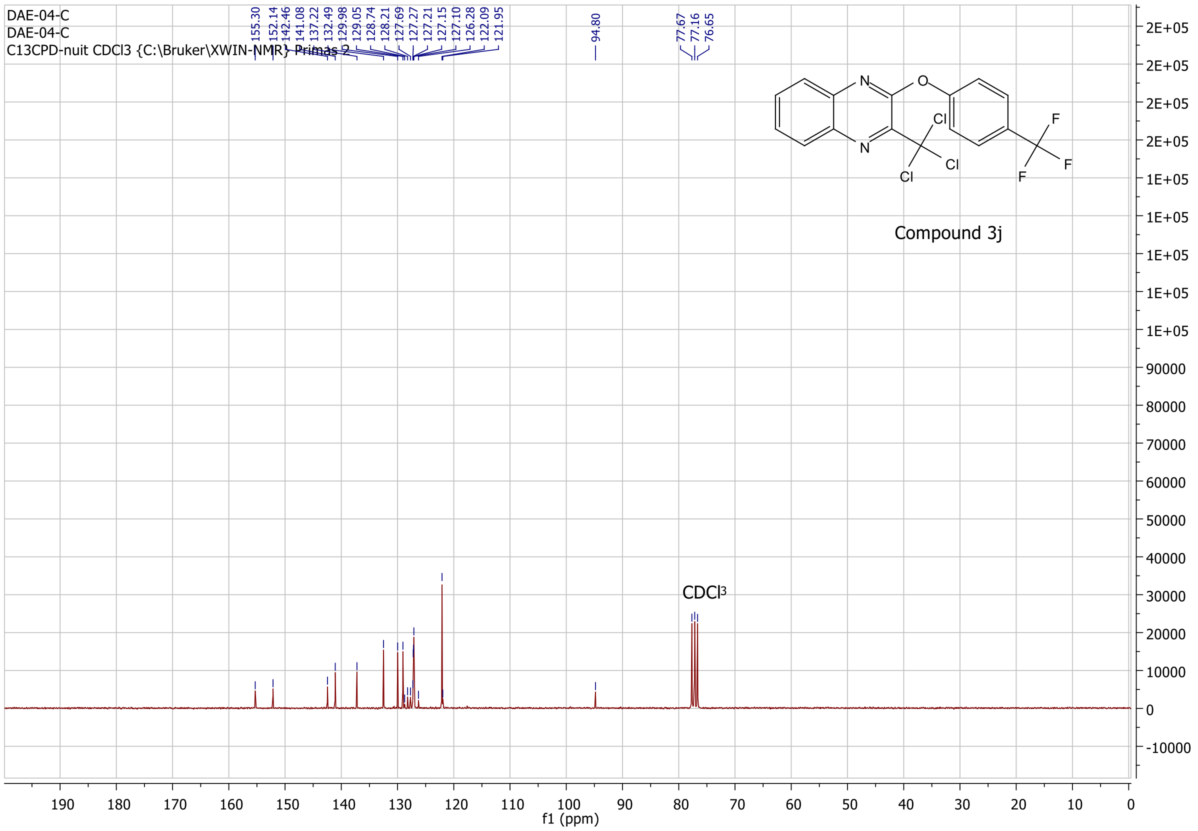

*Figure S20. ^{13}C NMR spectrum of **3j** in $CDCl_3$, on a Bruker ARX 63 spectrometer*

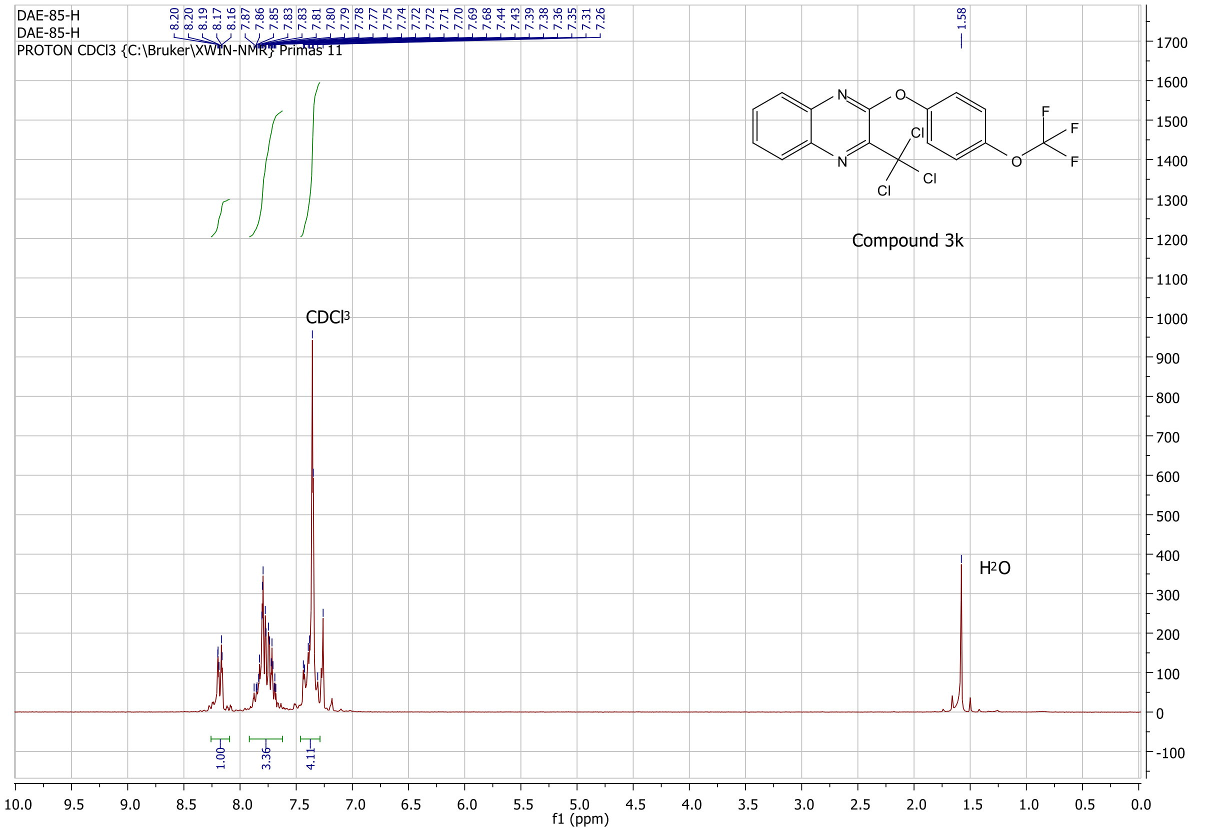

*Figure S21. ^{1}H NMR spectrum of **3k** in $CDCl_3$, on a Bruker ARX 250 spectrometer*

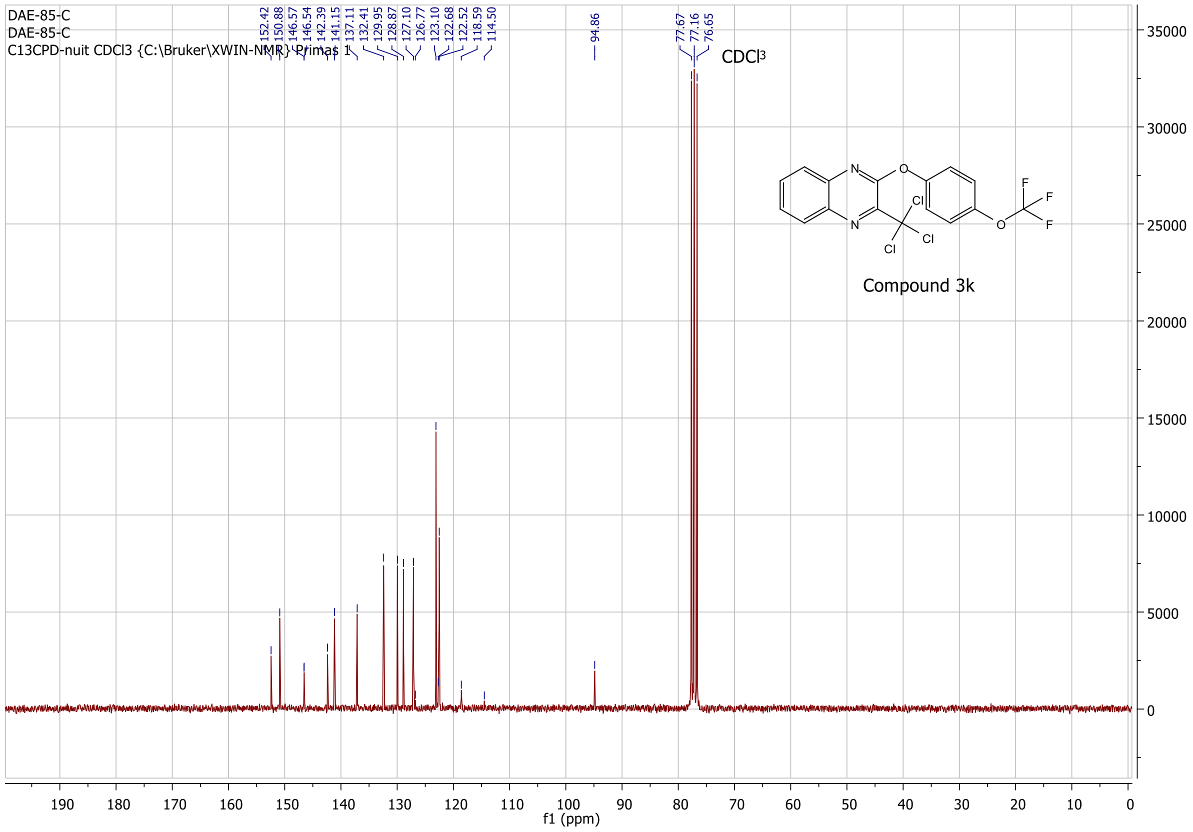

*Figure S22. ^{13}C NMR spectrum of **3k** in $CDCl_3$, on a Bruker ARX 63 spectrometer*

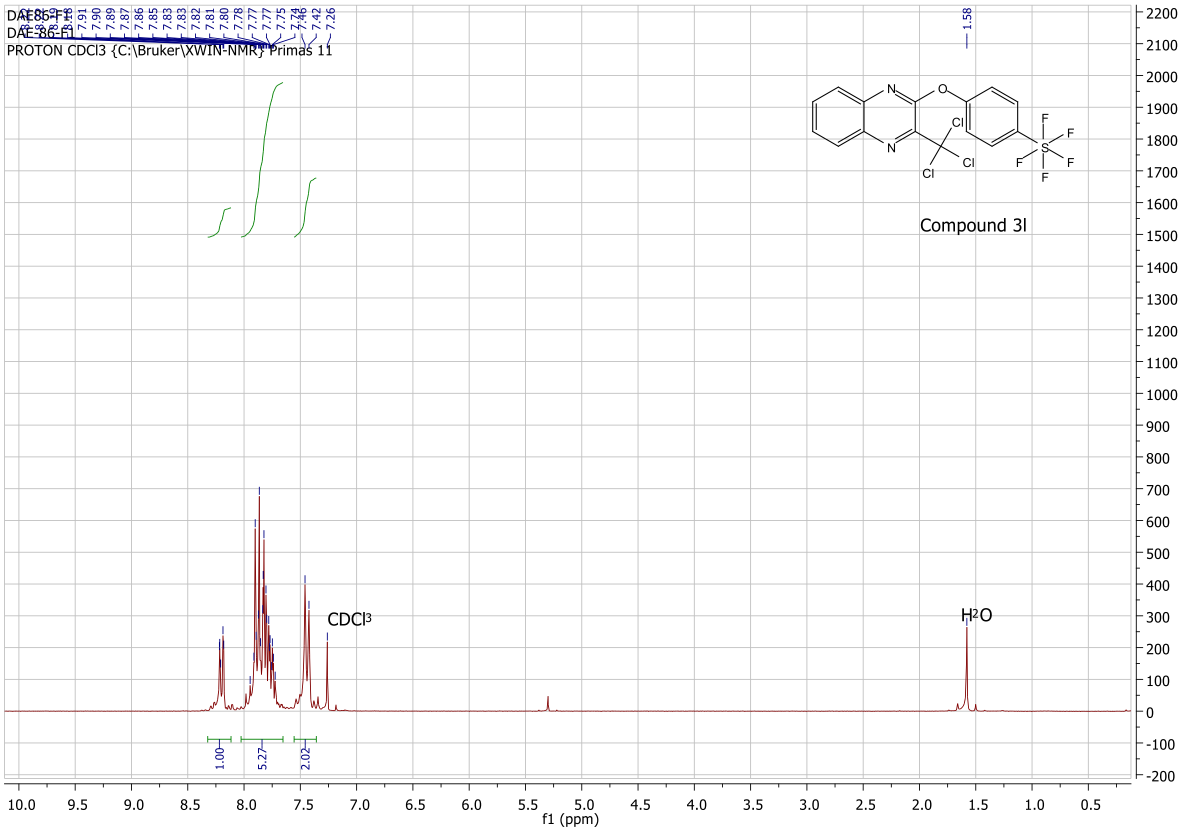

*Figure S23. ^{1}H NMR spectrum of **3l** in $CDCl_3$, on a Bruker ARX 250 spectrometer*

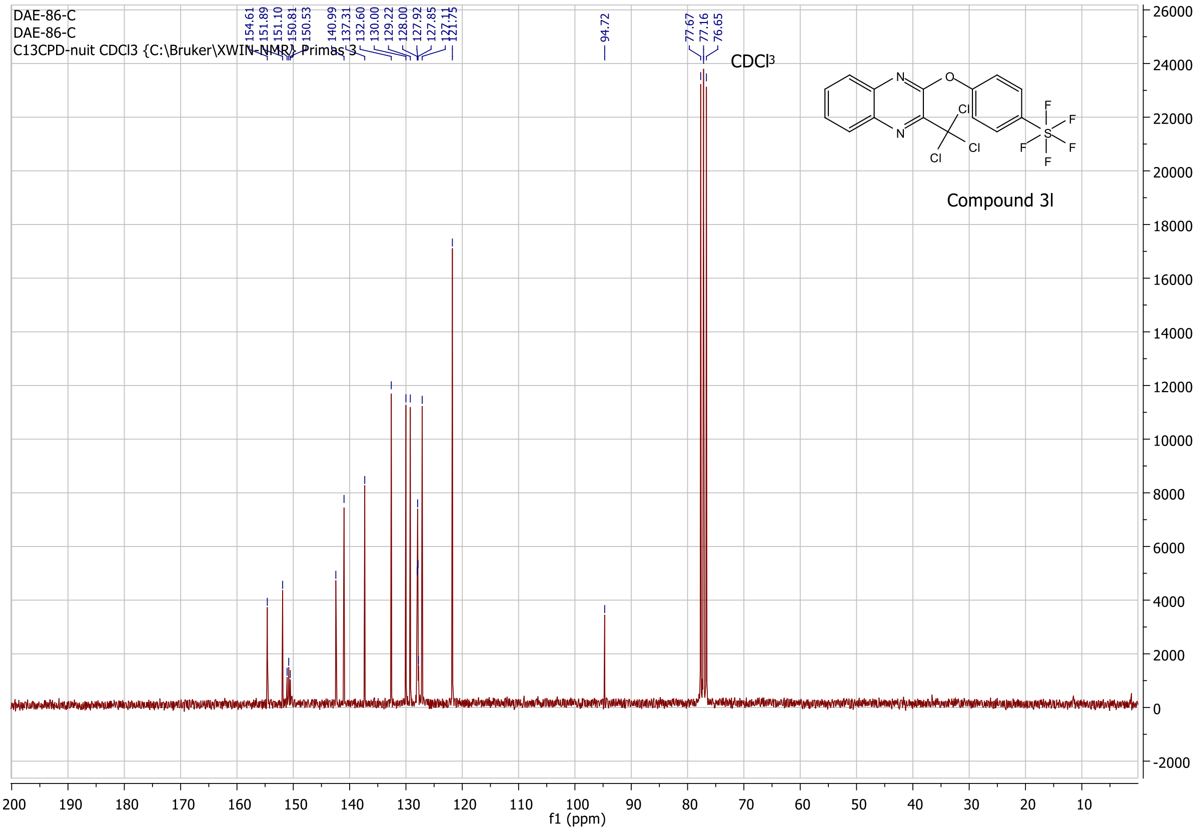

*Figure S24. ^{13}C NMR spectrum of **3l** in $CDCl_3$, on a Bruker ARX 63 spectrometer*

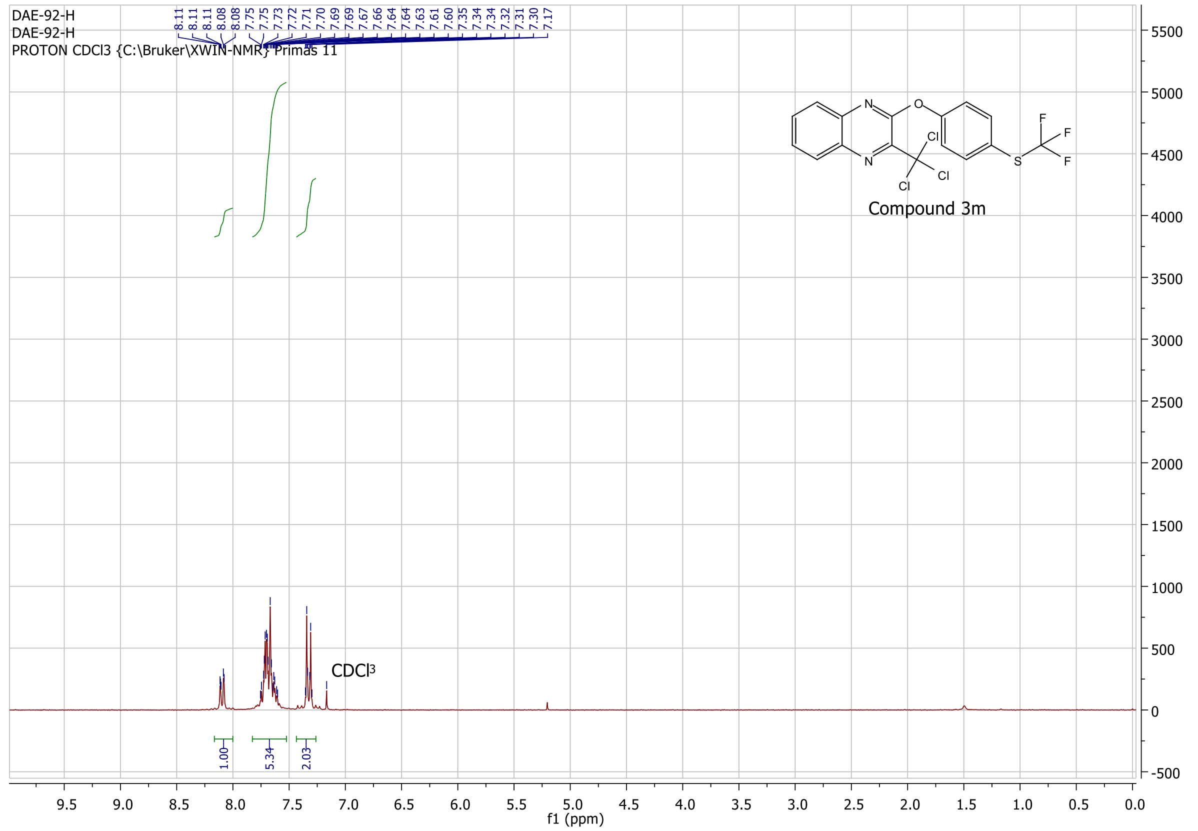

*Figure S25. ^{1}H NMR spectrum of **3m** in $CDCl_3$, on a Bruker ARX 250 spectrometer*

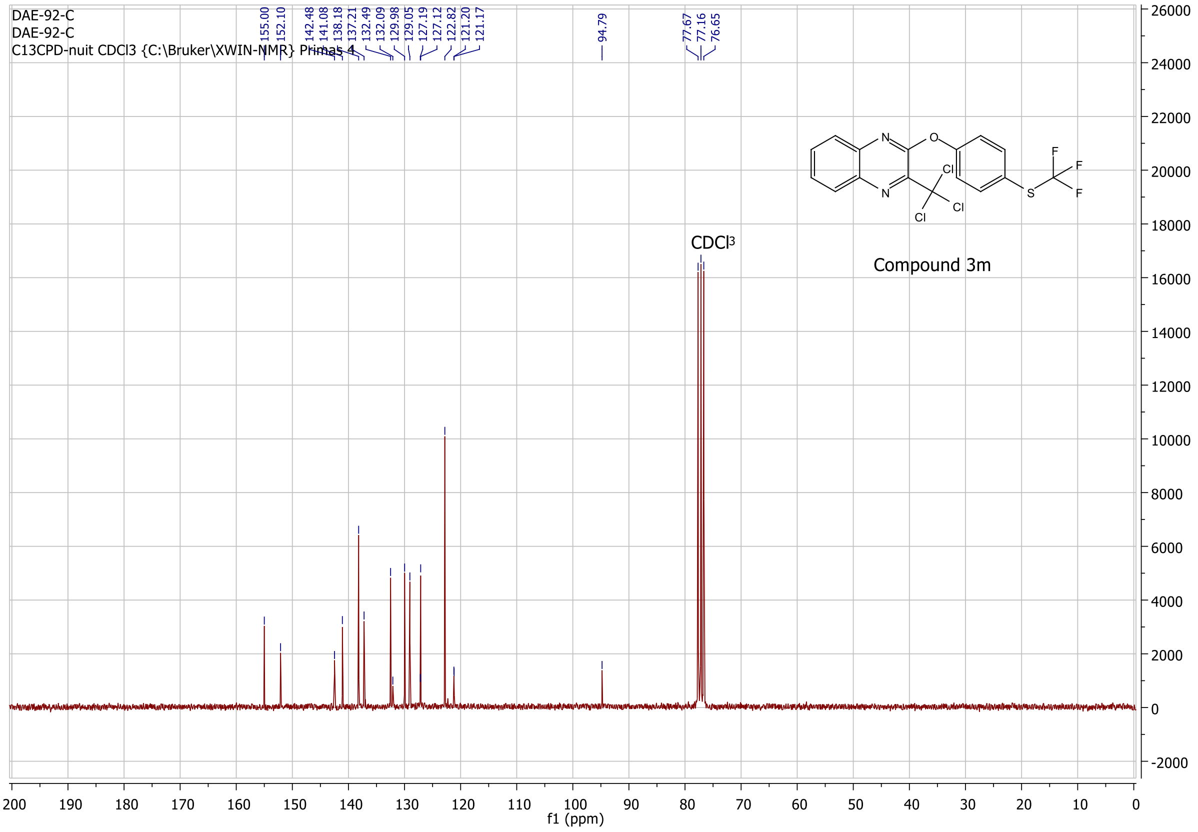

*Figure S26. ^{13}C NMR spectrum of **3m** in $CDCl_3$, on a Bruker ARX 63 spectrometer*

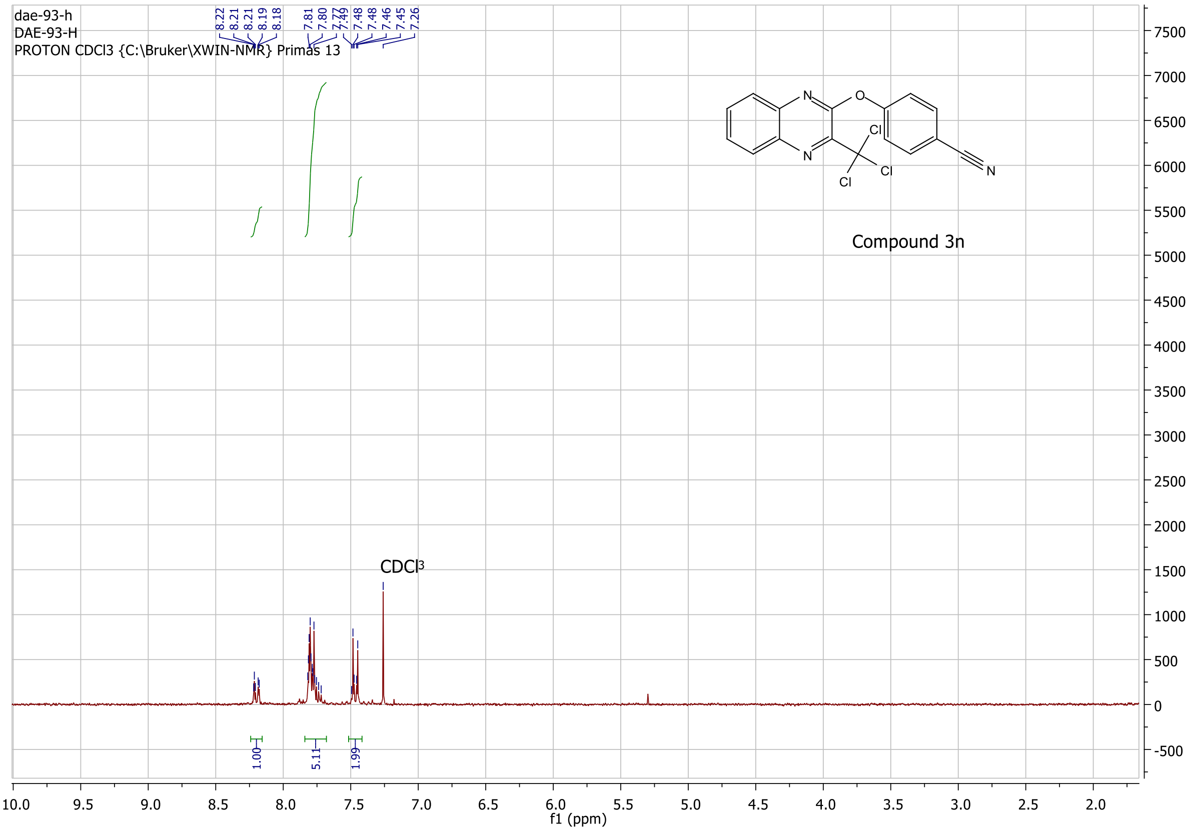

*Figure S27. ^{1}H NMR spectrum of **3n** in $CDCl_3$, on a Bruker ARX 250 spectrometer*

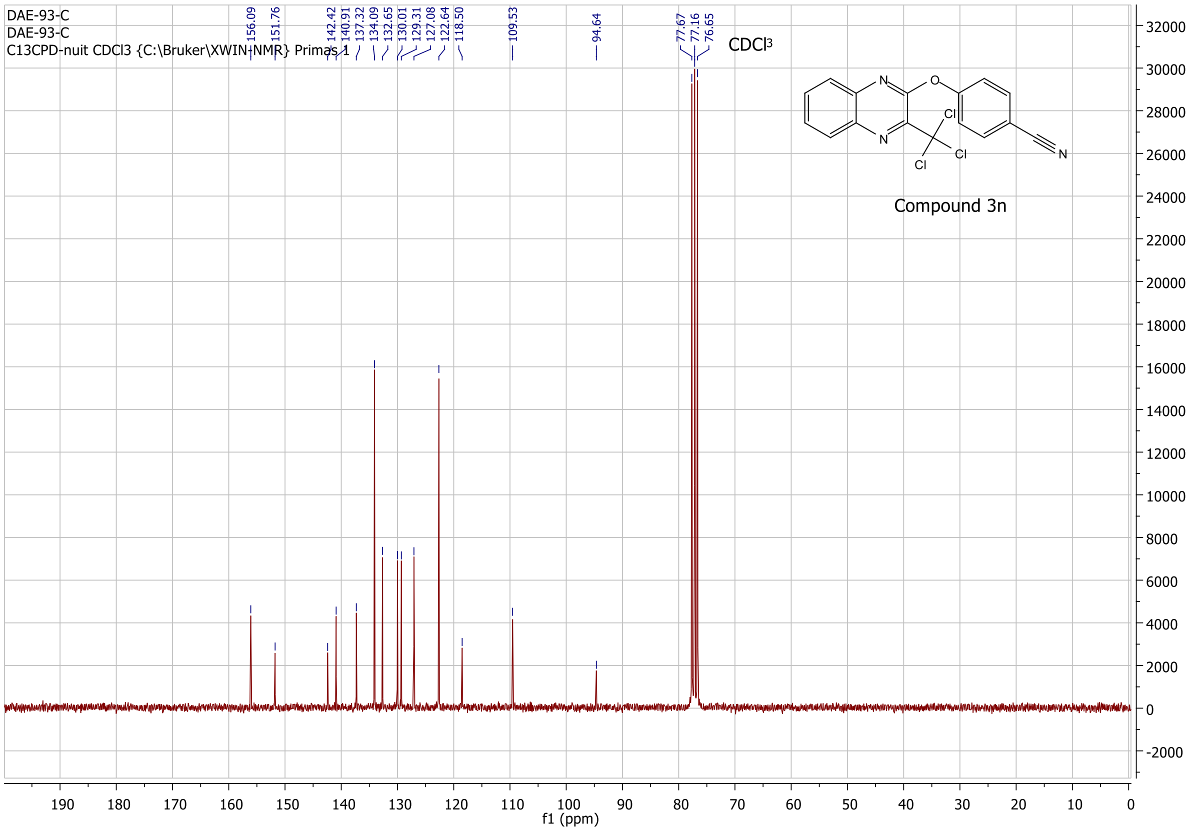

*Figure S28. ^{13}C NMR spectrum of **3n** in $CDCl_3$, on a Bruker ARX 63 spectrometer*

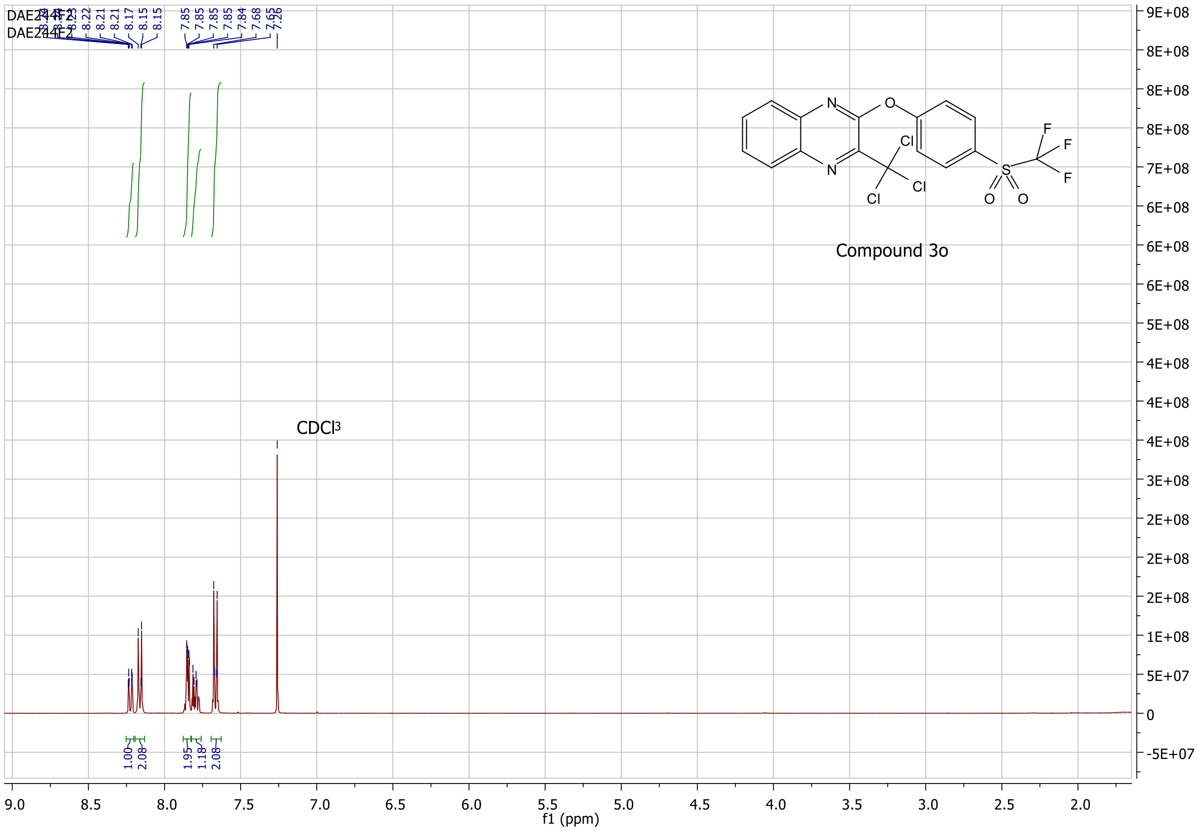

*Figure S29. ^{1}H NMR spectrum of **3o** in $CDCl_3$, on a Bruker ARX 250 spectrometer*

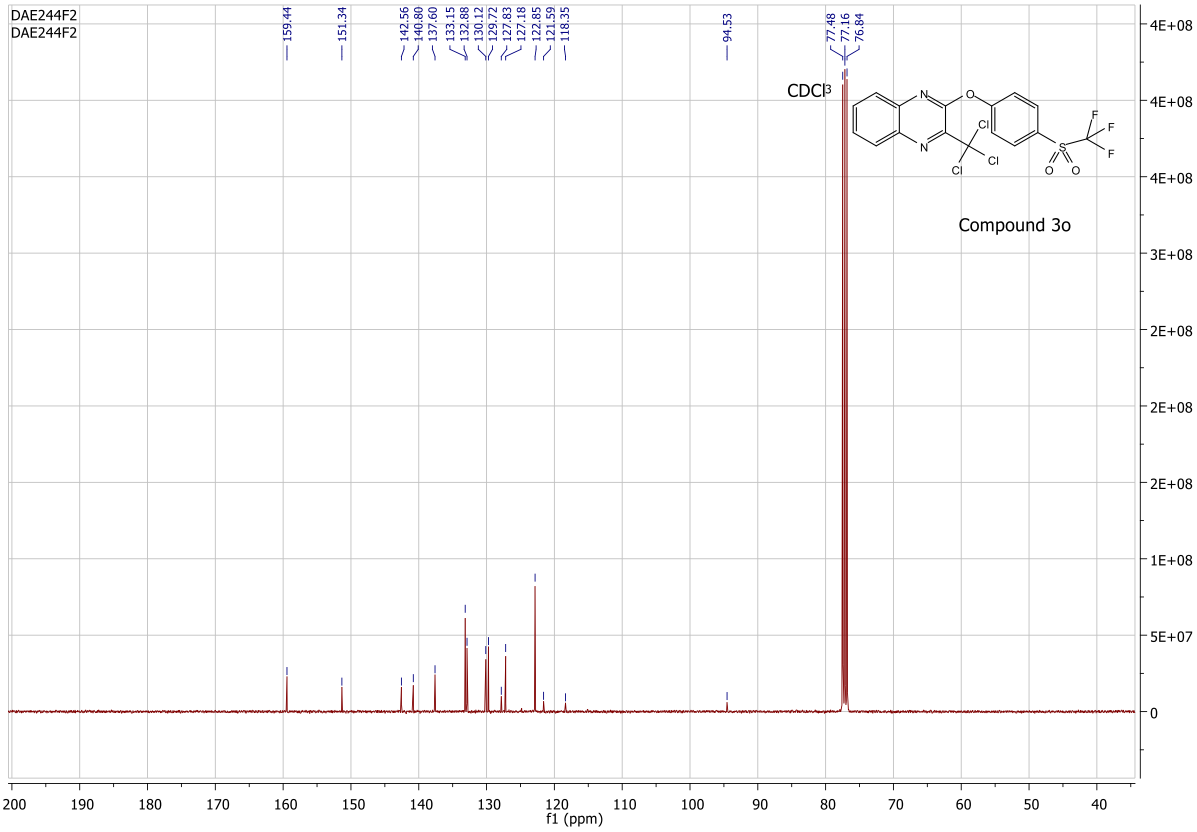

*Figure S30. ^{13}C NMR spectrum of **3o** in $CDCl_3$, on a Bruker ARX 63 spectrometer*

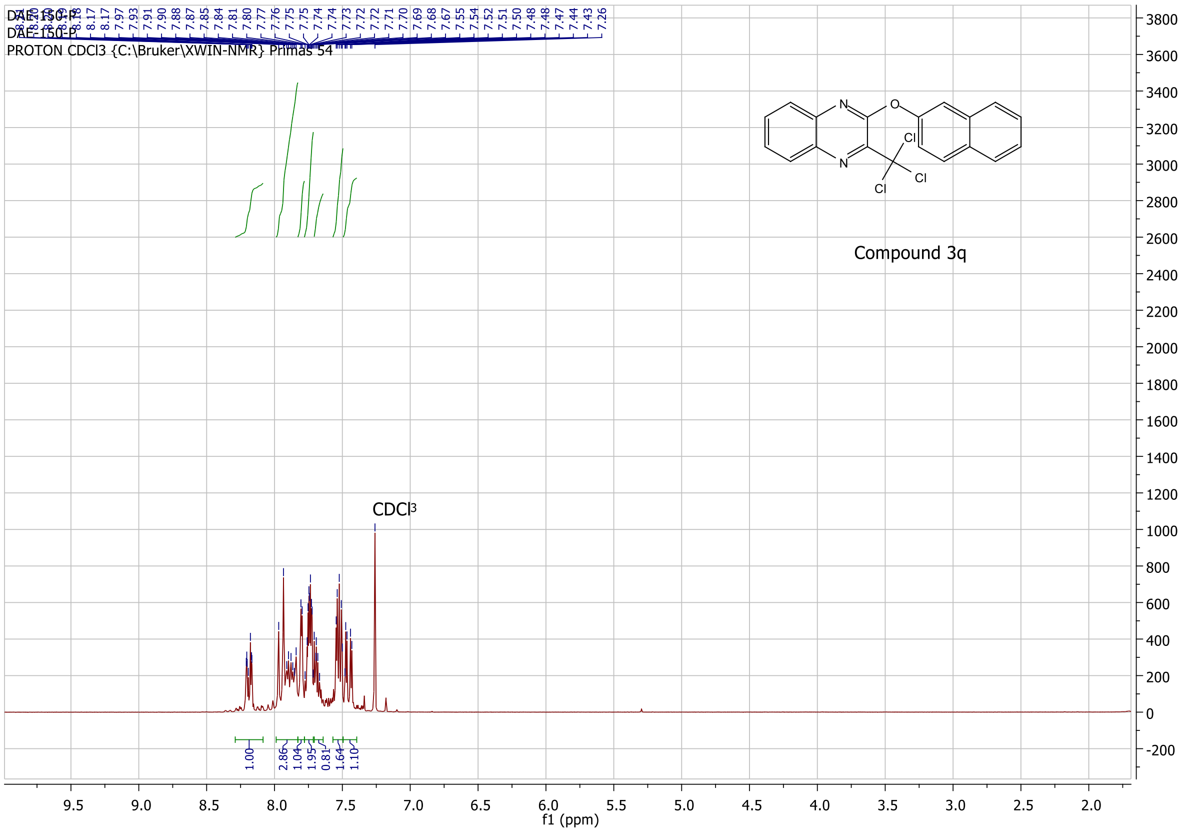

*Figure S31. ^{1}H NMR spectrum of **3q** in $CDCl_3$, on a Bruker ARX 250 spectrometer*

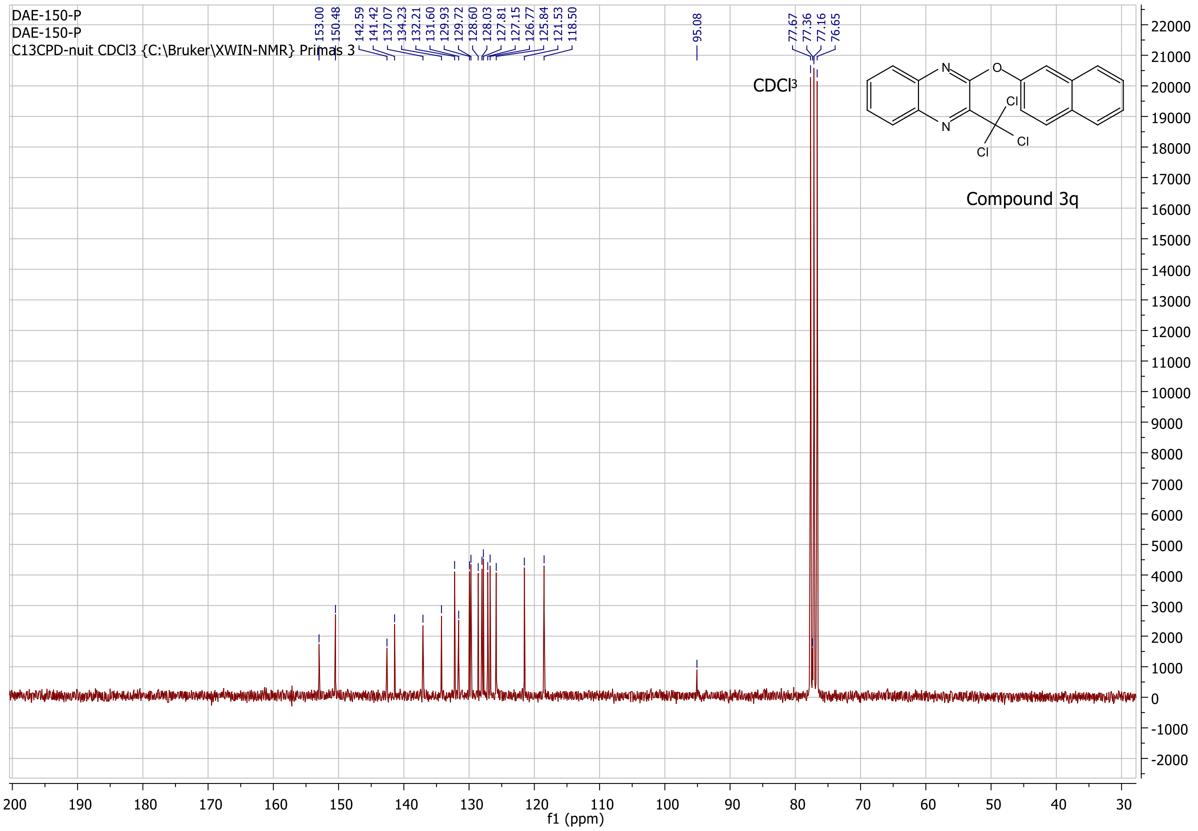

*Figure S32. ^{13}C NMR spectrum of **3q** in $CDCl_3$, on a Bruker ARX 63 spectrometer*

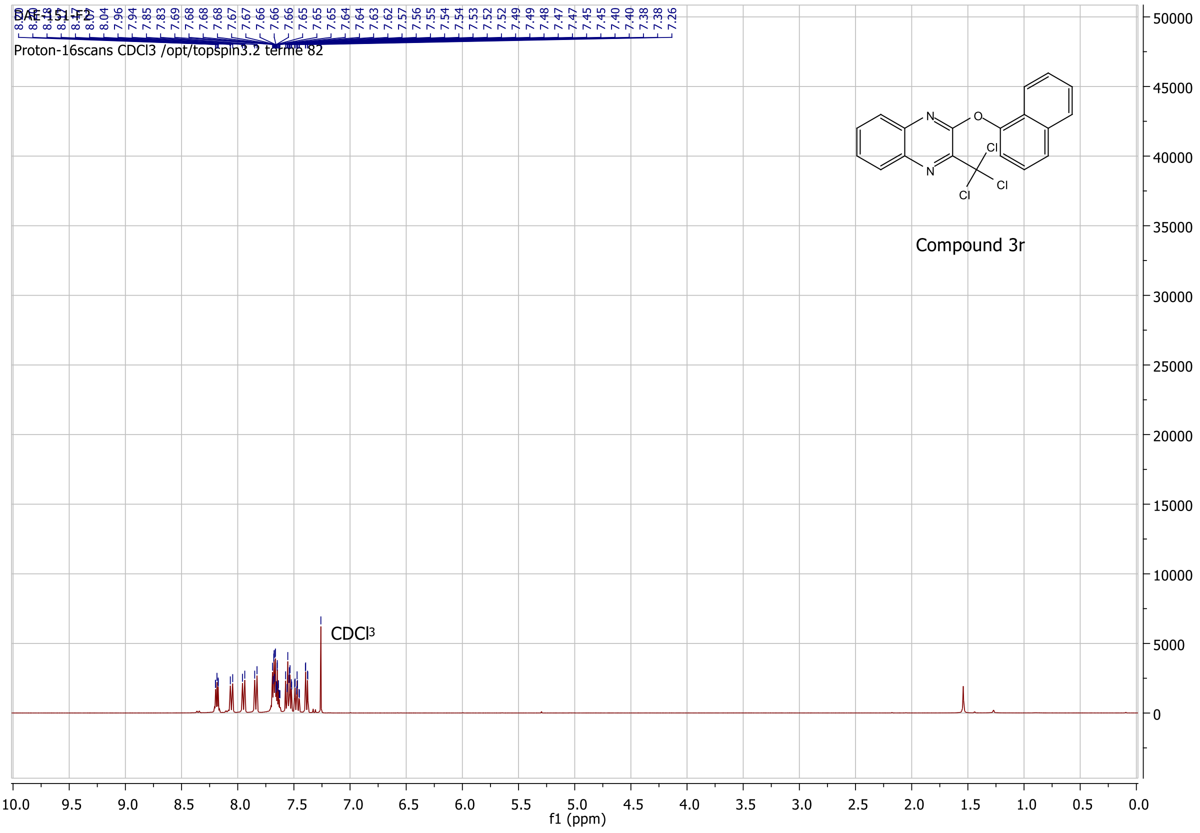

*Figure S33. ^{1}H NMR spectrum of **3r** in $CDCl_3$, on a Bruker ARX 250 spectrometer*

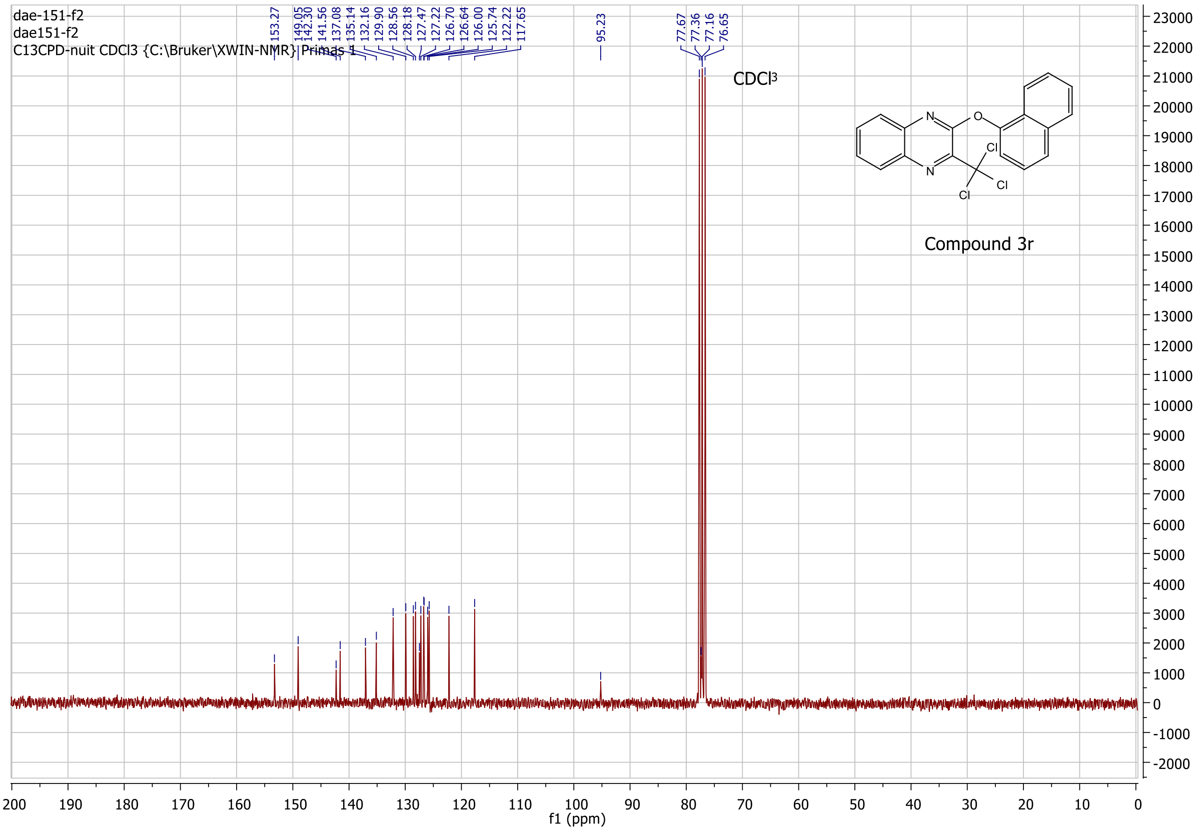

*Figure S34. ^{13}C NMR spectrum of **3r** in $CDCl_3$, on a Bruker ARX 63 spectrometer*

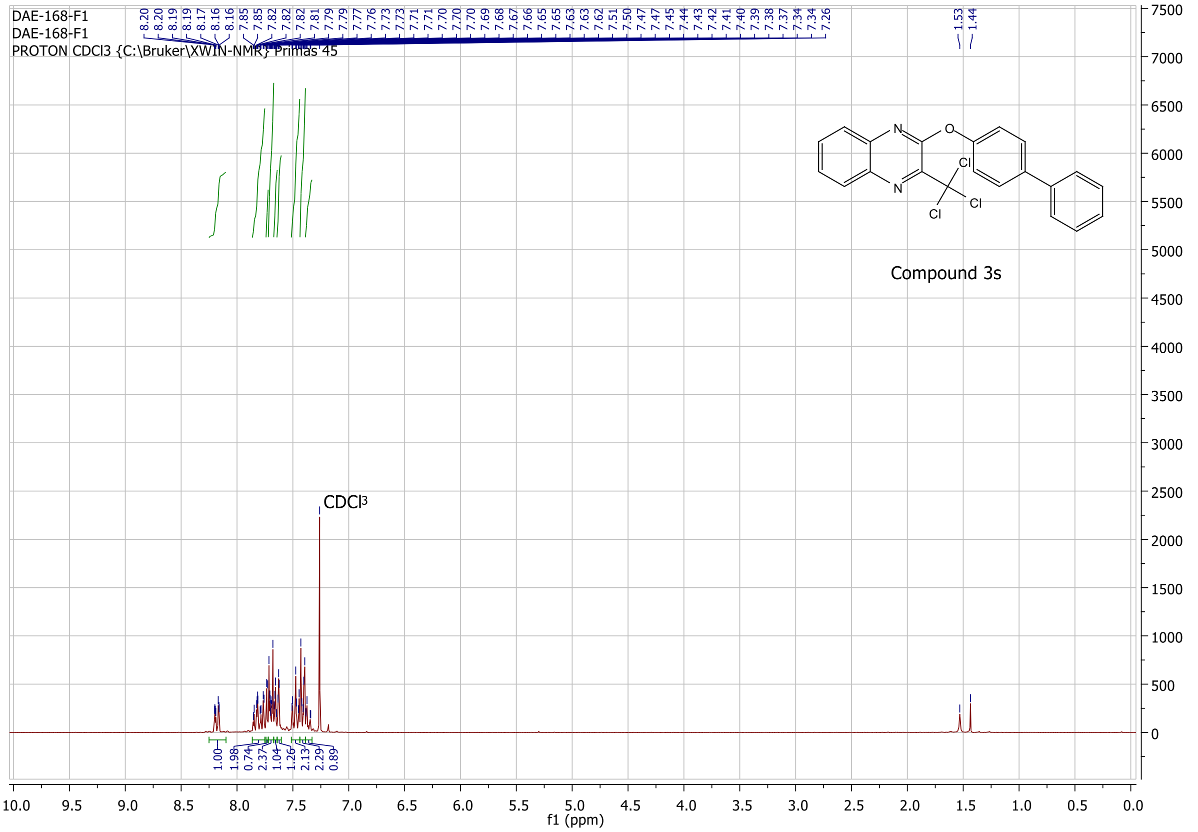

*Figure S35. ^{1}H NMR spectrum of **3s** in $CDCl_3$, on a Bruker ARX 250 spectrometer*

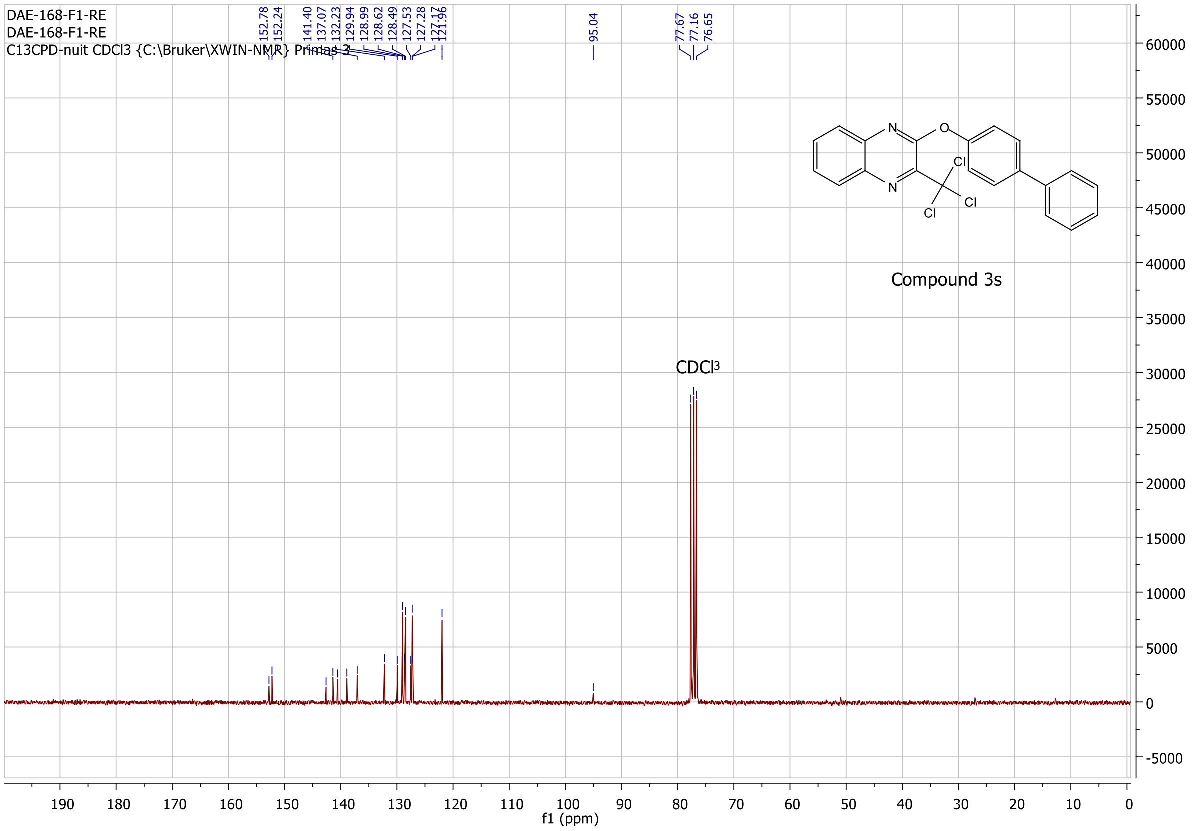

*Figure S36. ^{13}C NMR spectrum of **3s** in $CDCl_3$, on a Bruker ARX 63 spectrometer*

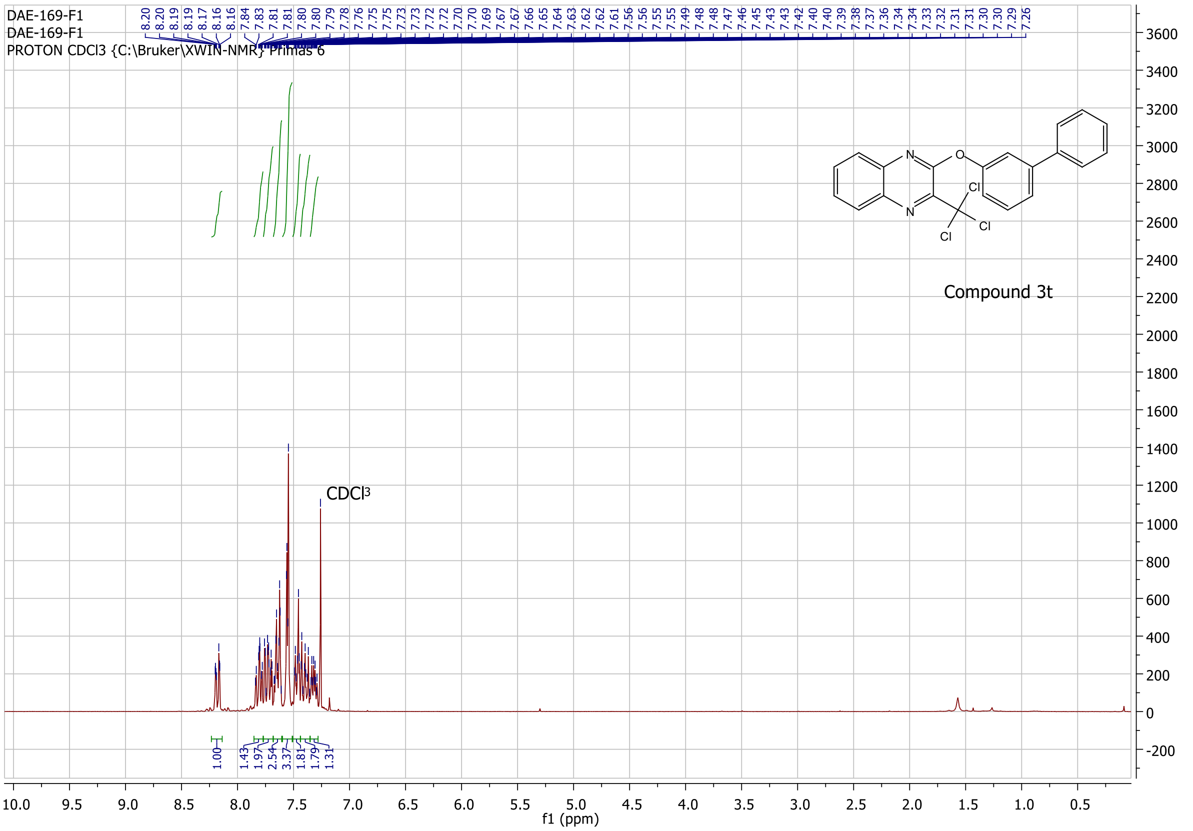

*Figure S37. ^{1}H NMR spectrum of **3t** in $CDCl_3$, on a Bruker ARX 250 spectrometer*

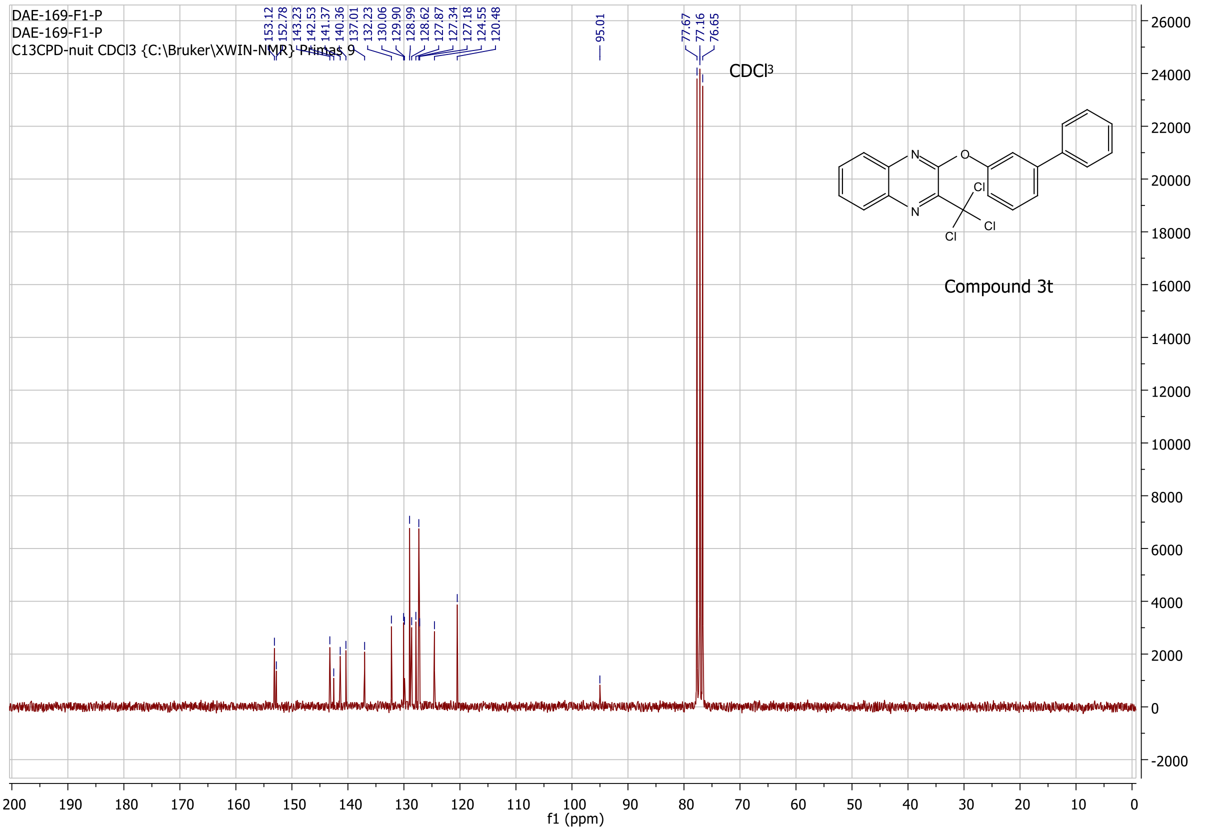

*Figure S38. ^{13}C NMR spectrum of **3t** in $CDCl_3$, on a Bruker ARX 63 spectrometer*

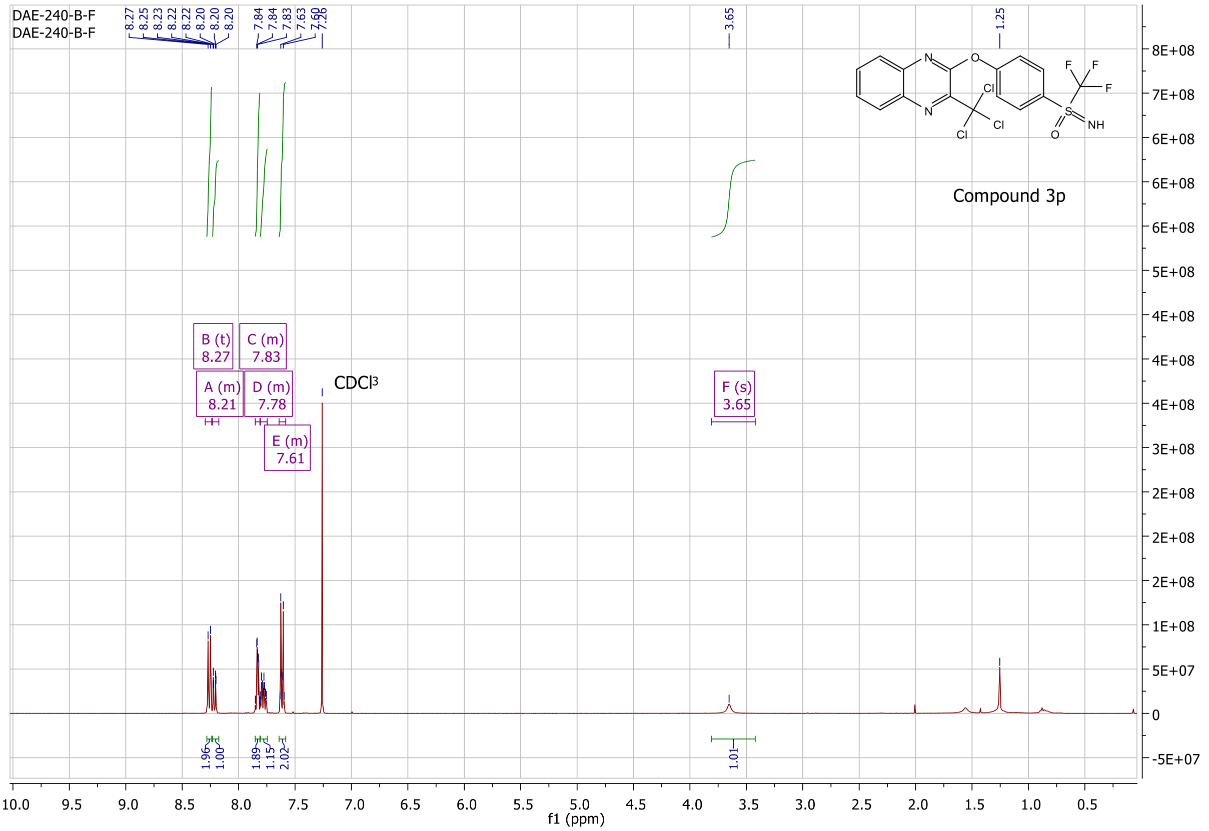

*Figure S39. ^{1}H NMR spectrum of **3p** in $CDCl_3$, on a Bruker ARX 250 spectrometer*

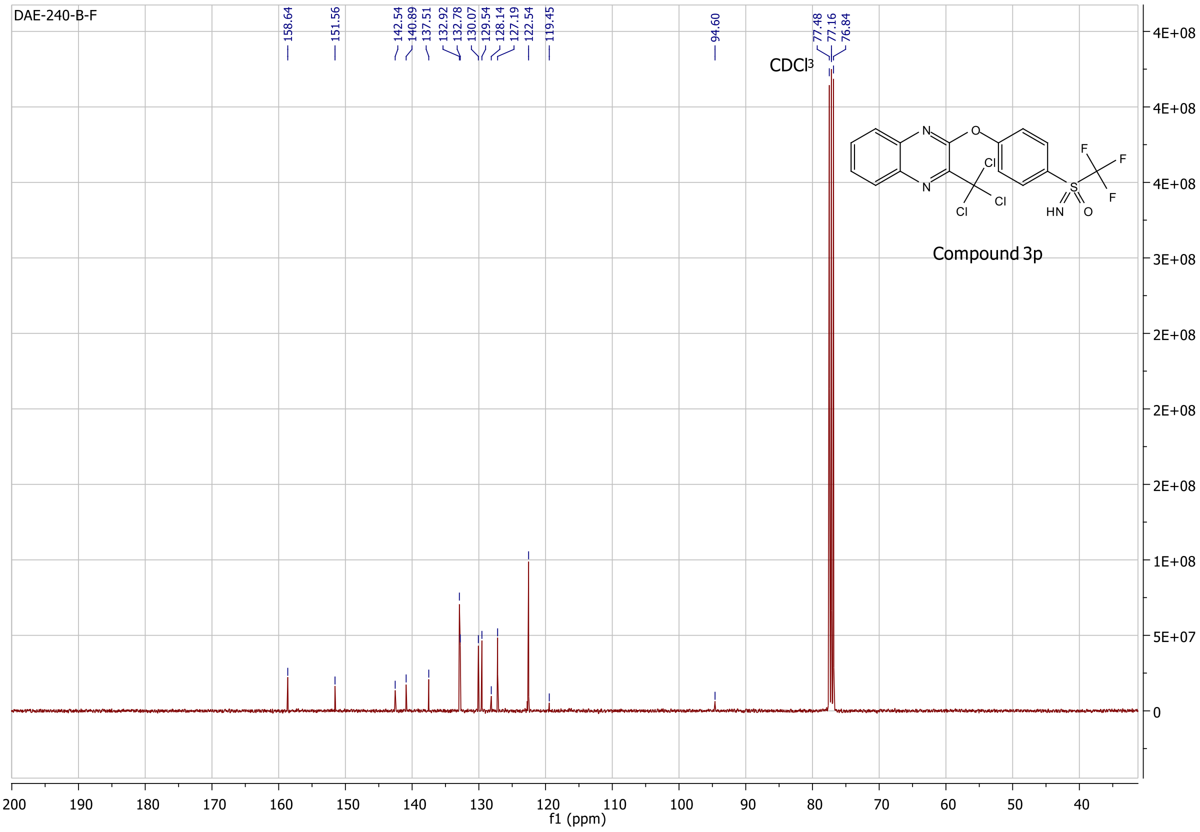

*Figure S40. ^{13}C NMR spectrum of **3p** in $CDCl_3$, on a Bruker ARX 63 spectrometer*

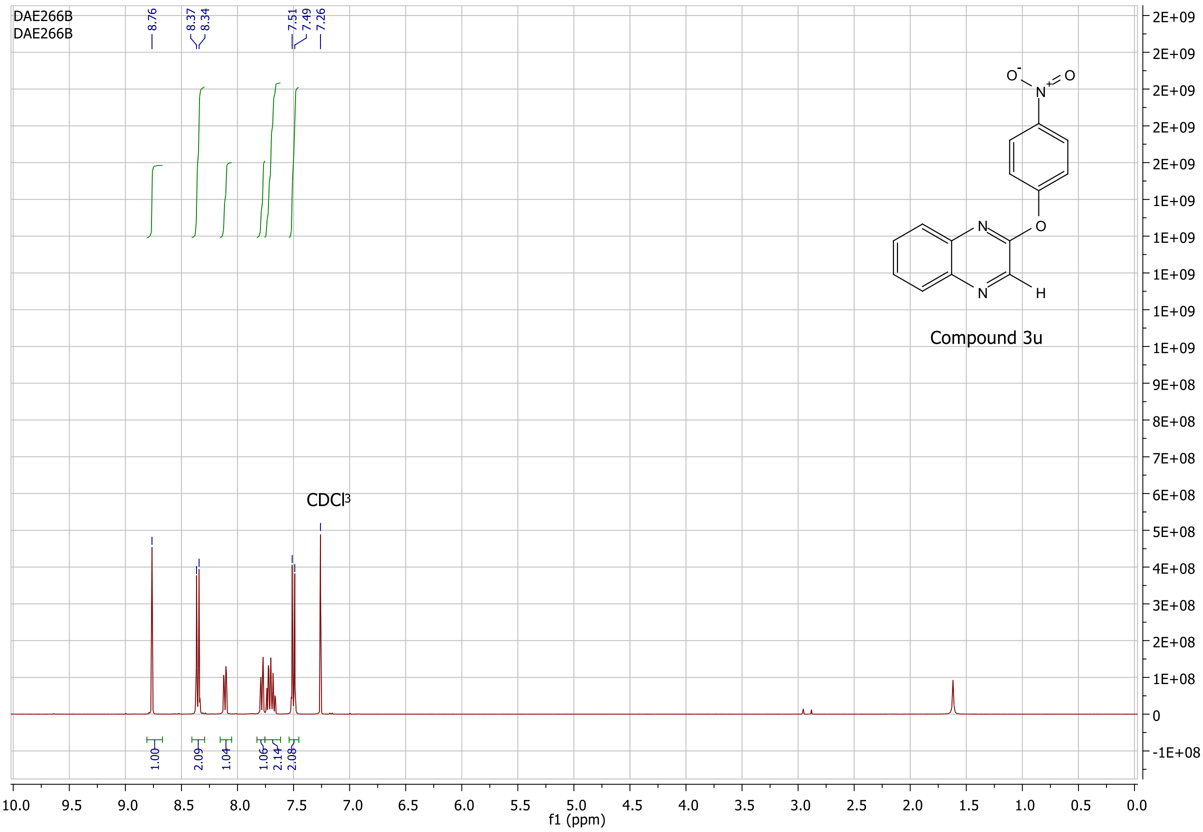

*Figure S41. ^{1}H NMR spectrum of **3u** in $CDCl_3$, on a Bruker ARX 250 spectrometer*

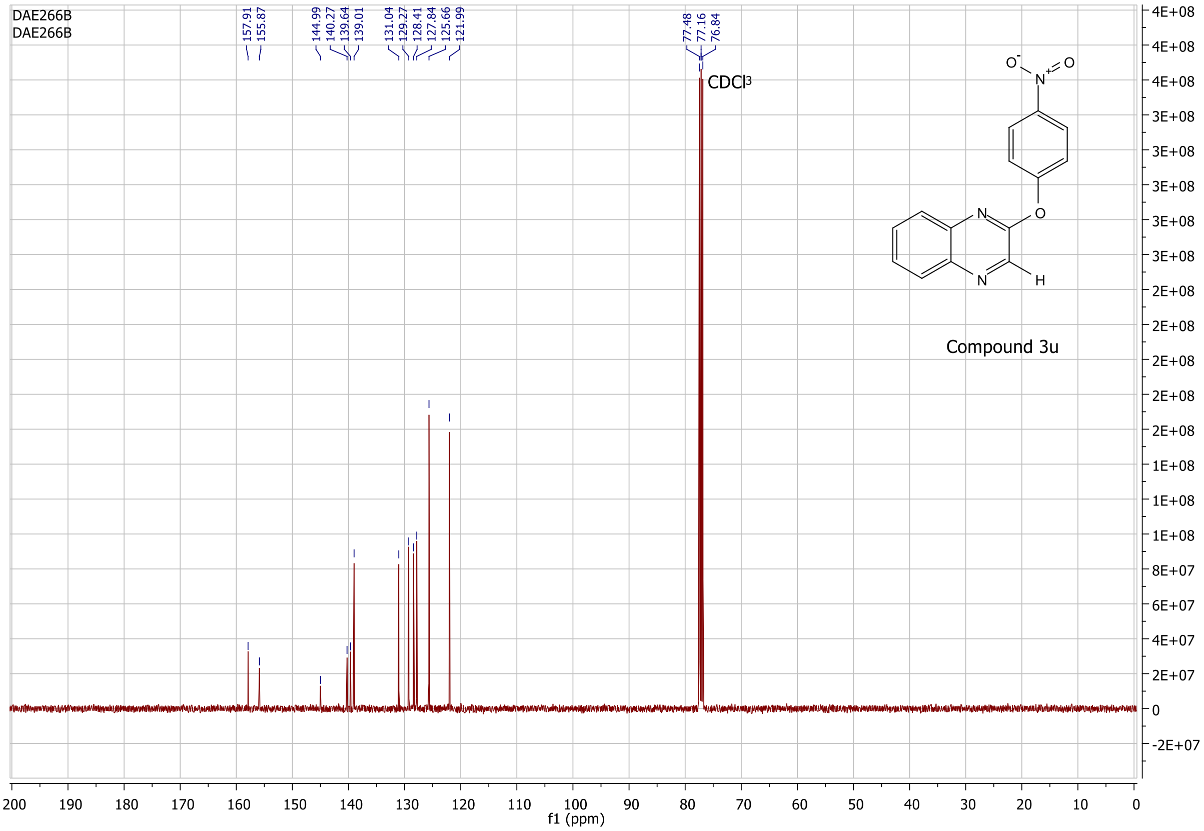

*Figure S42. ^{13}C NMR spectrum of **3u** in $CDCl_3$, on a Bruker ARX 63 spectrometer*

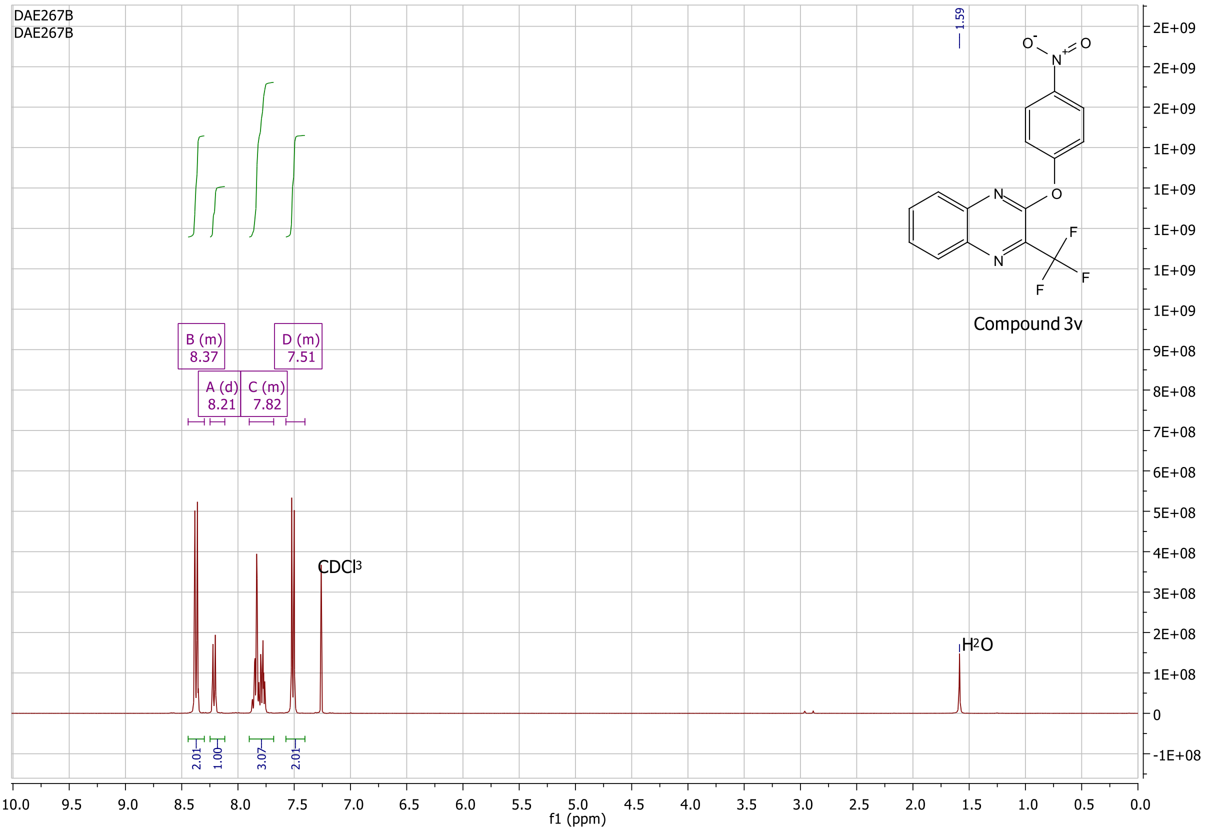

*Figure S43. ^{1}H NMR spectrum of **3v** in $CDCl_3$, on a Bruker ARX 250 spectrometer*

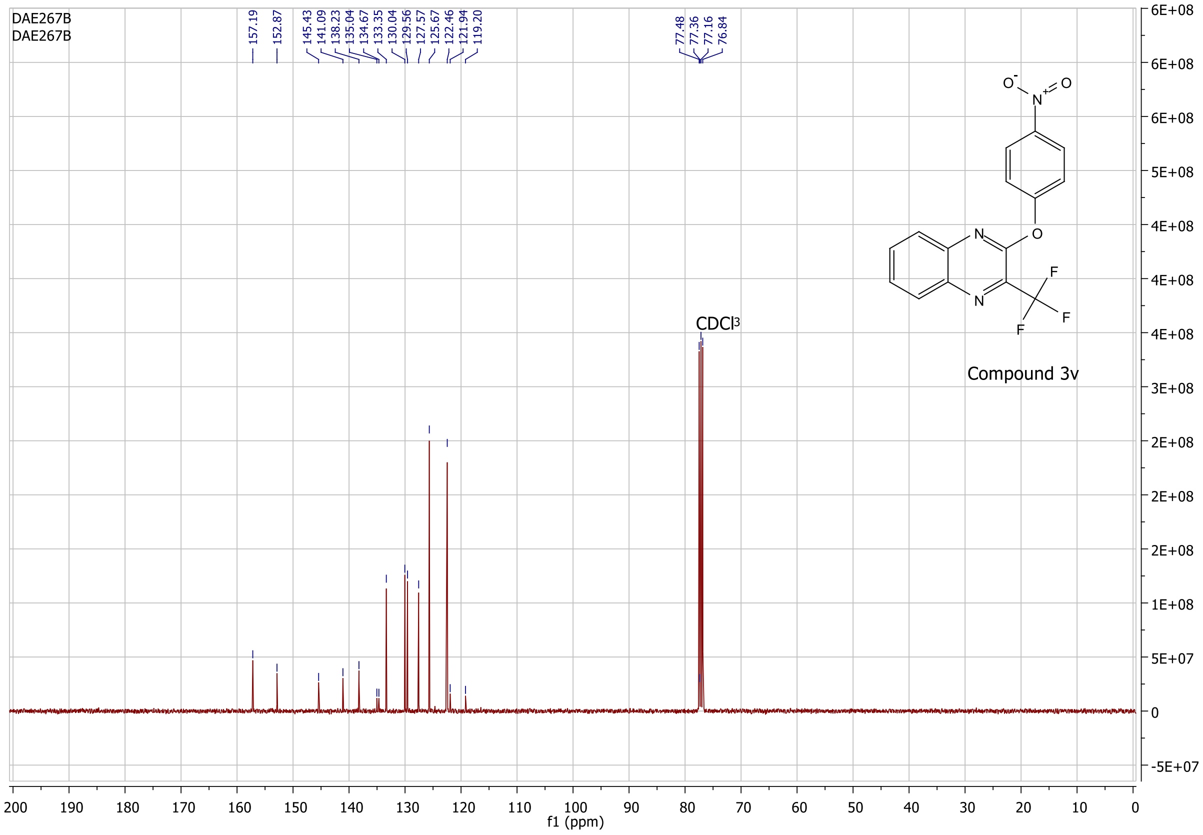

*Figure S44. ^{13}C NMR spectrum of **3v** in $CDCl_3$, on a Bruker ARX 63 spectrometer*

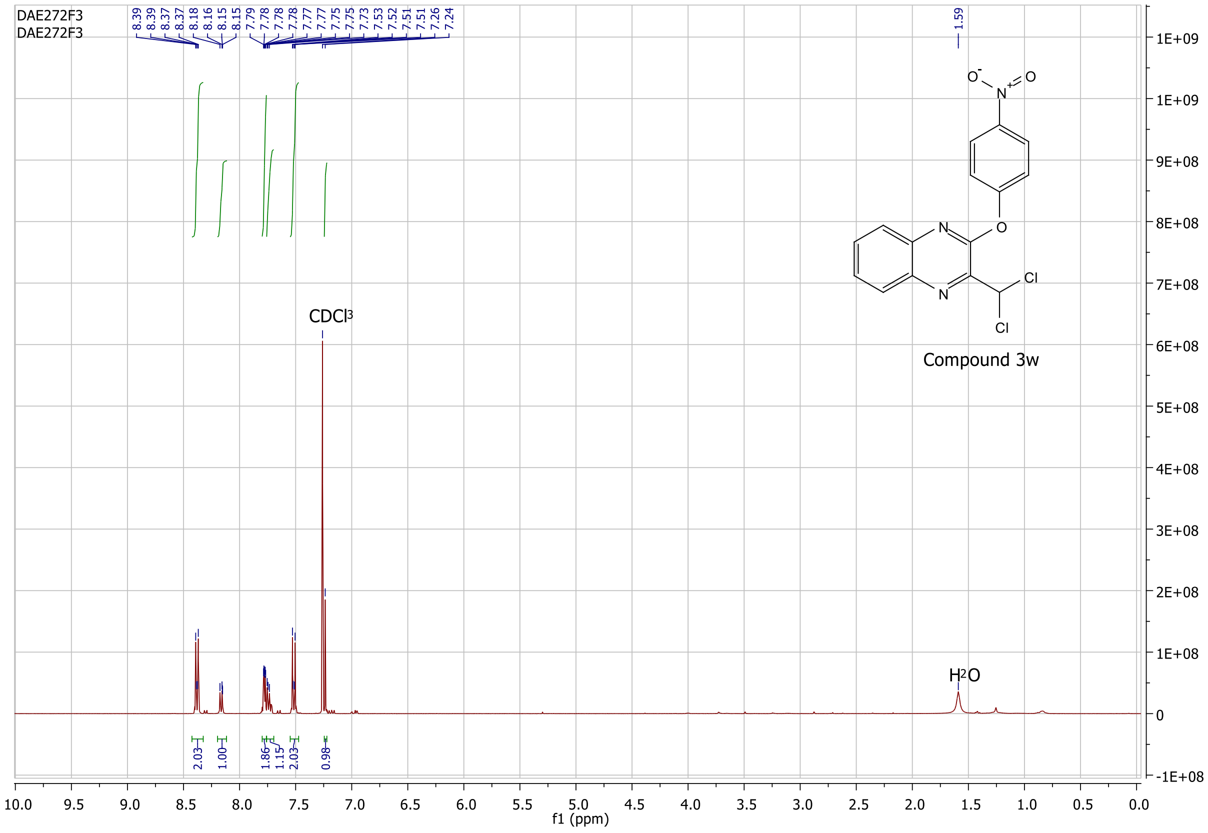

*Figure S45. ^{1}H NMR spectrum of **3w** in $CDCl_3$, on a Bruker ARX 250 spectrometer*

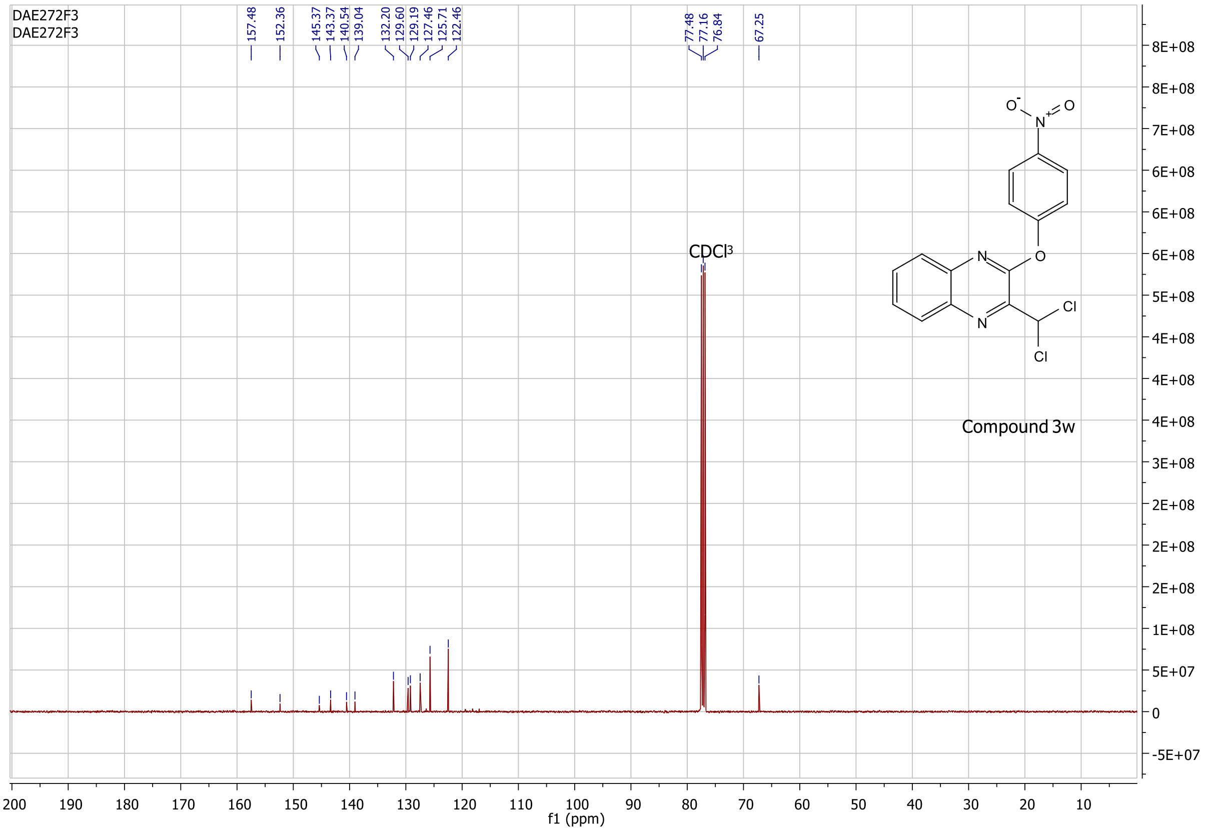

*Figure S46. ^{13}C NMR spectrum of **3w** in $CDCl_3$, on a Bruker ARX 63 spectrometer*

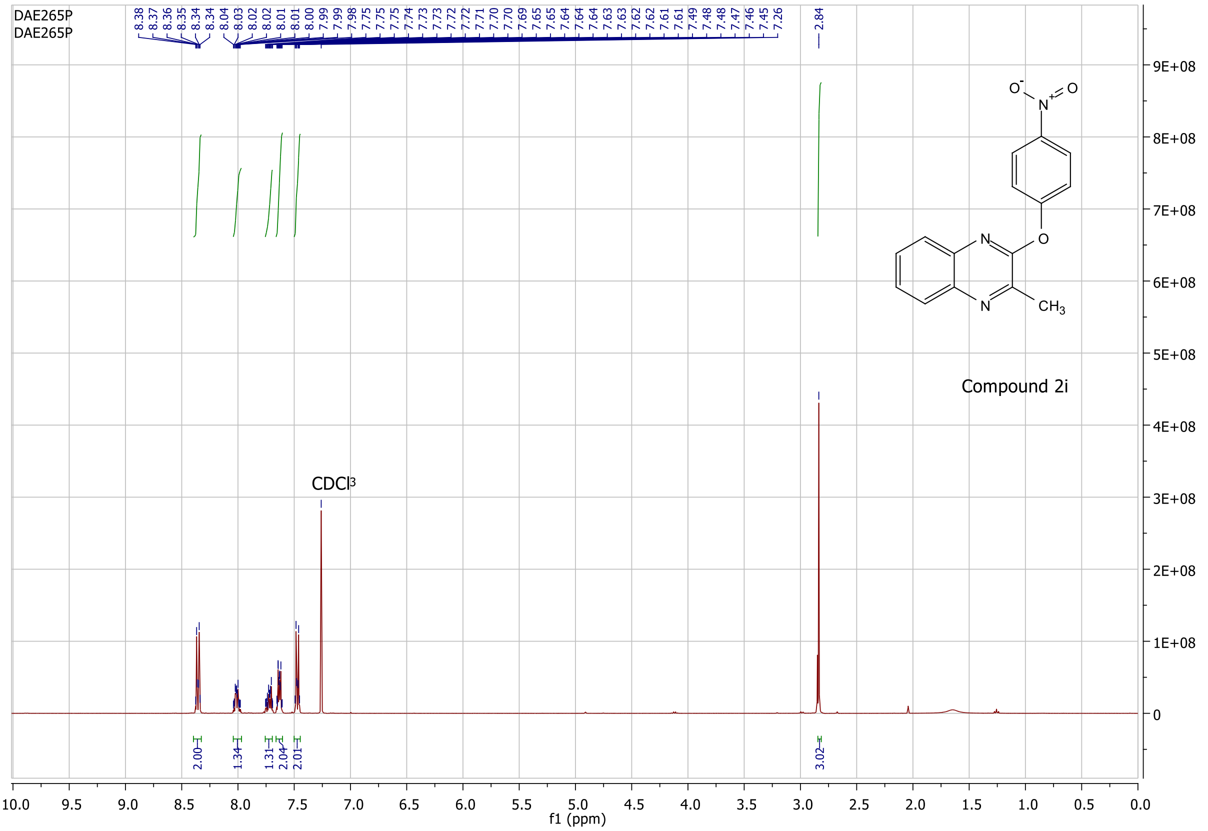

*Figure S47. ^{1}H NMR spectrum of **2i** in $CDCl_3$, on a Bruker ARX 250 spectrometer*

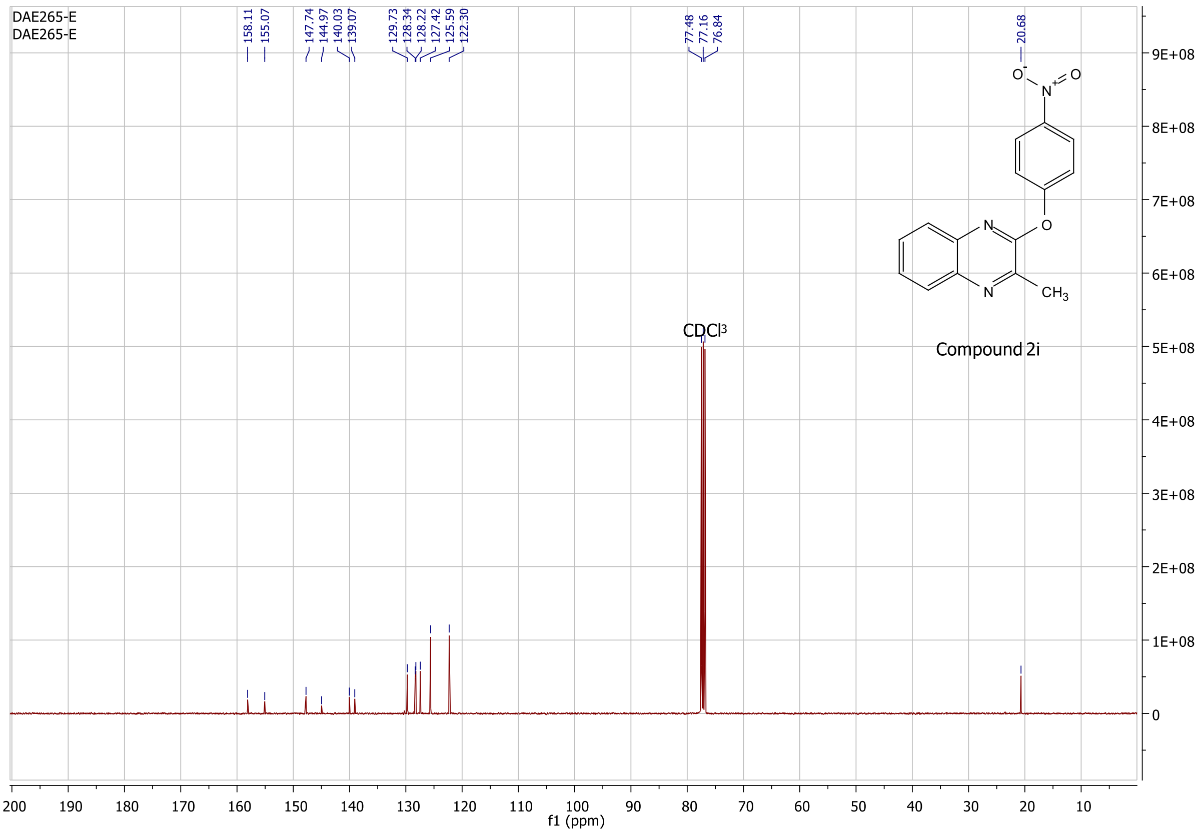

*Figure S48. ^{13}C NMR spectrum of **2i** in $CDCl_3$, on a Bruker ARX 101 spectrometer*

2. Evaluation on artemisinin-resistant and artemisinin-sensitive parasites

Artemisinin-resistance is demonstrated by the recrudescence assay with a significant faster recrudescence for F32-ART5 than for F32-TEM after 48 h ART exposure [33,34] (log-rank Mantel-Cox test *p*-value = 0.0049). Similarly, the ART-resistant strain F32-ART5 present a faster recrudescence than the strain F32-TEM after a 48-h treatment with (**3i**) 2.5 µM (log-rank Mantel-Cox test *p*-value = 0.0082) (Figure S49A). This delay in recrudescence time between both strains evidences a cross resistance between (**3i**) and ART on ART-resistant parasites. Moreover, (**3i**) did not present any activity against ART-resistant parasites at the quiescent stage as reported in the quiescent-stage survival assay (QSA) [35]. Indeed, there is no difference in recrudescence times of parasites treated with the combination "DHA / (DHA + **3i**)" and the parasites treated with DHA alone regardless of the concentration used (Figure S49B).

A) Cross resistance evaluation

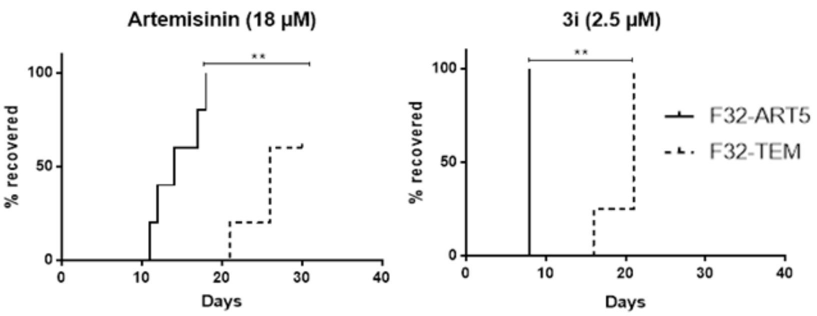

B) Chemosensitivity of quiescent parasites

[3i]	Drug treatment schedule	Post-treatment recrudescence time, in days
**2.5 µM**	DHA 6 h / DHA 48 h	8
	DHA 6 h / DHA + 3i 48 h	10
	Mock 6 h / 3i 48 h	10
**5 µM**	DHA 6 h / DHA 48 h	8
	DHA 6 h / DHA + 3i 48 h	8
	Mock 6 h / 3i 48 h	8

*Figure S49. Evaluation of (3i) on the ART-resistant strain F32-ART5. A) Kaplan-Meier survival analysis of recrudescence assays data of synchronous ring-stage parasites of F32-ART5 (solid lines) and F32-TEM (broken lines) after a 48-h drug exposure. Artemisinin was used as drug control. The final event was defined as the time necessary for P. falciparum cultures to reach the initial parasitemia. Observations were considered censored if no recrudescence was observed at day 30. Log-rank (Mantel-Cox) test was performed (p-value < 0.05 is considered as significant, ** = < 0.01), treatment with 18 µM ART (n = 5) or 2.5 µM 3i (n = 4). B) Recrudescence time of the strain F32-ART5 after treatment of quiescent parasites with 2.5 or 5 µM 3i obtained by QSA. Each experiment was performed once, DHA: dihydroartemisinin (700 nM).*

3. Studying the effect of (3i) on the liver stage development of *P. falciparum*

Cryopreserved primary human hepatocytes were seeded in 96 well plates with a pre-determined seeding dose that gave a single dense layer of attached hepatocytes. Four days post seeding, each plate well was infected with 30,000 freshly extracted *P. falciparum* sporozoites. A dose range (0.9-30 µM) of (**3i**) was added to each well simultaneously with the sporozoites. Atovaquone (ATQ) was used as a positive parasite killing control at 25 nM. Each experimental condition included three culture wells. The culture medium with or without drugs was refreshed every 48 hours and two independent plates were studied. One was fixed at day 6 post-infection which corresponds to almost full maturation of P. falciparum schizonts in hepatocytes. The second plate was fixed at day 12 post-infection to follow the parasites for a longer time having in mind the possible effect of (**3i**) on parasite apicoplast. Upon fixation, parasites were immunostained with anti-HSP70 antibody. Plates were subjected to quantification using an automated plate scanner.

Treatment with (**3i**) had no effect on exoerythrocytic forms (EEF or schizont) size and numbers while ATQ could completely clear all parasites from the culture (Figure S50). The increase in parasite

numbers in 1.8 µM and 3.75 µM could be attributable to a well-known ‘plate effect’ as it has accompanied by a slight higher number of hepatocytes counted in these wells (Figure S50) thus giving a higher infection rate.

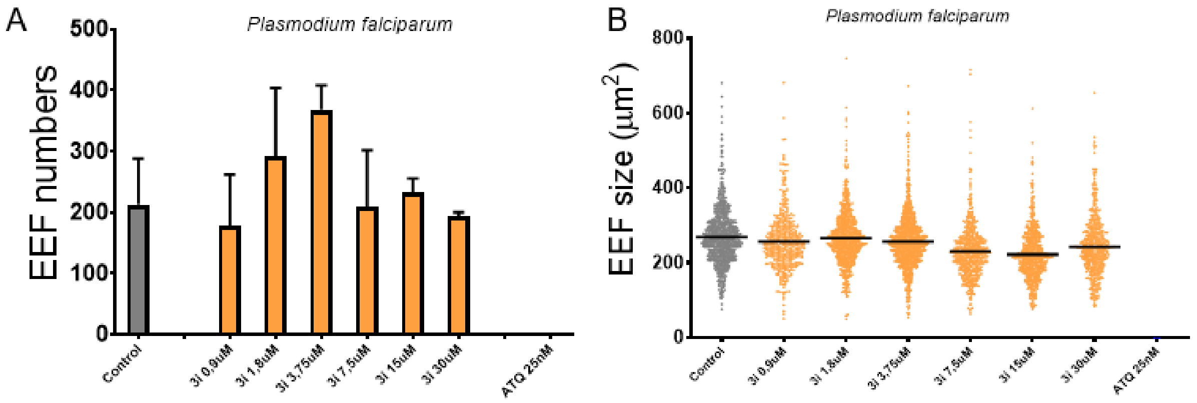

Figure S50. Quantification of P. falciparum hepatic exo-erythrocytic forms (EEFs) number (A) and size (B) in hepatocyte culture plate fixed at day 6 post-infection.

Next, we studied parasites fixed at day 12 post infection. When compared to parasites fixed at day 6, the number of parasites was drastically reduced which is normal since schizogony of *P. falciparum* is 5 to 7 days and at day 12, more than 75% of parasites are liberated from hepatocytes, enter the culture medium and are washed away when culture medium is changed (Figure S51). Concerning (**3i**), we could not observe a significant effect on parasite growth.

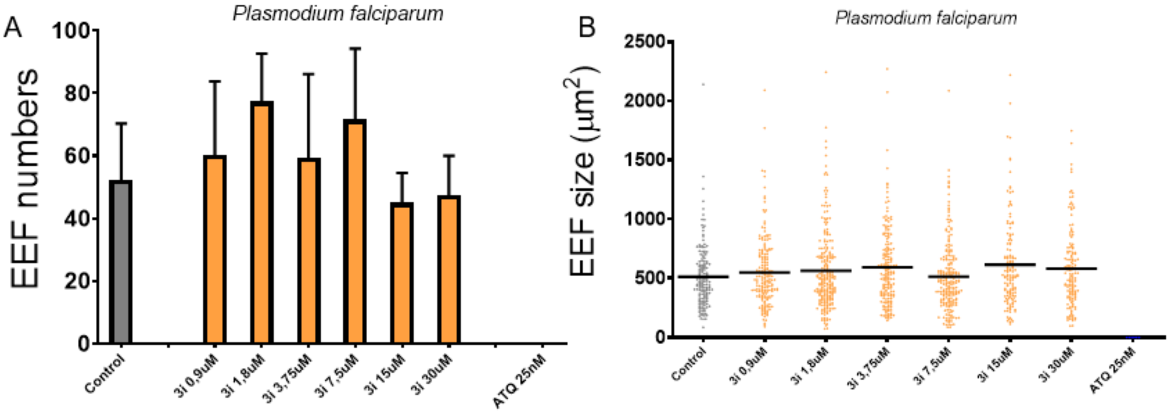

Figure S51. Quantification of P. falciparum hepatic exo-erythrocytic forms (EEFs) number (A) and size (B) in hepatocyte culture plate fixed at day 12 post-infection.

4. The effect of (3i) on apicoplast in liver stage development of *P. falciparum*

Fixed cultures of *P. falciparum* infected primary human hepatocytes were fixed six days following sporozoite infection when intracellular schizonts reach full maturity. Parasite apicoplast was visualized by immunostaining cultures with an antibody that recognizes the acyl carrier protein (ACP). ACP is localized to stroma of apicoplast and is considered a marker for the organelle. Simultaneous counter staining against the heat shock protein 70 of *P. falciparum* (*Pf*HSP70) was used to detect parasites

under a confocal microscope. In full mature non-treated *P. falciparum* the apicoplast is mature and fills the parasite cytoplasm and the ACP signal has a punctate pattern. In parasites exposed to a high dose of 30 µM (**3i**) around 2% of parasites displayed an abnormal apicoplast with loss of ACP signal in the core cytoplasmic regions and its further aggregation towards the periphery (Figure S52).

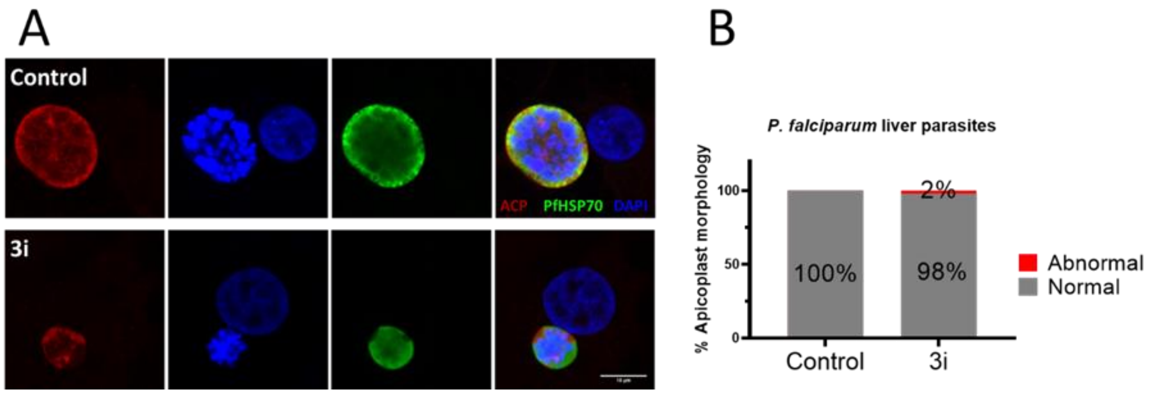

*Figure 52. The effect of **3i** on apicoplast in liver stage development of P. falciparum*
